# Chromatin Accessibility in Cancer: Biological Functions, Mechanisms, Therapeutic Potential, and Future Directions

**DOI:** 10.1002/mco2.70655

**Published:** 2026-03-14

**Authors:** Wentao Xia, Min Jiang, Yefei Huang, Kun Ding, Yansu Chen

**Affiliations:** ^1^ School of Public Health Xuzhou Medical University Xuzhou Jiangsu China; ^2^ Center For Medical Statistics and Data Analysis Xuzhou Medical University Xuzhou Jiangsu China; ^3^ Key Laboratory of Human Genetics and Environmental Medicine Xuzhou Medical University Xuzhou Jiangsu China; ^4^ Department of Urology The Affiliated Hospital of Xuzhou Medical University Xuzhou Jiangsu China

**Keywords:** cancer, chromatin accessibility, epigenetic regulation, therapeutic strategies

## Abstract

Cancer remains a major therapeutic challenge owing to its complex pathogenesis and the limitations of current treatments, such as poor specificity, toxicity, and multidrug resistance. Chromatin accessibility, which is dynamically regulated by genetic, epigenetic, and environmental factors, plays crucial roles in cancer initiation and progression. However, substantial obstacles persist in developing therapeutic strategies that target chromatin accessibility and translating them into clinical practice. This review comprehensively summarizes the biological functions and regulatory mechanisms of chromatin accessibility in tumors, encompassing tumorigenesis, progression, metabolic reprogramming, angiogenesis, stemness, tumor immune microenvironment, and therapy resistance. We integrate comparisons between human and murine models and detail key profiling technologies, including Assay for Transposase‑Accessible Chromatin with high‑throughput sequencing, DNase‑seq, single‑cell multiomics, and three‐dimensional chromatin‑conformation assays. Furthermore, we compile recent preclinical and clinical trials that utilize chromatin accessibility as a biomarker or therapeutic target, along with combination strategies involving chemotherapy, immunotherapy, targeted therapy, and radiotherapy. From a multiomics and interdisciplinary perspective, we discuss current limitations in translating fundamental research into clinical applications and highlight future directions for epigenetics‑based precision oncology.

## Graphical abstract

Cancer remains the leading cause of mortality worldwide, and drug resistance further underscores the urgent need for innovative therapeutic strategies. Chromatin, a stable yet highly dynamic nucleoprotein complex, serves as the primary carrier of genetic material in eukaryotic cells. Chromatin accessibility reflects the degree of openness of chromatin structure, determining the capacity of DNA to interact with transcription factors, regulatory proteins, and other molecules. The dynamic relationship between chromatin accessibility and gene transcription activity represents a core mechanism of gene expression regulation. Regulated by multiple epigenetic mechanisms, chromatin accessibility has attracted significant research attention for its critical role in tumor initiation, progression, and treatment response.

Unlike previous reviews on chromatin accessibility, which primarily characterized its landscapes and regulatory mechanisms or its broad pathological roles in human diseases, a systematic integration of chromatin accessibility with tumor biology and therapeutic strategies remains underexplored. This review synthesizes the latest research to analyze the progress and limitations in current chromatin accessibility studies, comprehensively assessing the research landscape. We specifically focus on exploring: (1) the key epigenetic mechanisms governing chromatin accessibility during tumorigenesis and progression; (2) its functional impact in tumors; and (3) current key strategies and emerging technologies in tumor therapy, including a compiled list of representative epigenetic drugs based on different targets for cancer treatment and their associated clinical trials in combination therapies. We also explore the feasibility and novel perspectives of integrating multiomics approaches with artificial intelligence to advance epigenetics‐based precision tumor therapy.

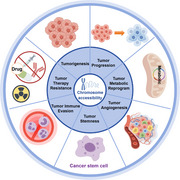

## 1 Introduction

Cancer continues to pose a significant therapeutic challenge, primarily attributed to its intricate pathogenesis and the inherent limitations of current treatment modalities—including poor specificity, systemic toxicity, and the development of multidrug resistance [1, 2, 3]. Beyond genetic mutations, acquired epigenetic abnormalities have emerged as key drivers of oncogenic gene expression programs and the hallmarks of tumor biology [4, 5, 6, 7]. The term “epigenetics,” first coined by Conrad Waddington, broadly describes heritable regulatory mechanisms governing gene activity without altering the DNA sequence, encompassing DNA methylation, histone modifications, chromatin remodeling, and noncoding RNA (ncRNA) regulation [4, 5]. As a central pillar of epigenetic regulation, chromatin accessibility—defined as the degree of physical openness of chromatin, which determines the binding capacity of transcription factors, regulatory proteins, and other molecules—plays a pivotal role in defining cell identity and function [8]. Its dynamic regulation integrates genetic, epigenetic, and environmental cues, making it a pivotal interface in cancer development and progression.

The study of chromatin accessibility has a long‐standing history, tracing back to mid‐20th century observations of nuclease‐sensitive genomic regions, which first hinted at structurally open chromatin associated with active transcription. Milestone discoveries have progressively unveiled the multifaceted mechanisms governing this accessibility. These include the establishment of histone acetylation as a marker linked to increased chromatin openness and gene activation [9], the elucidation of DNA methylation in promoter regions as a mechanism for silencing gene expression by reducing accessibility (notably, intragenic DNA methylation is typically associated with gene activation) [10], and the characterization of how three‐dimensional (3D) chromatin architecture (e.g., loops and topological‐associated domains [TADs]) modulates the proximity of regulatory elements to influence accessibility and gene expression [11]. Chromatin accessibility is thus coordinately regulated by an integrated network involving DNA methylation, histone modifications, chromatin remodeling complexes (e.g., switch/sucrose nonfermentable [SWI/SNF]), transcription factors, ncRNAs, and higher‐order chromatin folding [9, 10, 11]. These findings underscore the crucial role of chromatin accessibility in epigenetic regulation, which in turn dictates gene expression patterns in cells. Advances in profiling technologies, particularly Assay for Transposase Accessible Chromatin with high‐throughput sequencing (ATAC‐seq) [12], now enable genome‐wide mapping of these dynamics, revealing widespread alterations in tumors that influence oncogenesis, metastasis, metabolic reprogramming, and therapy resistance [13, 14, 15, 16, 17, 18, 19].

Despite the growing recognition of its importance, previous reviews have often focused either on the general landscapes and regulatory mechanisms of chromatin accessibility [20] or its broad pathological roles across human diseases [21]. A systematic and comprehensive integration of chromatin accessibility with the hallmarks of cancer biology and, crucially, with translational therapeutic strategies (spanning from mechanistic insights to clinical trial evidence), remains underexplored. This critical knowledge gap provides the rationale for the present review.

This review aims to synthesize the latest research to provide a comprehensive overview of how chromatin accessibility shapes tumor biology and can be leveraged for cancer treatment. We will specifically explore: (1) the key epigenetic mechanisms governing chromatin accessibility during tumorigenesis and progression; (2) its functional impact across various tumor processes including metabolism, angiogenesis, stemness, immunity, and therapy resistance; and (3) current and emerging therapeutic strategies, compiling representative epigenetic drugs and their associated clinical trials when used in combination with other modalities. Furthermore, we will examine the utility of chromatin accessibility as a diagnostic and prognostic biomarker.

To address these objectives, the review is structured as follows. We first outline the biological basis of chromatin accessibility, covering its structural foundations, key regulatory mechanisms, and core profiling technologies. We then detail its multifaceted roles in tumors, discussing its contributions to tumorigenesis, progression, and the tumor microenvironment. Subsequently, we compile and analyze treatment strategies and clinical trials involving chromatin accessibility, reviewing its use as a biomarker and a therapeutic target in various combination regimens. Finally, from a multiomics and interdisciplinary perspective, we discuss current challenges in clinical translation and propose future directions for epigenetics‐driven precision oncology. This logical progression from fundamental concepts to biological functions, and ultimately to therapeutic applications, is designed to offer a cohesive and insightful resource for both researchers and clinicians in the field.

## 2 Biological Basis of Chromatin Accessibility

This section lays the foundational framework for understanding chromatin accessibility in the context of cancer. We begin by delineating the fundamental concepts of chromatin architecture and the principle of accessibility. We then dissect the sophisticated, multilayered regulatory network governing its dynamics. Finally, to bridge mechanistic understanding with empirical research, we detail the pivotal experimental and computational technologies that enable the mapping and interpretation of chromatin accessibility landscapes.

### 2.1 Chromatin Structure and Accessibility

#### 2.1.1 The Basic Structure of Chromatin

Chromatin is a stable yet highly dynamic nucleoprotein complex composed of DNA, histones, nonhistone proteins, and small amounts of RNA, serving as the primary carrier of genetic material in eukaryotic cells [22]. The fundamental structural unit of chromatin is the nucleosome, formed by 147 base pairs of DNA wrapped around a histone octamer. This octamer consists of an H3–H4 histone tetramer and two H2A–H2B dimers. Chromatin plays a crucial role in genome compaction while dynamically regulating various nuclear processes, with nucleosomes constituting the core regulatory machinery [23, 24]. We will subsequently elaborate on the multifaceted regulatory functions of nucleosomes in these biological processes.

#### 2.1.2 Characterization of Open and Closed Regions of Chromatin

Chromatin accessibility—a critical property defined by the permissibility of physical interactions between macromolecular complexes and chromatin DNA—fundamentally reflects the openness of chromatin architecture. Within the nucleus, chromatin exists in a spectrum of accessibility states: (1) highly accessible (“open chromatin”) characterized by nucleosome‐depleted regions, (2) moderately accessible (“permissive chromatin”) with dynamic nucleosome repositioning, and (3) low‐accessibility/repressive (“closed chromatin”) marked by stable nucleosome occupancy [8]. Open chromatin regions exhibit decompacted structures with exposed DNA, predominantly localizing to gene promoters, enhancers, and cis‐regulatory elements to facilitate transcriptional activation. In contrast, closed chromatin adopts condensed configurations with limited DNA exposure, typically occupying transcriptionally silent loci and heterochromatic regions. Under specific circumstances, open chromatin region can also promote gene repression (not only promote gene activation), for example, opening of insulator or repressor regions can promote nearby gene's silencing. The opposite scenario also holds true [25]. This structural dichotomy between accessible and inaccessible chromatin provides critical mechanistic insights into the relationship between chromatin topology and epigenetic regulation, which will be systematically explored in subsequent sections.

#### 2.1.3 Relationship Between Chromatin Accessibility and Gene Expression

Chromatin accessibility is primarily influenced by the spatial distribution and occupancy patterns of nucleosomes and other DNA‐binding factors [26]. This accessibility determines the availability of DNA for interactions with transcription factors, regulatory proteins, and RNA polymerase, thus directly regulating transcriptional activity. Notably, accessible chromatin regions constitute merely 2–3% of the genome, with over 90% of these regions remaining unoccupied by transcription factors under basal conditions [27]. Transcription factors are a class of proteins that can bind to specific sequences of DNA. In regions of open chromatin, where DNA is more exposed, transcription factors can readily access and bind to key regulatory sequences, such as promoters and enhancers. For pioneer transcription factors, they can access the nucleosome occupied regions directly, thereby initiating or enhancing gene transcription [8]. Conversely, in closed chromatin regions, limited DNA exposure hinders transcription factor binding, effectively repressing gene transcription [28]. Thus, it is easy to see that the relationship between dynamic changes in chromatin accessibility and transcription factor binding and gene transcriptional activity as one of the central mechanisms of gene expression regulation.

### 2.2 Mechanisms Regulating Chromatin Accessibility

**FIGURE 1:**
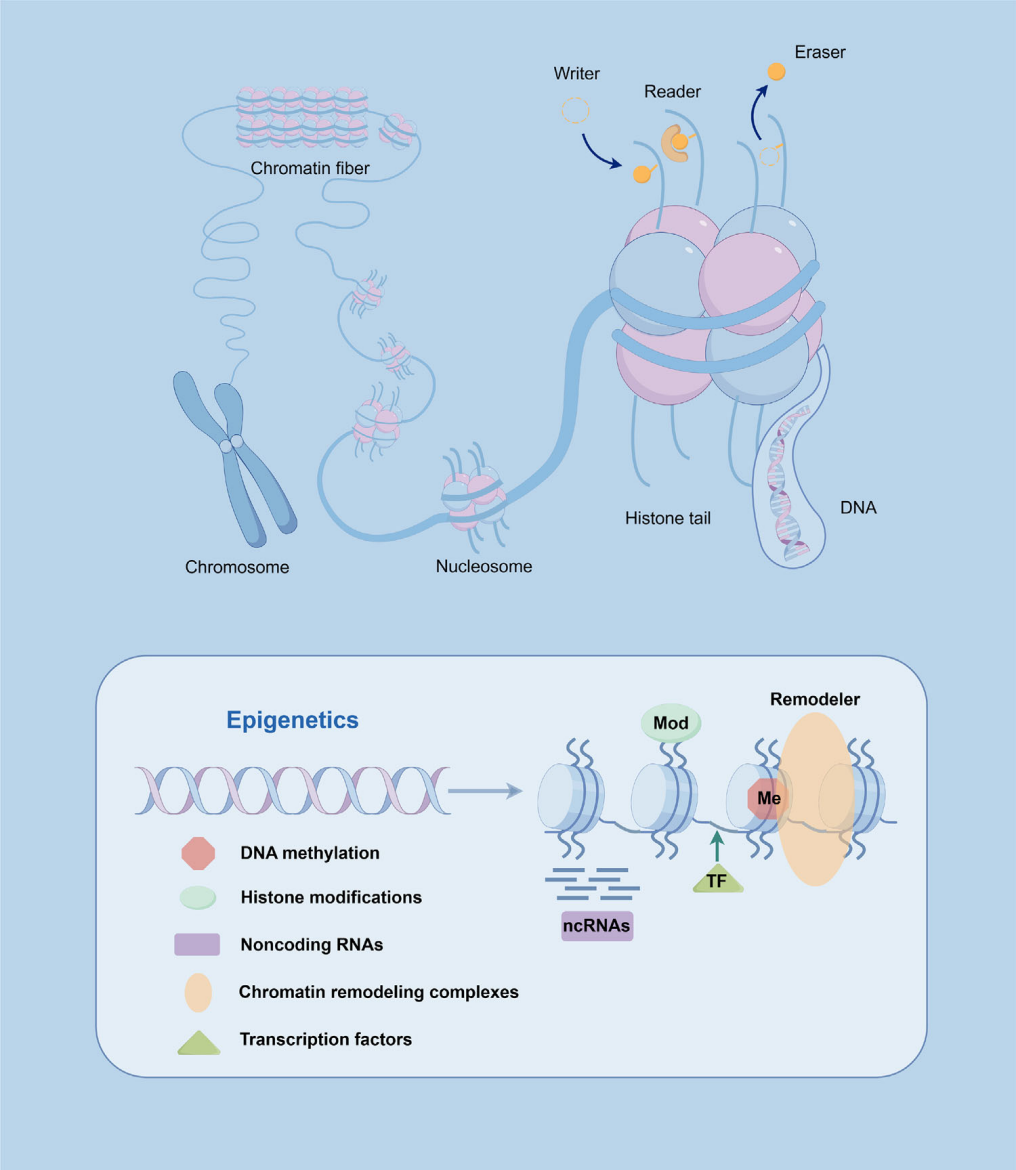
Basic chromatin structure and regulatory mechanisms. The basic structure of chromatin and the mechanisms by which it is subject to multiple epigenetic regulations, including DNA methylation, histone modifications, noncoding RNAs, chromatin remodeling complexes, and transcription factors.

Chromatin accessibility is dynamically regulated through an integrated network of epigenetic mechanisms, including DNA methylation, histone modifications, ncRNAs, chromatin remodeling complexes, transcription factors, and chromatin 3D structure. In this section, we systematically delineate the principal mechanisms governing chromatin accessibility and their coordination in transcriptional control (Figure 1).

#### 2.2.1 DNA Methylation

DNA methylation is a crucial epigenetic modification mechanism that directly regulates gene expression by adding methyl groups to the DNA molecule. This process typically occurs initially on cytosine–phosphate–guanine (CpG) islands within the promoter regions of genes. This modification can silence genes or increase mutation probability by deaminating 5‐methylcytosine (5mC) to 5mU, which later will be repaired as T. DNA methylation is primarily catalyzed by three enzymes, DNMT1, DNMT3A, and DNMT3B. DNA methyltransferase (DNMT) mediates the addition of a methyl group to the fifth carbon of the cytosine base to form 5mC. DNMT3A and DNMT3B are the enzymes responsible for ab initio DNA methylation, while DNMT1 is the enzyme necessary to maintain DNA methylation during DNA replication [29, 30]. Methylation at the 5‐carbon (5mC) of cytosine ring in CpG dinucleotides was the first identified form of epigenetic modification and remains the most extensively studied chromatin modification [31, 32, 33]. DNA methylation plays a crucial role in epigenetic regulation and is involved in a variety of nuclear processes such as gene expression, DNA repair, and recombination [34, 35].

DNA methylation affects chromatin accessibility through multiple mechanisms. First, DNA methylation alters the structural properties of DNA, including geometric conformation, mechanical stability, and physicochemical properties [36, 37, 38], and it should be noted that the magnitude of the effects induced by CpG methylation is highly dependent on the local DNA sequence environment around the methylation site [39]. Second, numerous studies have demonstrated that DNA methylation‐induced changes in DNA geometry and mechanical properties offer the prospect of gaining insight into how DNA methylation regulates nucleosome structure and dynamics [37, 40, 41].

Emerging evidence highlights the context‐dependent role of DNA methylation in regulating nucleosome positioning and transcription. However, findings remain inconsistent across different investigations [42]. A subset of investigations demonstrates that unmethylated CpG‐rich promoter regions exhibit nucleosome depletion, whereas DNA hypermethylation correlates with increased nucleosome occupancy [43, 44, 45]. Conversely, alternative studies report an inverse association, where CpG methylation destabilizes nucleosome–DNA interactions and promotes nucleosome eviction [38, 46, 47]. A possible explanation for these contradictory results may lie in the existence of nucleosomes located in different genomic regions, which may respond differently to DNA methylation. DNA methylation of promoter regions and CpG islands usually leads to closing of open chromatin regions and gene silencing. But DNA methylation of gene body regions usually connects to gene activation [43, 48]. Finally, DNA methylation is also involved in the regulation of the 3D structure of chromatin, and most studies have shown that DNA methylation can be recognized by reader proteins, leading to gene silencing and chromatin compression [10, 38]. In conclusion, DNA methylation can significantly affect the physicochemical properties of DNA in a sequence‐dependent manner, inducing changes in nucleosome stability and DNA packaging that ultimately modulate DNA accessibility.

Extensive research has unequivocally established DNA methylation as a pivotal epigenetic hallmark of carcinogenesis, characterized by two complementary aberrations: locus‐specific hypermethylation at CpG islands within tumor suppressor gene (TSG) promoters and genome‐wide hypomethylation promoting chromosomal instability (CIN) [1, 49]. In cancer cells, the predominant alteration is the hypermethylation of TSGs, leading to their inactivation and promoting tumor growth. In contrast, activation of prometastatic genes induced by DNA hypomethylation promotes tumor invasion and metastasis [50]. Hypermethylation‐mediated silencing of TSGs is a dominant mechanism in cancer. For instance, Sun et al. found that hypermethylation disrupts TP53 binding to the ZNF334 promoter, suppressing its transcription and accelerating hepatocellular carcinoma (HCC) progression [51]. Similarly, Yellow et al. identified hypermethylation by DNMT1 as a driver of triple‐negative breast cancer (TNBC). This process suppresses estrogen receptor (ER) expression, induces epithelial–mesenchymal transition to enable metastasis, and enhances autophagy and cancer stem cell (CSC) proliferation in TNBC [52].

Global DNA hypomethylation is a hallmark of CIN in aggressive cancers [53]. Endo et al. linked genome‐wide hypomethylation to poor prognosis and occult metastasis in pancreatic cancer, suggesting its utility as a predictive biomarker [54]. In small cell lung cancer (SCLC), Na et al. found that DNMT3A downregulation—mediated by KMT2C loss—induces hypomethylation of prometastatic genes, facilitating tumor dissemination [55]. Guo et al. further showed that DNA hypomethylation silences antitumor immune genes in early prostate cancer (PC) while retaining proproliferative drivers, enabling immune evasion during metastasis [56].

DNA methylation intersects with key cancer pathways. Han et al. found that PHF14‐mediated hypermethylation of SMAD7 activated TGF‐β signaling to drive lung adenocarcinoma (LUAD) metastasis [57]. In colorectal cancer (CRC) with KRAS mutations, Huang et al. found that SLC25A22‐mediated glutamine catabolism reduced DNA demethylation, which enhanced Wnt/β‐catenin signaling and promoted tumorigenesis and cancer stemness [58]. Collectively, these findings underscore DNA methylation's indispensable role in shaping tumor biology through chromatin remodeling and pathway dysregulation.

#### 2.2.2 Histone Modifications

Histones are core proteins essential for regulating processes such as DNA packaging, chromatin acquisition, gene expression, and DNA repair [59]. Posttranslational modifications (PTMs) of histones—including acetylation, methylation, phosphorylation, SUMOylation, and ubiquitination—dynamically modulate chromatin structure and function. These modifications occur predominantly on the N‐terminal tails of histones and influence nucleosome stability, chromatin accessibility, and recruitment of effector proteins [60]. Histone modifications are key epigenetic mechanisms that regulate chromatin accessibility and gene expression, dynamically regulating chromatin accessibility by altering chromatin structure and recruiting effector proteins. Transcriptional activation or repression of genes is affected by aberrant histone modifications, which also affect many processes, including DNA replication and recombination, thereby impairing cellular homeostasis and controlling tumor formation [61, 62]. Histone tails and cores undergo a variety of PTMs, including acetylation, phosphorylation, methylation, SUMO acetylation, and ubiquitination. These PTMs can establish different chromatin environments that regulate a variety of nuclear processes such as gene expression, replication, repair, and regulation of genome structure [63]. In this section, we will briefly describe how several important and well‐studied PTMs (acetylation, methylation, and phosphorylation) affect chromatin accessibility in open and closed chromatin conformations [59, 64].

##### 2.2.2.1 Histone Acetylation

Histone acetylation, a hallmark epigenetic modification, is catalyzed by histone acetyltransferases (HATs), which transfer acetyl groups to lysine residues on histone N‐terminal tails [65]. Chromatin dynamics can be determined by nucleosome stability, which can be directly affected by histone core structural domain acetylation [66]. Histone acetylation significantly improves chromatin accessibility by neutralizing histone positive charge and attenuating its interaction with negatively charged DNA, leading to loosening of chromatin structure [67]. For example, Kouzarides et al. found that elevated acetylation enhances DNA accessibility, enabling transcriptional machinery to engage with target genes [62]. Similar claims were validated in a later study by Mathias Wenes, who found that mitochondrial pyruvate carrier (MPC) inhibition‐induced metabolic flexibility promotes acetyl coenzyme‐A production from glutamine and fatty acid oxidation, thereby enhancing histone acetylation and chromatin accessibility on promemory genes [68]. Scholars such as Sheu et al. found that cilium 5‐HTR6 stimulation activates the nonclassical Gαq/11–RhoA pathway, which regulates nuclear actin and increases histone acetylation, thereby increasing chromatin accessibility [69]. Histone acetylation dysregulation is increasingly implicated in tumorigenesis. He et al. highlighted acetyl‐CoA's role as a metabolic bridge, connecting lipid metabolism to histone acetylation to fuel cancer growth, proliferation, and metastasis [70]. Miziak et al. emphasized that aberrant acetylation patterns alter chromatin architecture and gene expression, serving as potential biomarkers for cancer progression and prognosis [71]. Thus, these studies underscore histone acetylation's dual role as a regulator of chromatin structure and a driver of oncogenic processes.

##### 2.2.2.2 Histone Methylation

Histone methylation represents a fundamental epigenetic modification primarily occurring on lysine (K) and arginine (R) residues of histones H3 and H4. This dynamic process is mediated by histone methyltransferases (HMTs) and reversed by demethylases (KDMs). Unlike acetylation, methylation does not alter histone charge but regulates chromatin state by recruiting specific effector proteins [72]. Histone methylation of lysine 4 (H3K4me3), the most extensively studied modification, marks transcriptionally active chromatin regions [73]. Methylation of cytosine in CpG dinucleotides, histone lysine and arginine residues has been suggested by Li et al. to be a chromatin modification that plays a key role in regulating genome integrity, replication, and accessibility [74]. Posttranslational methylation of histone lysine or arginine residues plays an important role in gene regulation and other physiological processes. Aberrant histone methylation patterns resulting from genetic alterations (mutations, translocations, or gene overexpression) are strongly implicated in disease pathogenesis, particularly cancer [75]. Enhancer of Zeste Homolog 2 (EZH2) overexpression correlates with poor overall survival (OS) in lung cancer patients [76]. Other recent studies have found that abnormal histone methylation levels contribute to skin tumorigenesis and summarized the efficacy of several epigenetic inhibitors targeting histone methylation‐modifying enzymes in skin cancer, suggesting that histone methylation‐modifying enzymes could serve as a new class of targets for skin cancer therapy [77].

##### 2.2.2.3 Histone Phosphorylation

Histone phosphorylation is a dynamic PTM regulated by the coordinated activity of protein kinases (e.g., Aurora B, MSK1/2) and phosphatases (e.g., PP1/PP2A). Predominantly occurring on serine/threonine residues of histones H3 and H2A, this modification modulates chromatin structure and function during critical cellular processes such as transcription and mitosis [78]. It has been shown that phosphorylation of histone H3 is unique because it binds to open chromatin during gene activation on the one hand and marks highly condensed chromatin during mitosis on the other [79]. In tumorigenesis, phosphorylation of serine 10 on histone H3 (H3S10ph) is emerging as an important player in cancer development and dissemination as it promotes malignant transformation of cells and is involved in essential cellular functions [80]. Large‐scale proteogenomic analyses have further identified pan‐cancer patterns linking phosphorylation to dysregulated DNA repair and acetylation to altered metabolic–immune crosstalk, revealing distinct tumor subpopulations with shared epigenetic vulnerabilities [81].

Histone modifications are critical regulators of chromatin structure, gene expression, and tumorigenesis. In addition to the above three histone modifications, there are a variety of less prevalent and atypical PTMs, such as ubiquitination, lactylation, succinylation, citrullination, ADP‐ribosylation, 5‐hydroxytryptophanization, and serotoninization. For example, posttranslational histone modifications are one of the mechanisms used by cellular processes to remodel chromatin and gain access to potential DNA templates [82], and deletion of the deubiquitinating enzyme BAP1 promotes H2AK119ub modification, remodeling the accessibility of chromatin and thus disrupting transcriptome patterns in human liver‐like organs [83]. Merkuri et al. demonstrated that glycolysis induced histone lactylation of neural crest‐associated genes, thereby increasing their chromatin accessibility, combining metabolic state of embryonic cells with chromatin organization and gene regulatory network activation [84]. Xu et al. found that histone lactylation plays a crucial role in cancer progression and that anaerobic metabolism promotes breast cancer survival through histone‐3 lysine‐18 lactylation mediating the PPARD axis [85]. Upon study, Wang et al. found that Zeb1 in epithelial‐like cells transcriptionally regulate the expression of several key glycolytic enzymes, thereby predisposing tumor cells to utilize glycolysis for energy metabolism. In the process, lactate accumulation‐mediated histone lactylation enhances chromatin accessibility and cellular plasticity, including induction of neurogenic gene expression, thereby promoting neuroendocrine PC (NEPC) development [86]. Jing et al. found that lysine succinylation (Ksucc) is a newly identified histone PTM and that this succinylation affects nucleosome dynamics and is important in regulating DNA accessibility and chromatin dynamics [87]. Kamo et al. found that the citrullination at R53 in H1.2 resulted in the reduced electrostatic interaction with DNA and the reduced binding affinity to nucleosomes [88]. Smith et al. demonstrated that histone poly(ADP‐ribosylation) factor 1‐dependent histone ADP‐ribosylation triggers chromatin relaxation to facilitate the recruitment of repair factors at DNA damage sites [89]. Similarly, Martinez‐Zamudio et al. proposed that PARP‐1 enzyme activity promotes gene transcription by increasing promoter accessibility through histone ADP‐ribosylation [90]. Recently, it has also been found that histone 5‐hydroxytryptophanylation, serotoninylation may also be a potential marker of chromatin activity [91, 92]. In conclusion, PTM is an integral part of tumor cell adaptation and response to intracellular and environmental changes, and more in‐depth studies are needed on the PTM control processes that lead to cancer development and progression.

Regulation of histone proteins affects gene expression through multiple mechanisms including exchange with histone variants. Beyond PTMs, the selective incorporation of histone variants represents another critical mechanism governing chromatin structure and accessibility. Histone variants (e.g., H3.3, H2A2, H2BE) differ in amino acid sequence from their canonical counterparts and are often incorporated into chromatin in a DNA replication‐independent manner, conferring unique biophysical properties to the nucleosome. These variants directly remodel the chromatin accessibility landscape by altering nucleosome stability and histone–DNA interactions [93, 94]. In cancer, the dysregulated expression of histone variants is a frequent event. They drive gene expression programs linked to malignant phenotypes by promoting or restricting specific chromatin states. For instance, in glioblastoma (GBM), histone variant macroH2A2 shapes chromatin accessibility at enhancer elements to antagonize transcriptional programs of self‐renewal [95]. In addition, Filipescu et al. report that macroH2A deficiency in cancer‐associated fibroblasts leads to altered chromatin looping and elevated inflammatory gene expression, thereby affecting immune cell function and limiting the antitumor response in melanoma [96]. Consequently, acting as “oncohistones,” histone variants play a deterministic role in tumorigenesis and progression by establishing unique patterns of chromatin openness.

#### 2.2.3 Noncoding RNAs

ncRNAs, broadly defined as RNA transcripts not translated into functional proteins, have emerged as pivotal regulators of chromatin dynamics and gene expression. Advances in transcriptome‐wide analyses have expanded the catalog of ncRNAs, revealing their diverse roles in epigenetic modulation [97]. ncRNAs can be broadly categorized into small (<200 nucleotides) and long (>200 nt) ncRNAs. Small ncRNAs include endo‐siRNAs, microRNAs, piwi‐interacting RNAs, small nuclear RNAs, small nucleolar RNAs, and tRNA‐derived fragments [98, 99]. Among these, siRNAs and miRNAs are known to have regulatory roles in epigenetics [100]. SiRNAs are derived from double‐stranded RNA precursors that are cleaved by DICER to form 19 to 24 base RNAs, and studies have shown that siRNAs act through the RNA interference pathway to silence gene expression through DNA methylation and histone modification [101]. MiRNAs are short ncRNAs (∼21 nt) that act as posttranscriptional regulators with a large number of targets [102]. Shui et al. used ATAC‐seq to analyze the chromatin accessibility landscape of colon tissues expressing K‐RasWT and K‐RasG12D, and the data showed that overactivation of K‐Ras induced a significant increase in K‐Ras expression. Ras overactivation induces full de‐repression of miRNA targets that is dependent on miRNA expression levels [103].

It is well known that many lncRNAs play regulatory roles in cell growth, development, and disease processes. Lu et al. identified a long‐chain ncRNA called lncRNA Muscle Regeneration Enhancer Factor, which interacts with Smarca5 to promote chromatin accessibility when muscular satellite cells are activated and begin to differentiate, thus facilitating the p300/CBP/H3K27ac genomic binding [104]. Terroba et al. found an increase in overall chromatin accessibility upon overexpression of lncRNA metastasis‐associated LUAD transcript 1 (MALAT1), suggesting that individual lncRNAs can drive LUAD metastasis through reprogramming of the tumor microenvironment [105]. Deng et al. identified a mechanism of chromatin accessibility and gene transcriptional regulation jointly mediated by RNA m6A formation and DNA demethylation, highlighting the importance of the interplay between RNA m6A and DNA modifications in physiological and pathogenic processes [106]. Similarly, Li et al. revealed through their study that the CFL1–METTL3–seRNA m6A–YTHDC2/MLL1 axis plays a role in the epigenetic regulation of local chromatin status and gene expression [107]. Confirmed by a growing body of evidence, we can assume that regulatory ncRNAs play an important role in epigenetic control.

Beyond the sequences of ncRNAs themselves, their posttranscriptional chemical modifications—core components of epitranscriptomics—represent an additional critical layer of gene expression regulation. Key RNA modifications (e.g., m6A, m5C, m3C) dynamically and reversibly regulate RNA fate, including stability, splicing, nuclear export, and translation efficiency, through dedicated “writer,” “eraser,” and “reader” proteins [108]. Notably, profound crosstalk exists between this RNA‐level regulation and chromatin structure. For instance, Li et al. demonstrate the cofilin family protein CFL1 as a METTL3 cofactor that helps super‐enhancer (SE) RNA m6A methylation formation. Then SE RNA m6A promotes local chromatin accessibility and oncogene transcription in pancreatic ductal adenocarcinoma (PDAC) [109]. Furthermore, Lee et al. report that METTL8 links mt‐tRNA m3C modification to the HIF1α/RTK/Akt axis to sustain GBM stemness and tumorigenicity [110]. Thus, RNA modifications, along with their potential interactions with chromatin regulators, fine‐tune chromatin accessibility and gene expression programs.

#### 2.2.4 Chromatin Remodeling Complexes

Chromatin remodeling complexes are a class of ATP‐hydrolysis‐dependent molecular machines that dynamically regulate chromatin accessibility by altering nucleosome position and composition [111, 112]. Chromatin remodelers were originally discovered and validated in yeast, and eukaryotic cells contain four chromatin remodeling complexes that are classified based on similarities and differences in ATPase subunits, including SWI/SNF, Imitation Switch (ISWI), Chromodomain Helicase DNA binding domain (CHD), and inositol requiring 80 (INO80) [113].

##### 2.2.4.1 SWI/SNF Complexes

The SWI/SNF complex is the most studied chromatin remodeling complex in mammals and has been found to be involved in a wide variety of life activities [114]. The SWI/SNF complex can be divided into three major modules—ATPase, actin‐related protein (ARP), and somatic module [115]. The SWI/SNF chromatin remodeling complex (SCRC) is a subfamily of ATP‐dependent chromatin remodeling proteins that play a wide range of roles in the regulation of gene expression by modifying chromatin structure [116]. The SCRC acts by displacing nucleosomes near important regulatory sites, which promotes the binding of transcription factors, thereby facilitating the expression of genes and gene activation in unicellular and multicellular eukaryotes [117, 118]. Genomic abnormalities of the SCRC subunit occur in approximately 20% of cancers [119, 120]. The first clue linking the SWI/SNF complex to cancer appeared in the late 1990s, when mutations in the gene encoding the SMARCB1 subunit were identified in rhabdomyosarcoma [121]. Additional studies have supported this idea, and inactivating mutations in AT‐rich interaction domain 1A (ARID1A) are prevalent in a wide range of cancers, including up to 62% of clear cell carcinomas of the ovary [122], endometrioid carcinomas [123], and gastrointestinal tumors such as gastric, colorectal, and pancreatic cancers [124]. The mechanisms by which mutations in each individual subunit promote tumorigenesis and the function of mutant SWI/SNF complexes in cancer is currently an active area of research, and in addition to this, a number of studies have elucidated the pathways regulated by the SWI/SNF complexes, and how subunit mutations that disrupt the expression programs of these genes can promote cancer [125]. Concepcion et al. found that SMARCA4/BRG1 encodes one of the two mutually exclusive ATPases present in the mammalian SCRC, which is frequently mutated in lung cancer and drives cancer progression, leading to an increased incidence of development and metastasis of highly complex undifferentiated malignancies [126]. Therefore, we can assume that aberrant expression and mutation of the SWI/SNF complex is prevalent in cancer and that it plays a broad role in regulating gene expression by modifying chromatin accessibility and structure.

##### 2.2.4.2 ISWI Complexes

It has been shown that Smarca5 (also known as Snf2h), an important enzyme in the SWI/SNF family with remodeling activity, alters gene expression by promoting chromatin accessibility, and a transposase‐accessible chromatin analysis of zebrafish and newborn fetuses has shown that Smarca5 is responsible for maintaining chromatin accessibility to promoters of hematopoiesis‐related genes in fetal HSPCs. Smarca5 interacts with nucleolin to promote chromatin remodeling, which in turn promotes genomic binding of transcription factors to regulate expression of hematopoietic regulators such as bcl11ab [127]. The ISWI family is an important component of ATP‐dependent chromatin remodeling complexes and consists of two ATPases, SNF2L (SMARCA1) or SNF2H (SMARCA5), which alternatively bind to complex‐specific auxiliary subunits [128]. Deletion of SNF2H in mammalian cells results in genome‐wide changes in nucleosome organization, accompanied by an increase in nucleosome repeat length and a decrease in the binding of specific transcription factors (e.g., CCCTC‐binding factor [CTCF]), which coincides with reduced chromatin accessibility [129, 130]. Unlike the related SCRC, the ISWI complex fails to evict nucleosomes, but instead regulates nucleosome sliding to maintain appropriately spaced nucleosome arrays, which dynamically affects chromatin accessibility [131, 132]. Iurlaro et al. investigated this and found that the core subunit of the nucleosome remodeling factor nucleosome remodeling factor (NURF), bromodomain PHD finger transcription factor (BPTF) leads to a strong reduction in chromatin accessibility and SNF2H ATPase localization around CTCF sites. The study further revealed a mechanistic link between NURF‐mediated chromatin remodeling and the structural function of CTCF. [133]. High‐throughput sequencing and a growing number of basic and clinical studies have identified altered function or composition of ISWI‐containing complexes as critical for tumorigenesis and progression. Genetic abnormalities are major determinants of the levels of certain ISWI subunits in specific types of cancer and contribute to the tumor phenotype [134]. For example, Buganim et al. identified BPTF as a gene involved in transcriptional regulation and chromatin remodeling and showed that BPTF may play a procarcinogenic role in tumors carrying chromosomal aberrations in 17q [135]. Itamochi et al. obtained tumor from 55 Japanese women diagnosed with ovarian clear cell carcinoma (OCCC). Tissue samples and matched blood samples were obtained from 55 Japanese women diagnosed with OCCC, and whole‐genome sequencing using the Illumina HiSeq platform revealed that mutations in the ISWI ATPase SNF2L (SMARCA1) were associated with OCCC [136]. In addition, some ISWI subunits were strongly associated with patient prognosis. In HER2+ breast tumors, high levels of BAZ1A are associated with deleterious recurrence‐free survival (RFS) and very poor OS [137]. Pietrzak et al. found that TIP5 expression and high PTEN‐del expression in prostate tumors were strongly associated with reduced prostate‐specific antigen RFS [138]. Dai et al. found that BPTF was highly expressed in non‐small cell lung cancer (NSCLC) tumor tissues, and BPTF cooperated with p50 NF‐κB to promote COX‐2 expression and tumor cell growth in lung cancer and was positively associated with advanced clinical stage, more lymph nodes, and distant metastasis [139]. This series of studies on ISWI has also demonstrated that it influences tumorigenesis and development through its involvement in transcriptional regulation and chromatin remodeling.

##### 2.2.4.3 CHD Complexes

CHD proteins are ATP‐dependent chromatin modifiers involved in the structural organization of chromatin and act as gatekeepers for genome access [140]. The hallmark feature of CHD remodelers is the presence of a bichromatin structural domain in its N‐terminal region and SNF2‐like ATPase/helicase core, which mediates binding to chromatin by directly binding to the histone H3 tails to mediate binding to chromatin through direct binding to methylated lysines [141]. The CHD family is a family of chromatin regulators that are frequently lost or inactivated in a variety of human cancers, and CHD proteins affect chromatin compression and thus access to DNA by cellular mechanisms. The CHD family consists of nine members, CHD1–9, with CHD1 serving as the founding member of the CHD family, was originally discovered to be a DNA‐binding protein. It has been shown that yeast Chd1 is primarily responsible for chromatin assembly, and that the nucleosome remodeling deacetylase (NuRD) complex remodeling agent helps to repress gene binding to chromatin, regulating gene transcription, genome stability, and developmental signaling [142]. A study found that exposure to cigarette smoke was associated with hypermethylation of the CHD1 promoter [143]. Factors that interact with components of the transcriptional machinery and histone modifiers converge upstream of CHD1 to regulate its expression. For example, the Pol II‐associated factor hPAF2/PD2 mediates MLL‐mediated deposition of H3K4me2/3 covalent modifications characteristic of transcriptionally active genes and promotes CHD1 expression in pancreatic cancer cells [144]. In addition, CHD enzymes act downstream of key signaling pathways that are disrupted during tumorigenesis, and the chromatin remodeling activity of CHD enzymes appears to be critical for translating information from ligand‐mediated signaling pathways into the transcriptional machinery [145, 146, 147]. It is easy to see that CHD controls fundamental processes, including transcription, proliferation, and DNA damage repair, by controlling access to DNA by the cellular machinery.

##### 2.2.4.4 INO80 Complexes

The INO80 complex is an evolutionarily conserved ATP‐dependent chromatin remodeling complex, and like SWI/SNF, INO80 can be divided into ATPase, ARP, and body modules. INO80 is the major ATPase subunit of the INO80 complex and has a wide range of effects on a variety of cellular processes, including transcriptional regulation, DNA replication and repair, telomere maintenance, and chromosome segregation [148, 149]. In yeast, INO80 has also been implicated in the removal and degradation of ubiquitinated RNAPII from chromatin [150]. Gowans demonstrated that the INO80 complex mediates metabolic signaling in chromatin, remodeling coordinated metabolic homeostasis and cell division [151]. Chakraborty and Magnuson found that INO80 promotes repression of sex‐linked gene expression during spermatogenesis in mice by regulating chromatin accessibility [152]. In cancer, the INO80 chromatin remodeling complex plays an important role in many tumors. INO80 is important for the maintenance of genomic stability, and inactivation or depletion of INO80 results in aneuploidy and chromosomal structural abnormalities [153, 154]. Since there is a causal relationship between genomic instability and tumorigenesis [155], these findings suggest that INO80 may act as a tumor suppressor [156]. Less consistent with this conventional account, however, Lee et al. found that INO80 haploinsufficiency inhibits colon cancer tumorigenesis by increasing apoptosis through activation of replication stress‐induced ATR–Chk1 signaling [156]. Belk et al. demonstrated, by in vivo clustered regularly interspaced short palindromic repeats (CRISPR) screenings in mouse and human tumor models, that the relationship between INO80 and the perturbation of the BAF chromatin remodeling complex improved T cell persistence in tumors [157]. In addition, Prendergast et al. showed that the ATP‐dependent chromatin remodeling INO80 complex promotes R‐loop resolution, and counteracting the R‐loop promotes cancer cell proliferation and avoids DNA damage‐induced death [158]. These results suggest that the INO80 complex plays a critical role in transcriptional regulation, DNA damage repair, and stem cell maintenance by affecting chromatin accessibility.

#### 2.2.5 Transcription Factors

Transcription factors are widely recognized for their ability to bind to conserved motifs (unmethylated DNA sequences) within promoters to modulate transcription [159]. There is a bidirectional regulatory relationship between transcription factors and chromatin accessibility, and this interaction constitutes a central aspect of gene expression regulation.

This is first reflected in the active regulation of chromatin accessibility by transcription factors. Transcription factors are key regulators of cellular processes, and the activity of transcription factors can be modulated either by regulating the abundance of their active forms (including transcriptional, translational, and posttranslational regulation) or by regulating the accessibility of their binding sites (including epigenetic processes and cell‐type‐specific chromatin states). Once bound, transcription factors can open chromatin for or prevent binding of other factors and activate or repress transcription of genes [160]. Pioneer transcription factors play an important role in regulating chromatin accessibility, thereby defining the epigenetic landscape of the cell. This is most evident for the so‐called pioneer TF class, whose definition is based on their ability to bind to closed chromatin [160, 161]. Brennan et al., by studying TF binding data and chromatin accessibility data in early Drosophila embryos, showed that chromatin accessibility during genome activation follows complex sequence rules and is mediated by both the pioneer TF and the transcriptional activators driven in distinct steps [162]. There is also growing evidence that nonpioneer transcription factors can regulate chromatin, and Benveniste et al. has shown through a large‐scale computational study that histone modifications can be predicted very accurately from transcription factor binding profiles, outlining known interactions between transcription factors and chromatin‐modifying enzymes [163]. A study also showed by single‐cell transcriptional and chromatin accessibility analyses that chromatin accessibility correlates with cell type‐specific transcription factor activity and chromatin interaction networks [164].

Second is the regulation of transcription factors by chromatin accessibility. Enhancers are a class of cis‐regulatory elements that are key drivers of cell‐type‐specific gene expression, and because enhancers require TF binding, they rely heavily on chromatin accessibility to trigger transcriptional activity. Chromatin accessibility is therefore an important regulator of enhancer function [165]. Klemm et al.’s observation that ∼94% of all ENCODE TF ChIP‐seq peaks belong to accessible chromatin supports this view, and that the organization of accessible chromatin throughout the genome reflects a network of permissive physical interactions in which enhancers, promoters, insulators, and chromatin‐binding factors synergistically regulate gene expression through this network [8]. Wang et al. determined that chromatin accessibility determines the potential of bile acid‐dependent transcription factors to regulate antimicrobial peptides (AMPs) at the pretranscriptional level, shaping the regional heterogeneity of AMPs between the small and large intestine [166].

As for the role of transcription factors in cancer by regulating chromatin accessibility, some studies have verified this. Liu et al. generated high‐quality single‐cell chromatin accessibility profiles of epithelial cells from 29 CRC patients and found that subtype‐specific transcription factors bind to different sets of target genes and contribute to the similarity and diversity of chromatin accessibility and RNA expression among patients. In addition, the CpG island methylator phenotype was identified and the chromatin status of the CIMP‐high subtype was characterized and TF regulators identified [167]. Chen et al. found that CD8+ T cells from individuals with cancer or chronic viral infections express high levels of Nr4a transcription factors and show enrichment of Nr4a binding motifs in accessible chromatin regions. Tumor‐infiltrating lymphocytes targeted for Nr4a1/2/3 triple knockdown display robust effector functions: reduced expression of the inhibitory receptor, increased cytokine production, and strong enrichment of accessible chromatin for motifs involved in effector function transcription factors [168]. Helminen et al. have identified, by genome‐wide techniques, several key features of the glucocorticoid receptor (GR) action on PC cells, and that in enzalutamide (ENZ)‐exposed PC cells, GR substitution for the androgen receptor (AR) occurs almost exclusively at accessible chromatin loci displaying occupancy of the pioneer factor Forkhead box A1 (FOXA1). Silencing of FOXA1 enhances the chromatin‐binding and transcriptional activity of GR, identifying chromatin accessibility and FOXA1‐mediated repression as important regulators of GR action in PC, pointing to novel pathways to counter steroid receptor‐mediated antiandrogen resistance [169]. These studies reveal molecular pathways by which transcription factors play key roles in cancer by remodeling chromatin accessibility.

#### 2.2.6 Chromatin 3D Structure

3D chromatin architecture refers to the form of spatial organization of genomic DNA in the nucleus formed by multiple levels of folding, including TADs, chromatin loops, and active (A) or repressive (B) compartments [170]. Recent studies have emphasized the importance of the 3D structure of chromatin in regulating various cellular processes, especially transcription. This is achieved through a dynamic chromatin structure that controls spatial accessibility and thus dynamically regulates gene expression. Quiroga et al. have argued that the 3D structure of chromatin plays an important role in gene regulation and cellular identity by regulating contacts between motifs and gene promoters through reattachment [171]. Kirkland et al. showed that LamC deletion may reduce chromatin accessibility of cardiomyocytes by decreasing their expression of transcription factors as well as cytoskeletal regulators [172]. A study proposes that T cell activation is associated with disruption of long‐range chromatin interactions as well as partitioning of TADs and remodeling of their TAD boundaries. Newly formed/enhanced TAD boundaries are associated with higher nucleosome occupancy and lower accessibility [173]. Chromatin loops typically connect enhancers and promoters and play a crucial role in the regulation of gene transcription [174]. Also due to dynamic long‐range preferential interactions, chromosomes segregate into two forms of mutually exclusive chromatin: compartments A and B. Compartment A corresponds to active transcription and open chromatin regions, whereas compartment B is compressed and enriched with repressive chromatin features [175, 176]. These studies serve as a demonstration of the role of the 3D structure of chromatin in influencing chromatin accessibility, which in turn determines the activation or repression of genes.

The 3D structure of chromatin plays a key role in development, gene regulation, and cellular identity. Alterations in this structure can have profound effects on cellular phenotypes and have been linked to a variety of diseases, including many types of cancer. Alterations in 3D chromatin structure through a variety of different mechanisms have been found to be associated with the development of various cancers. Mutations in adhesins, one of the most common mutations in cancer, have been shown to lead to dysregulation of DNA cycling within chromosomes, thereby affecting genome organization and gene expression [177]. Disruption of the 3D structure of chromatin in cancer often leads to activation of proto‐oncogenes or silencing of oncogenes. For example, some studies have found that chromatin is disrupted in some cancers as a result of genomic rearrangements or structural variants of this genomic structure, thereby affecting the regulatory landscape of cancer cells [178]. Sui et al. found that the MLL–AF9 fusion disrupts the 3D chromatin landscape and may contribute to dramatic transcriptome remodeling in MLLr acute myeloid leukemia (AML) by comprehensively analyzing 3D genomic structure, chromatin accessibility, and gene expression in samples of MLL–AF9 AML, a genetically edited aggressive with AML [179]. Luo et al. showed that HOTTIP‐mediated R‐loop formation directly enhances CTCF chromatin boundary activity and TAD integrity to drive oncogene transcription and leukemia progression [180]. Lai et al. found that knock‐in of the C‐terminus of hematopoietic‐specific nucleophosmin 1 reshaped the TAD topology, resulting in disruption of cell cycle regulation as well as aberrant chromatin accessibility and aberrant homologous gene expression, leading to blocked myeloid differentiation [181]. Recent in vitro investigations of PC metastasis have shown that the metastatic potential and aggressiveness of PC are also closely linked to chromatin compartmentalization and dynamic genomic alterations. During cancer progression, there is extensive genomic compartmentalization, which leads to significant changes in the nuclear chromatin activation environment, resulting in increased mixing and interactions in the A compartment [182]. In addition, in metastatic pancreatic cancer cells, there is an increase in the number of chromatin loops, along with the appearance of cell‐specific chromatin loops. LIPC is a gene that promotes pancreatic cancer metastasis and is associated with tumor cell migration and invasion. LIPC expression is regulated by enhancer 3 and enhancer 4, while tissue‐specific chromatin loops form progressively during distant metastasis of pancreatic cancer, enhancing LIPC expression [183]. These studies suggest that aberrant changes in the 3D structure of chromatin can drive tumorigenesis by remodeling chromatin accessibility and gene regulatory networks.

### 2.3 Technologies for Profiling Chromatin Accessibility and 3D Genome Architecture

The rapid advancement of chromatin accessibility research is closely linked to breakthroughs in high‐throughput sequencing technologies. From early population‐level (bulk) analyses to the current single‐cell and spatial multiomics profiling, technological evolution has not only mapped the dynamic chromatin landscape in cancer but also profoundly revealed the epigenetic basis of tumor heterogeneity, plasticity, and therapy resistance. This section will systematically review classical and cutting‐edge technologies for analyzing chromatin accessibility and 3D conformation, with a focus on their innovative applications and potential for clinical translation in cancer research.

#### 2.3.1 ATAC‐seq and DNase‐seq

Genome‐wide chromatin accessibility profiling was initially driven by DNase‐seq, which uses DNase I digestion followed by high‐throughput sequencing to map DNase I hypersensitive sites at base‐pair resolution and thereby identify active regulatory elements across the genome. DNase‐seq has been instrumental in the ENCODE project and in defining regulatory landscapes in diverse human cell types, including multiple cancer models [184, 185].

The development of ATAC‐seq, which relies on a hyperactive Tn5 transposase to simultaneously cut DNA and insert sequencing adapters, has substantially simplified chromatin accessibility profiling and reduced input requirements to as few as hundreds of cells. Compared with DNase‐seq, ATAC‐seq requires less material and handling time, provides comparable resolution for regulatory elements, and additionally preserves nucleosomal “laddering” patterns that inform nucleosome positioning and transcription‐factor occupancy [186, 187].

In cancer research, bulk ATAC‐seq and DNase‐seq have been widely used to map tumor‐specific regulatory elements and infer transcriptional regulatory networks. Large‐scale efforts have generated chromatin accessibility atlases of primary human cancers, linking tumor‐type‐specific open chromatin to oncogenic drivers and noncoding risk variants [188, 189]. Recent work is extending these assays to clinically relevant specimens: for example, optimized ATAC‐seq protocols for formalin‐fixed paraffin‐embedded (FFPE) samples and spatial FFPE–ATAC‐seq now enable in situ accessibility profiling in archived tumor tissues with preserved tissue architecture, opening a path toward retrospective clinical studies and routine translational applications [190, 191].

#### 2.3.2 Single‐Cell Chromatin Accessibility and Multiomics

Bulk assays average signals across heterogeneous cell populations and therefore obscure rare cell states and evolutionary trajectories that are central to cancer biology. Single‐cell ATAC‐seq (scATAC‐seq) overcomes this limitation by resolving chromatin accessibility profiles at single‐cell resolution, enabling the reconstruction of regulatory cell states, lineage relationships, and subclonal architectures within tumors and their microenvironment [192]. Recent pan‐cancer studies have generated large‐scale scATAC‐seq atlases from primary or archival tumor samples, revealing tumor‐type‐specific regulatory programs, immune‐cell state transitions, and the impact of copy‐number alterations on chromatin landscapes [193, 194]. Beyond accessibility alone, single‐cell multiomics technologies jointly measure chromatin accessibility, transcriptomes, and in some cases additional layers such as DNA methylation or protein abundance in the same cell. Early coassays such as sci‐CAR and SHARE‐seq established that simultaneous profiling of ATAC and RNA can directly link distal regulatory elements to their target genes and predict “chromatin potential” of future transcriptional states [195, 196]. Newer platforms such as Parallel‐seq and related methods have further increased throughput and robustness, facilitating the application of single‐cell multiomics to clinically annotated tumor cohorts and therapy‐response studies [197].

In cancer immunology, single‐cell multiomics is beginning to resolve how chromatin accessibility and transcriptional programs jointly shape T‐cell exhaustion, myeloid cell reprogramming, and neoantigen‐driven immune responses, thereby directly informing epigenetic‐based combination immunotherapies and minimal residual disease monitoring [198, 199]. From a mechanistic standpoint, these approaches provide a unique opportunity to causally link chromatin accessibility dynamics to changes in cell state plasticity, stemness, and therapy resistance.

#### 2.3.3 3D Chromatin‐Conformation Assays

Chromatin accessibility is tightly coupled to higher‐order 3D genome architecture. Chromosome‐conformation capture‐based methods, particularly Hi‐C and in situ Hi‐C, quantify physical contacts between distal genomic loci, enabling the discovery of TADs, chromatin loops, and long‐range enhancer–promoter interactions [200, 201]. In cancer, 3D genome reorganization can disrupt TAD boundaries, create oncogenic enhancer hijacking events, and rewire regulatory hubs, thereby reshaping chromatin accessibility and gene expression programs that drive tumorigenesis and progression [202, 203].

Newer 3D genome assays, including Micro‐C, HiChIP, and PLAC‐seq, increase resolution or enrich for specific protein‐anchored chromatin interactions, allowing finer mapping of regulatory loops around oncogenes and tumor suppressors [204, 205, 206]. When combined with ATAC‐seq and histone‐mark ChIP‐seq, these methods delineate “3D regulatory hubs” in which clusters of accessible enhancers physically converge on key transcription factors and lineage‐defining genes, providing a structural explanation for SE‐driven transcriptional addiction and for context‐specific vulnerabilities that may be exploited therapeutically [207, 208].

Importantly, 3D chromatin‐conformation assays are beginning to be applied to patient‐derived organoids (PDOs), ex vivo coculture models, and longitudinal biopsy samples, offering a route to study how therapy reshapes genome topology and how emergent 3D configurations correlate with minimal residual disease or acquired resistance [209, 210, 211]. Integrating these 3D maps with the chromatin accessibility and multiomics datasets discussed above will be critical for moving from correlative associations toward mechanistic models of genome folding in cancer.

Collectively, these complementary technologies—bulk ATAC‐seq and DNase‐seq, single‐cell multiomics, and 3D chromatin‐conformation assays—provide a multiscale view of chromatin accessibility in cancer, ranging from nucleotide‐level regulatory elements to cell‐type‐specific programs and higher‐order genome topology. Their joint application in clinically annotated cohorts will be essential for identifying robust accessibility‐based biomarkers, for disentangling tumor‐intrinsic versus microenvironment‐driven regulatory changes.

## 3 Role of Chromatin Accessibility in Tumors

**FIGURE 2:**
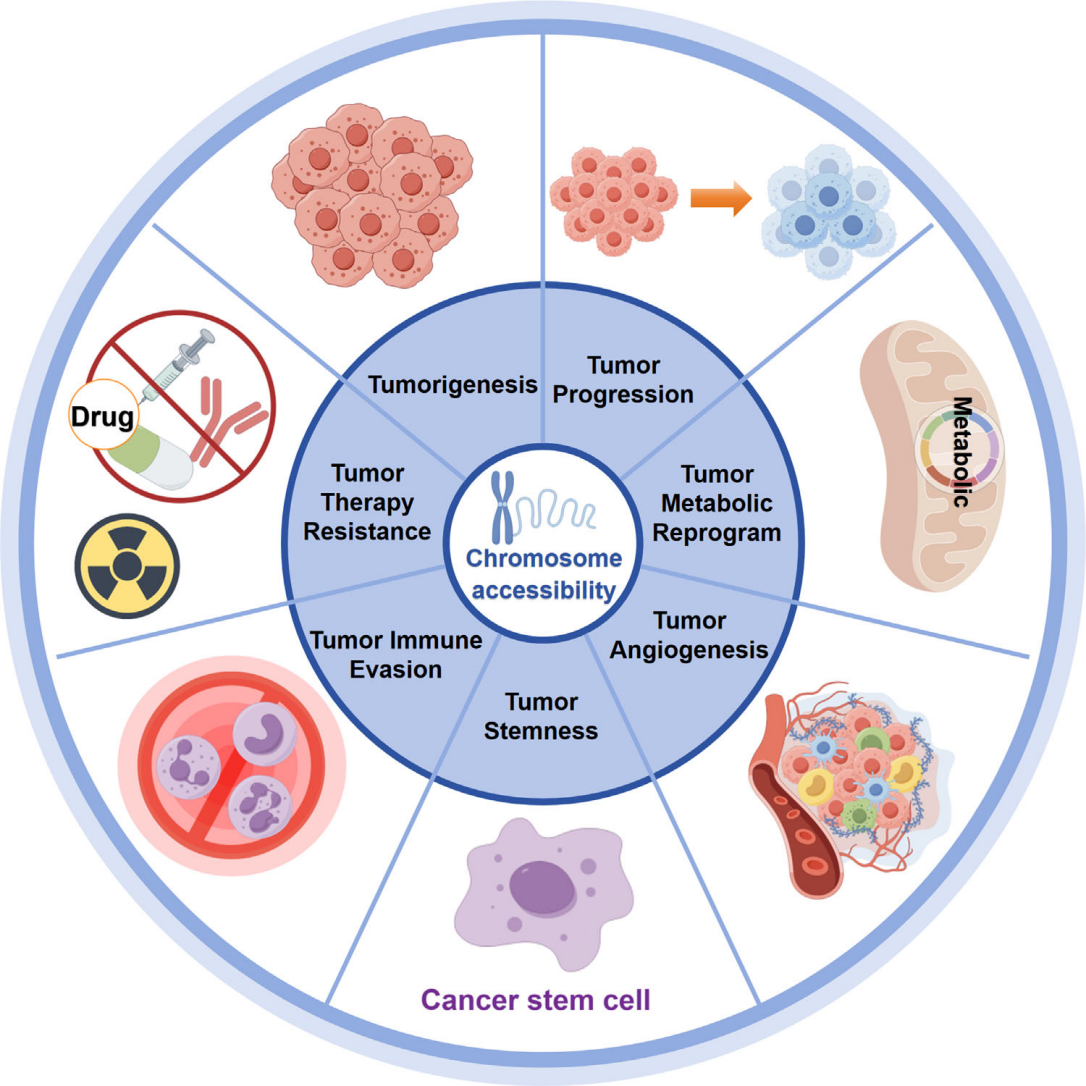
The role of chromatin accessibility in tumors. The role of chromatin accessibility in tumors, including: tumorigenesis, tumor progression, tumor metabolic reprogramming, tumor angiogenesis, tumor stemness, tumor immune evasion and microenvironment, and tumor therapy resistance.

**FIGURE 3:**
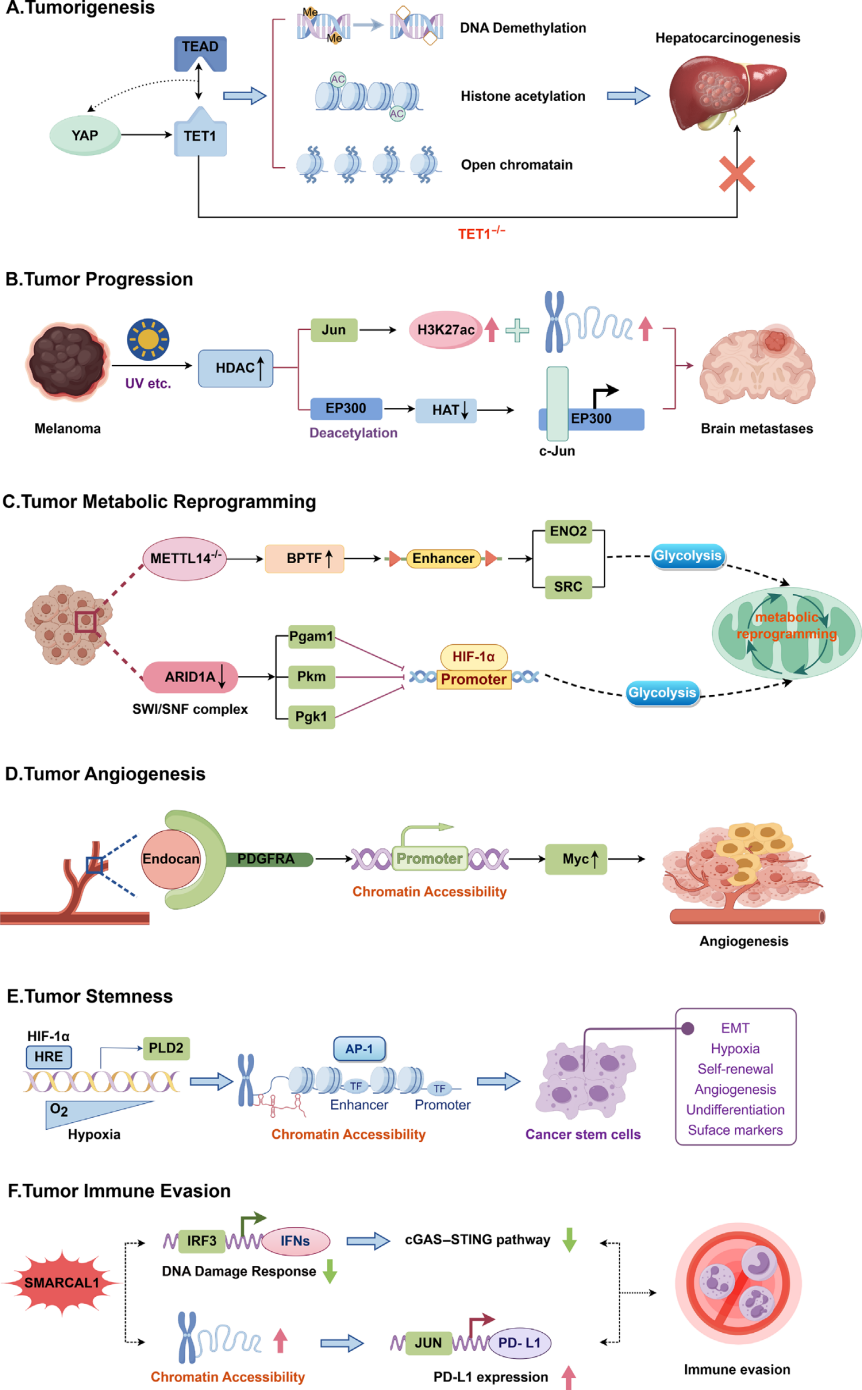
Schematic representation of the mechanisms underlying the role of chromatin accessibility in tumors. (A) YAP–TEAD complex interacts with TET1 to demethylate DNA at YAP target genes in the liver. Loss of TET1 reverses YAP‐induced chromatin and transcriptional changes and suppresses YAP‐induced hepatomegaly and tumorigenesis. (B) Stress‐induced (e.g., UV irradiation) HDAC8 activity regulates an invasive melanoma cell state, deacetylates EP300, inhibiting its enzymatic activity, switching its association from MITF sites to Jun sites and increases H3K27 acetylation and chromatin accessibility at Jun‐target gene transcriptional sites, thereby increasing the development of melanoma brain metastases. (C) BPTF constituted super‐enhancers that activate downstream targets like enolase 2 and SRC proto‐oncogene nonreceptor tyrosine kinase, leading to glycolytic reprogramming of METTL14−/− cells; ARID1A loss increased chromatin accessibility and enhanced HIF‐1α binding to the promoter regions of Pgam1, Pkm, and Pgk1, similarly promotes glycolytic reprogramming. (D) Endocan, a proteoglycan secreted by endothelial cells, can directly bind to the PDGFRA receptor and activate its activity, thereby enhancing the chromatin accessibility of the Myc promoter and upregulating Myc expression, which in turn confers enhanced proliferation, migration, and angiogenesis capabilities to GBM cells. (E) HIF‐1α activates PLD2 transcription through hypoxia response elements, increased accessibility of AP‐1 transcription factor binding sites induces cancer stem cells formation. (F) The DNA translocase SMARCAL1 favors tumor immune evasion by two distinct mechanisms: it suppresses the cGAS–STING pathway by limiting endogenous DNA damage and induces PD‐L1 expression by modulating chromatin accessibility at a PD‐L1 regulatory element.

We explore the multidimensional role of chromatin accessibility in tumors, including tumorigenesis, progression, metabolic reprogramming, angiogenesis, tumor stemness, immunity and microenvironment, and tumor therapy resistance (Figures 2 and 3).

### 3.1 Tumorigenesis

Tumorigenesis is a complex, gradual process primarily driven by the activation of oncogenes and the inactivation of TSGs [212]. In tumor cells, alterations in chromatin accessibility play a crucial role in tumorigenesis and progression.

Chromatin accessibility regulates proto‐oncogenes and oncogenes by modulating promoter and enhancer activity [213]. For example, protein arginine methyltransferase (PRMT) 1 promotes PDAC by increasing chromatin accessibility at glycolytic gene promoters and enhancers [214]. Similarly, SE RNA m6A modification enhances chromatin accessibility, driving PDAC‐associated gene transcription, ultimately driving PDAC development [107]. SET domain containing 2 (SETD2), a frequently mutated chromatin modifier in cancer, facilitates KRAS‐driven lung tumorigenesis by increasing enhancer accessibility and upregulating polycomb repressive complex 2 (PRC2) and KRAS signaling genes [215].

Epigenetic mechanisms—including DNA methylation, histone modifications, transcription factors, and ncRNAs—further influence tumorigenesis by altering chromatin structure. The YAP–TEAD complex upregulates TET1, which promotes DNA demethylation, H3K27ac modification, and chromatin opening at YAP target genes, ultimately accelerating liver tumorigenesis [216]. In addition, recent studies have found that lactate‐induced lactic acidification further affects lactate production, recycling, and utilization, which in turn promotes tumorigenesis [217]. Lactate, through histone lactylation, modifies chromatin structure to regulate gene expression, linking metabolic reprogramming to tumor progression [218]. In melanoma, PHB2 recruits MLL2 to the CANT2 promoter, enhancing H3K4me3 and activating this oncogenic lncRNA while suppressing the tumor suppressor CCBE1 [219].

In addition, the role of chromatin remodeling complexes in tumorigenesis cannot be ignored. SMARCA5, an ISWI complex ATPase, maintains aberrant chromatin accessibility in leukemia by recruiting DDX5 and SP1 to activate AKR1B1, which reprograms fructose metabolism and worsens patient outcomes [220]. CARM1 promotes the development of breast cancer by methylating the NuRD complex to upregulate cell cycle genes [221]. Similarly, Wei et al. found that a JmjC family protein, JARID2, is highly expressed in several types of cancers including breast cancer, and JARID2 promotes breast tumorigenesis by interacting with the NuRD complex, especially playing a key role in the adipocyte‐derived leptin response [222].

The impact of chromatin accessibility extends beyond these examples. Studies have highlighted its role in the progression of various cancers, including CRC [223], gastric cancer [224], PC [225], and ovarian cancer (OC) [226], underscoring the multifaceted contribution of chromatin remodeling in tumorigenesis.

### 3.2 Tumor Progression

Chromatin accessibility, as a core hub of epigenetic regulation, plays a key role in tumor proliferation, invasion, and metastasis, and recent studies have revealed that it can contribute to the molecular panorama of tumor malignant evolution through multidimensional mechanisms.

Dysregulated chromatin accessibility fuels tumor proliferation by activating oncogenic pathways. For instance, HMGA1 drives myeloproliferative tumor progression by enhancing chromatin accessibility at the GATA2 locus, recruiting activating histone marks to stimulate proproliferative gene networks [227]. Similarly, BRD8 sustains GBM proliferation via the EP400 complex, which deposits the histone variant H2AZ at p53 target loci to repress cell cycle arrest signals [228]. In neuroblastoma, activator protein‐1 (AP‐1) remodels chromatin to expose IRF2BP2 binding sites, activating ALK signaling through SEs to drive proliferation [229]. Microtubule‐associated serine/threonine kinase‐like (MASTL) has a key function in mitotic regulation, and it has been shown that IL‐6 and TNF‐α stimulation induces trimethylation of H3K4Me3 at the MASTL promoter to promote chromatin accessibility, thereby promoting HCC proliferation [230].

Chromatin accessibility dysregulation facilitates tumor invasion by rewiring oncogenic signaling pathways. For example, in early‐stage LUAD, SE activation at the LINC01977 locus drives malignancy by hijacking the TGF‐β/SMAD3 pathway, linking chromatin remodeling to invasive progression [231]. Similarly, in melanoma, imprinted site regulator BORIS, also known as CCCTC binding factor, which mediates altered chromatin accessibility promotes a proinvasive transcriptional signature [232]. In PDAC, chromatin accessibility profiling identified KRAS‐activated FOSL2 as a critical mediator of invasion. FOSL2 upregulates CCL28 to promote tumor cell migration and invasion, correlating with poor patient prognosis [233]. Zheng et al. showed that HBV X protein (HBx) is the most frequently integrated viral gene sequence after HBV infection. HBx increases chromatin accessibility and ETV4 expression to regulate dishevelled‐2 and promote HCC cell migration and invasion, and high expression of ETV4 is correlated with poor prognosis of HCC patients [234].

Dynamic chromatin accessibility changes are central to epigenetic reprogramming during metastasis, enabling transcriptional plasticity that drives tumor dissemination. For example, histone deacetylase (HDAC) 8 upregulation in melanoma increases H3K27ac levels and enhances c‐Jun binding site accessibility, promoting invasive phenotypes and brain metastasis [235]. Similarly, the lncRNA MALAT1 fuels LUAD metastasis by amplifying chromatin accessibility at inflammatory loci, upregulating CCL2 to remodel the tumor microenvironment [105]. Inactivation of tumor suppressors further reshapes metastatic potential. LKB1 loss in LUAD alters chromatin landscapes to accelerate metastatic progression [236]. In HCC, the transposon‐derived lncMER52A—dependent on chromatin accessibility for its expression—promotes metastasis by interacting with p120‐catenin [237]. Epigenetic regulators like Cat eye syndrome chromosome region, candidate 2 (CECR2) also play key roles: in breast cancer, CECR2 increases chromatin accessibility to activate NF‐κB signaling, fostering immunosuppressive microenvironments and metastasis [238]. SETD2 mutations exemplify another mechanism, where loss of H3K36me3 activates enhancers that drive prometastatic transcription, creating dependencies on histone chaperone complexes [239]. These mechanisms extend across cancers, including colorectal [240], osteosarcoma [241], pancreatic [242], bladder [243], and prostate malignancies [244]. These results suggest that dynamic alterations in chromatin accessibility in tumor cells can drive aberrant tumor proliferation, invasion, and metastasis by remodeling the transcriptional program and regulating the activation of oncogenes and metastasis‐related pathways. Future studies need to combine multiomics data to resolve the driving mechanisms of accessibility changes in specific tumor types and develop precise therapeutic strategies targeting open regions of chromatin.

### 3.3 Regulation of Metabolic Reprogramming

Metabolic reprogramming—a hallmark of cancer—enables tumor cells to adapt to nutrient demands through enhanced glycolysis (the Warburg effect), dysregulated glutamine metabolism, and increased lipid synthesis [245, 246]. Emerging research highlights chromatin accessibility as a central regulator of these metabolic shifts.

Tumor microenvironment metabolites reciprocally remodel chromatin landscapes. Lactate, for instance, induces histone lactylation, altering chromatin spatial configuration to regulate DNA accessibility and gene expression. Spatial lactate gradients link metabolic activity to epigenetic rewiring, positioning lactate as a dual driver of tumor energetics and malignant progression [218]. Similarly, in NEPC, Zeb1 promotes metabolic plasticity by modulating glycolytic enzymes and reshaping chromatin accessibility to facilitate lineage plasticity [86].

Meanwhile, key transcription factors regulate metabolic enzyme expression through chromatin accessibility. For example, in CRC, the Lyn/RUVBL1 complex enhances FOXA1 chromatin accessibility, activating arachidonic acid (AA) metabolism to accelerate liver metastasis [247]. In renal cell carcinoma (RCC), METTL14 loss stabilizes BPTF mRNA, amplifying glycolysis‐associated SEs and fostering prometastatic epigenetic memory [248].

Chromatin remodeling complexes also directly regulate metabolic enzyme expression. For example, ARID1A deficiency disrupts SWI/SNF complex function, opening chromatin regions to activate glycolytic genes and sensitizing lung tumors to glutaminase inhibitors—a “synthetic lethal” vulnerability with therapeutic potential [249].

Collectively, chromatin accessibility dynamics orchestrate metabolic reprogramming by modulating enzymes, signaling pathways, and epigenetic crosstalk. These insights underscore the need to target chromatin–metabolism interplay for novel cancer therapies.

### 3.4 Tumor Angiogenesis

Tumor angiogenesis, a process by which tumor cells acquire oxygen and nutrients by inducing neovascularization, is critical for tumor growth, invasion and metastasis [250]. A growing body of evidence identifies chromatin accessibility as a critical regulator of this process via diverse epigenetic mechanisms.

Chromatin remodeling by transcriptional complexes activates proangiogenic gene networks. For example, in breast cancer, SIN3A‐associated protein, 30 kDa (SAP30), a subunit of the SIN3 corepressor complex, paradoxically enhances chromatin accessibility at promoters of genes linked to angiogenesis, lymphangiogenesis, and metastasis, driving tumor progression [251]. Similarly, in HCC, nuclear actin dynamics increase chromatin accessibility to coordinate extracellular matrix remodeling and angiogenesis‐related gene expression, promoting metastasis [252]. Resistance to antiangiogenic therapies is closely associated with dynamic changes in chromatin accessibility. For instance, amilotinib‐resistant tumor cells exhibit increased accessibility at angiogenesis‐associated genomic regions, with TFAP2A driving resistance via proangiogenic transcriptional programs [253]. In GBM, endothelial‐secreted Endocan activates PDGFRA signaling, enhancing MYC promoter accessibility to stabilize tumor‐promoting phenotypes and underscoring vascular–tumor crosstalk in disease progression [254]. In addition, angiogenesis is epigenetically linked to metabolic reprogramming. For example, Li et al. found that EZH2‐mediated H3K27me3 deposition suppresses transsulfuration pathway genes, but inhibition of EZH2 (e.g., Tazemetostat) restores chromatin accessibility, reactivating cysteine–methionine metabolism and lipid homeostasis while suppressing angiogenesis [255].

These results suggest that altered chromatin accessibility plays an important role in tumor angiogenesis by modulating the expression of proangiogenic factors and endothelial cell function. Future studies should further resolve the heterogeneity of angiogenesis‐associated chromatin opening patterns in different tumor types and develop epigenetic drugs targeting specific accessibility regions to optimize strategies for antiangiogenic therapy.

### 3.5 Tumor Stemness

Tumor stemness refers to the key characteristics of tumor cells with self‐renewal, multidirectional differentiation, and tumorigenic ability, which are closely related to tumorigenesis, metastasis, and recurrence [256]. Recent studies have shown that dynamic remodeling of chromatin accessibility plays a central role in maintaining the self‐renewal and differentiation of CSCs by regulating the spatiotemporal expression patterns of stemness‐related genes.

Metabolic–epigenetic crosstalk modulates CSC properties. In leukemia, nuclear hexokinase 2 (HK2) enhances chromatin accessibility at DNA repair and stemness loci, reinforcing leukemic stem cell identity [257]. Furthermore, hypoxia drives CSC's characteristics through epigenetic reprogramming. For example, Hypoxia further reprograms CSCs via HIF‐1α, which activates AP‐1 binding sites and increases PLD2 transcription to drive chemoresistance in OC [258]. Similarly, abnormalities in the SWI/SNF chromatin complex affect stemness homeostasis, and ARID1A is a key subunit of the SCRC, and Wang et al. found that ARID1A deficiency increased the expression of hepatic stem/progenitor cell markers and enhanced cellular self‐renewal, as well as remodeled chromatin accessibility of genes associated with liver function. Thus, ARID1A deficiency may increase the number of stem/progenitor‐like cells by dysregulating the expression of these genes associated with cell stemness, differentiation, and liver function, leading to cancer development [259]. Similarly, MUC1‐C promotes prostate CSC self‐renewal by activating the PBAF complex to enhance chromatin accessibility, linking redox homeostasis to stemness [260]. Targeting MUC1‐C inhibits NEPC CSC tumorigenicity and therapy resistance [261].

ncRNAs and histone variants fine‐tune chromatin landscapes. In HCC, CircHULC drives CSC growth by inducing chromatin reprogramming and genomic instability [262]. Nikolic et al. revealed that the histone variant macroH2A2 shapes chromatin accessibility at enhancer elements to antagonize the epigenetic program of stemness in GBM [263].

Based on the above studies, it is easy to see that chromatin accessibility alterations maintain the self‐renewal and differentiation potential of CSCs by regulating stemness‐associated transcription factors and epigenetic modifications. Future studies should further resolve the heterogeneity of chromatin opening patterns in different tumor types and develop precise therapeutic strategies targeting the specific accessibility regions of CSCs to overcome tumor resistance and recurrence.

### 3.6 Tumor Immunity and Microenvironment

Tumor immune evasion and the immunosuppressive tumor microenvironment are critical barriers to effective cancer therapy [264]. Chromatin accessibility plays a pivotal role in this process by regulating immune checkpoint molecules and immune cell functionality.

Chromatin remodeling drives immune evasion by upregulating immunosuppressive signals [265]. For example, SMARCAL1, a DNA translocase, collaborates with the transcription factor JUN to maintain chromatin accessibility at the PD‐L1 promoter, enhancing its expression and enabling tumor cells to escape immune detection [266]. Similarly, the transcription factor FLI1 amplifies IDO1 expression by coordinating CBP and STAT1 activity, promoting IFN‐γ‐induced kynurenine production and suppressing T cell responses [267].

Tumor‐infiltrating regulatory T cells exhibit distinct chromatin accessibility patterns compared with peripheral blood Tregs, with enriched AP‐1 motifs in enhancers linked to immune suppression and leukocyte differentiation [268]. Combining DNMT and EZH2 inhibitors reduces promoter methylation, increases chromatin accessibility, and reactivates interferon‐stimulated genes, restoring antitumor immunity in HCC [269]. In NSCLC, IL‐4 suppresses TAP2 expression via chromatin remodeling, impairing antigen presentation and promoting immune evasion [270].

Chromatin accessibility regulates extracellular matrix (ECM) remodeling and metabolic pathways to shape TIME. In GBM, RUNX1 interacts with NPM1 to maintain chromatin openness at ECM‐associated genes, fostering an immunosuppressive niche [271]. WDR6 promotes HCC progression by enhancing TNF‐α expression through chromatin remodeling, while MPC activity preserves cytotoxic T cell function by maintaining histone acetylation and promemory gene accessibility [68, 272].

In addition, Loss of tumor suppressors (NF1, TSC1, TGF‐β RII) alters chromatin landscapes, activating the IL6‐JAK3‐STAT3/6 pathway and recruiting immunosuppressive LAG3+ T cells [273]. In nasopharyngeal carcinoma, EBV infection reduces CTCF levels, driving CD74‐mediated T cell exhaustion and epigenetic reprogramming [274]. Moreover, SETD2 inactivation impairs H3K36me3 deposition, reducing NR2F1 transcription and activating STAT1 to enhance PD‐1 expression, thereby reshaping TIME [275].

These findings suggest that altered chromatin accessibility plays a critical role in tumor immune evasion and immune microenvironment remodeling through multilevel regulation of immune‐related gene expression. The integrated multiomics analysis and the establishment of organoid models will help to deeply understand the regulatory mechanism of chromatin accessibility in the dynamic changes of TIME and provide new ideas for the development of novel immunotherapy strategies.

### 3.7 Tumor Therapy Resistance

Tumor therapeutic resistance is a major challenge in clinical cancer treatment, including various forms of chemotherapy resistance, targeted therapy resistance, endocrine therapy resistance, immunotherapy resistance and radiotherapy resistance. Recent studies have found that chromatin accessibility plays a central role in multiple drug resistance mechanisms by dynamically regulating key pathways such as drug metabolism, target expression, DNA damage repair, and immune microenvironment [18, 218, 276].

#### 3.7.1 Chemotherapy Resistance

Chemoresistance is a major challenge in tumor therapy. Y‐box binding protein 1 (YBX1) stabilizes CHD3 mRNA via m5C modification recognition, enhancing chromatin accessibility to promote homologous recombination and platinum resistance in OC [277]. Similarly, the histone demethylase KDM4 increases promoter accessibility of senescence‐associated genes, fostering chemoresistance in PC, while KDM4 inhibition reverses this phenotype [278]. In leukemia, nuclear HK2 elevates chromatin accessibility at DNA repair and stemness loci, reducing double‐strand breaks and conferring chemoresistance in leukemia stem cells [257]. CBX2 drives CRC chemoresistance by maintaining chromatin accessibility via the RUNX1–CBX2–MAP4K1–pERK axis, with CBX2 knockdown inducing epigenetic reprogramming and sensitizing tumors to therapy [223]. In squamous cell carcinoma, DLGAP1–AS2 enhances H3K27ac‐marked chromatin accessibility at the FAM3D locus, activating YAP signaling through phosphatidic acid synthesis to promote chemoresistance [279]. Hypoxia induces chemoresistance in OC by stabilizing HIF‐1α, which increases AP‐1 binding site accessibility and activates PLD2 transcription. PLD2 overexpression mimics hypoxia to enhance cisplatin and carboplatin resistance [258]. Overall, dynamic changes in chromatin accessibility can dynamically orchestrate the stress response and survival adaptation of cancer cells to chemotherapeutic agents by modulating DNA repair capacity, remodeling epigenetic status, and activating key signaling pathways, thereby driving the onset and development of chemoresistance in a wide range of solid and hematologic tumors.

#### 3.7.2 Targeted Therapy Resistance

Recent studies have reported the role of epigenetic changes, particularly chromatin accessibility, in resistance to targeted tumor therapies. In EGFR‐mutant lung cancer, the SCRC promotes osimertinib resistance by altering chromatin landscapes, facilitating survival despite tyrosine kinase inhibition [280]. Similarly, SMARCA4 inactivation in small‐cell lung cancer (SCLC) increases chromatin accessibility at neuroendocrine (NE) transcription factor loci, driving ERBB pathway activation and sensitizing tumors to afatinib [253]. In anrotinib‐resistant lung cancer, chromatin accessibility profiling revealed enrichment of angiogenesis‐related pathways. TFAP2A accelerates resistance by activating proangiogenic programs, highlighting the interplay between chromatin remodeling and vascular signaling [281]. In addition, in OC, CYP1B1, a member of the cytochrome P450 family of enzymes, promotes PARP inhibitor resistance through histone H1.4 interactions and increased chromatin accessibility [282].

In line with the above, the aberrant expression and adaptive signaling activation of targeted therapy‐related genes are coordinated through dynamic mechanisms such as remodeling chromatin structure, modulating transcription factor activity, enhancing DNA repair capacity, and mediating tumor cell state plasticity, thereby driving the onset and evolution of targeted therapy resistance in a wide range of lung, myeloma, ovarian, and NE tumors.

#### 3.7.3 Endocrine Therapy Resistance

Endocrine therapy resistance is also a common problem in tumor therapy, and the role of chromatin accessibility in its mechanism is gradually being revealed. In a study on endocrine therapy resistance in breast cancer, YAP was found to bind to TEAD to increase local chromatin accessibility to stimulate transcription of nearby genes, and transcriptional repression of ER by YAP was demonstrated revealing the Hippo pathway as a therapeutic target in ER+ breast cancer [283]. Blawski et al. indicated that the pioneer factor FOXA1‐driven another chromatin‐accessible state in invasive lobular carcinoma (ILC) of the breast, FOXA1 regulates its own expression in a feed‐forward mechanism by binding to the unique FOXA1 enhancer site in ILC. This results in the FOXA1–ER axis promoting transcription of genes associated with tumor progression and tamoxifen resistance [284]. Chen et al. showed that AR‐indifference is a mechanism of resistance to hormone therapy in PC, in which one cut homeobox 2 (ONECUT2) regulates gene expression through promoter binding, enhanced genome‐wide chromatin accessibility, and SE reprogramming, which activates resistance with multiple drivers associated with adenocarcinoma, stem cell‐like, and NE variants [285]. The use of AR inhibitors in PC increases the plasticity of the cell line, leading to resistance to AR‐targeted therapies. In a similar study to Chen, Leppanen et al. examined the chromatin landscape of AR‐positive PC cells after exposure to the AR inhibitor ENZ, identified a novel regulator of cellular plasticity, the homology‐frame transcription factor Sine oculis homeobox homolog 2 (SIX2), whose motifs are enriched in accessible chromatin regions after treatment, and suggested that depletion of IX2 might be a possible strategy for overcoming the androgen‐resistant cellular plasticity of PC mechanism [286].

Overall, it appears that aberrant activation of hormone receptor‐dependent and nondependent pathways is coordinated through dynamic mechanisms such as activation of transcription factor complexes, remodeling of pioneer factor‐mediated chromatin states, driving SE reprogramming, and inhibition of key receptor signaling, which can affect endocrine therapy resistance.

#### 3.7.4 Immunotherapy Resistance

Immunotherapy resistance is driven by chromatin accessibility changes that suppress antigen presentation, immune checkpoint activity, and antitumor immune responses. In multiple myeloma (MM), gamma‐secretase inhibitors induce antigen shedding by altering chromatin accessibility, reducing surface antigen expression and enabling immune evasion [287]. Similarly, acute lymphoblastic leukemia cells resistant to immunotherapy exhibit reduced CD19 and CD22 promoter accessibility, downregulating antigen expression while increasing dependence on BTK signaling [288]. Conversely, HDAC inhibitors like CXD101 restore antitumor immunity in checkpoint blockade‐resistant models by enhancing chromatin accessibility and H3K27 hyperacetylation at IFNγ‐responsive genes, synergizing with immune checkpoint inhibitors (ICIs) to activate STAT1‐driven immunity [289]. In NSCLC, IL‐4 signaling in NSCLC reduces TAP2 promoter accessibility, impairing antigen processing and promoting reversible immune evasion [270].

These results suggest that reduced accessibility of drug resistance‐related gene promoters leads to downregulation of their expression, and chromatin accessibility dynamically coordinates tumor cell regulation of immune checkpoints, silencing of antigenic expression, and adaptive remodeling of immune responses, thereby driving the onset of immunotherapeutic drug resistance and adaptive immune escape in a variety of malignancies, including MM, acute lymphoblastic leukemia, and NSCLC.

#### 3.7.5 Radiotherapy Resistance

Chromatin accessibility alterations significantly influence radiotherapy resistance through diverse mechanisms, as evidenced by emerging studies. For example, Yang et al. that exosomal DEK binds directly to chromatin, increasing genome‐wide accessibility. This process triggers the quiescent state of breast cancer CSCs, thereby reducing their sensitivity to chemo‐ and radiotherapy [290]. Similarly, Dawn et al. identified a clinical correlation between the cancer/testis antigen GAGE and radiotherapy resistance in cervical cancer. Mechanistically, GAGE mediates radioresistance by modulating chromatin accessibility, suggesting its role as a biomarker for treatment failure [291]. In contrast, Maja et al. found a higher ratio of alpha radiation‐induced DNA damage in the invasive breast cancer cell line MDA‐MB‐231 cells, which may be explained by the basal heterochromatin marker higher levels and suggests that dense chromatin is associated with poor tumor prognosis and resistance to photon radiotherapy [292].

These findings suggest that altered chromatin accessibility plays a key role in tumor therapy resistance through multidimensional regulation of gene expression programs. An in‐depth understanding of the regulatory mechanisms of chromatin accessibility in drug resistance will provide an important theoretical basis for the development of novel anticancer strategies.

## 4 Chromatin Accessibility in Tumor Treatment Strategies and Clinical Trials

In recent years, with the development of high‐throughput sequencing technologies, such as ATAC‐seq and DNase‐seq, the role of chromatin accessibility in tumor therapy has been continuously explored. This section will focus on the following points.

### 4.1 Chromatin Accessibility as a Tumor Marker

#### 4.1.1 Diagnostic Markers

Chromatin accessibility dynamics reflect epigenetic reprogramming events early in tumorigenesis. Abnormalities in open chromatin regions emerge earlier than those detected by traditional mutation analysis and can be noninvasively identified via liquid biopsies, such as circulating free DNA (cfDNA) [293, 294].

A recent multicohort study found that coverage of cfDNA in promoter regions can be used as an indicator of chromatin status and reflect gene expression levels in living cells. A comprehensive survey of plasma cfDNA from 546 individuals concluded that inferred chromatin accessibility alterations derived from cfDNA profiles have the potential to be used not only for cancer screening, but also for diagnostic and preoperative evaluation [295]. A simplified cfDNA methylation assay targeting OTOP2 and KCNA3 enables accurate diagnosis of esophageal cancer, addressing the unmet need for reliable blood‐based biomarkers [296]. In Taklifi et al.’s review, it was also shown that chromatin accessibility differences are reflected in the fragmentation patterns of free DNA, and a new pipeline that integrates chromatin accessibility status into the design of liquid biopsy‐targeted sequencing panels is described for identifying labeled regions of free DNA for cancer detection, as well as cancer‐specific markers that have potential use in liquid biopsy testing [297].

In addition, the role of differential expression of chromatin accessibility in distinguishing tumors from paraneoplastic or normal tissues, as well as in the classification of cancer subtypes, has been confirmed by a number of studies. Liu et al. comprehensively investigated the genome‐wide DNAme landscapes of breast cancer and 10 other cancers and their neighboring normal and healthy breast tissues, obtained eight CpGs with large differences in chromatin accessibility status, and constructed a logistic regression model, by which it was possible to differentiate between breast cancers and normal samples as well as other cancers with high sensitivity and specificity [298]. Similarly, Peter et al. analyzed whole‐genome sequencing data from more than 1000 cfDNA samples from cancer patients and healthy controls using a self‐developed bioinformatics pipeline that inferred accessibility of transcription factor binding sites from cfDNA fragmentation patterns and showed that inferring transcription factor binding from cfDNA can help predict and early detect tumor subtypes [299]. In a similar vein, Doebley et al. developed Griffin, a framework for analyzing cfDNA nucleosome conservation and accessibility, which employs a GC correction procedure tailored to variable cfDNA fragment sizes, allowing accurate analysis of chromatin accessibility of cfDNA for cancer subtype prediction and potentially direct personalization of therapies to improve patient prognosis [300].

Chromatin accessibility markers exhibit high tissue specificity and early detection potential. Integrating these with multiomics data could establish an “epigenetic liquid biopsy” platform, revolutionizing early cancer screening, diagnosis, and subtype stratification.

#### 4.1.2 Prognostic Markers

Chromatin accessibility alterations are closely associated with tumor progression, metastasis, and clinical outcomes, offering significant potential as prognostic biomarkers.

Dynamic chromatin accessibility changes serve as real‐time indicators of tumor aggressiveness. In clear cell renal carcinoma, BAP1 mutations reduce chromatin accessibility, while PBRM1 mutations increase it, with copper–cyanophorin promoting tumor–stromal interactions to drive progression [301]. In early‐stage TNBC, Meng et al. found that knockdown of ANCO1 resulted in enhanced aneuploidy, cellular senescence, and invasion in 3D stroma, suggesting that deletion of ANCO1 expression regulates chromatin accessibility and promotes progression of early‐stage TNBC [302]. Xu et al. identified a new cell subpopulation with abnormally high CXCL14 expression levels in positive lymph nodes of breast cancer patients by a combination of scRNA‐seq and scATAC‐seq assays to obtain chromatin accessibility profiles, suggesting that CXCL14 is a key regulator and marker of lymph node metastasis of breast cancer [303]. LaFave et al. proposed that epigenomic state transitions are characteristic of LUAD progression in mice, and they used single‐cell epigenomics to analyze chromatin state transitions in a LUAD mouse model characterized by activation of the RUNX transcription factor, which mediates extracellular matrix remodeling to promote metastasis [304]. These results suggest that dynamic changes in chromatin accessibility can serve as a real‐time monitoring indicator and biomarker of tumor aggressiveness.

In addition, in terms of predicting tumor prognosis and survival, some studies in recent years have also confirmed the role played by chromatin accessibility. In PDAC, transcription factors ZKSCAN1 and HNF1B exhibit differential chromatin accessibility patterns predictive of disease‐free survival [305]. NSCLC patients with ARID1B mutations show improved survival due to enhanced DNA damage response and cGAS–STING pathway activation, highlighting its prognostic utility [306]. In GBM, elevated chromatin accessibility at the GSTM1 locus predicts shorter survival, validated across multiple patient cohorts [307]. Through these studies, we can see that chromatin accessibility markers have multidimensional prognostic value and are gradually moving from basic research to clinical practice.

In conclusion, these findings demonstrate the ability of dynamic regulation of chromatin accessibility in revealing the mechanisms of tumor progression and metastasis and as a prognostic biomarker of tumors, which provides new prospects for precision medicine in tumors.

### 4.2 Chromatin Accessibility as a Therapeutic Target

Given that chromatin accessibility abnormalities are closely associated with tumorigenesis, progression, and treatment resistance, targeting key nodes of the chromatin accessibility regulatory network (e.g., DNA methylation, histone modification, chromatin remodeling complexes, etc.) has become an emerging strategy for tumor therapy. These mechanisms and clinical potentials are systematically described in terms of the following five aspects.

#### 4.2.1 Targeting DNA Methylation

DNA methylation (e.g., CpG island hypermethylation) drives tumorigenesis by silencing oncogenes, and DNA methylation is dynamically regulated by DNMTs and demethylases, which have been identified as inhibitory targets for a variety of cancers.

DNMT inhibitor (DNMTi), such as azacitidine and decitabine, have been approved by the United States Food and Drug Administration (US FDA) for the treatment of myelodysplastic syndromes (MDS), AML, and chronic myelomonocytic leukemia [308, 309]. Beyond hematologic malignancies, DNMTis show promise in solid tumors [310]. For example, decitabine exhibits efficacy in preclinical models of castration‐resistant PC (CRPC) and NEPC, particularly in RB1‐deficient subtypes, suggesting biomarker‐driven strategies could improve outcomes [311]. Combining DNMTis with other therapies enhances efficacy. In OC, adenosine deaminase 1 (ADAR1) deletion synergizes with DNMTis to remodel the immune microenvironment, reducing tumor burden and extending survival [312]. Zebularine, another DNMTi, sensitizes tumors to immunotherapy by activating the cGAS–STING pathway [313].

In addition, combinations with other epigenetic drugs may provide better therapeutic effects. Rodems et al. showed that the DNMTis decitabine or guadecitabine (SGI‐110) enhanced antitumor immunity by increasing the accessibility and expression of HLA‐I in PC cells, when combining them with the HDAC inhibitor LBH‐589 (LBH) [314]. Zhang et al. tested the effects of the combination of DNMTi (5‐aza‐2′‐deoxycytidine) and the EZH2 inhibitor GSK126 on drug sensitivity, DNA methylation, nucleosome accessibility, and gene expression profiles in human HCC cell lines. An increase in the number of upregulated genes after combination therapy was associated with prolonged antiproliferative effects and increased nucleosome accessibility. And potential therapeutic targets were identified and provided a rationale for therapeutic efficacy in HCC patients [269].

Targeting DNA methylation modification factors provides a unique epigenetic intervention strategy for tumor therapy by remodeling chromatin accessibility. In the future, it is necessary to combine multiomics technologies to resolve methylation dynamics, develop highly selective inhibitors, and overcome drug resistance through combination therapy (e.g., immune checkpoint blockade, targeted metabolism) to ultimately achieve individualized epigenetic precision therapy.

#### 4.2.2 Targeted Histone Modifications

Histone modification is a central mechanism of epigenetic regulation that affects tumorigenesis, progression, and treatment resistance by dynamically altering chromatin structure and gene expression [315]. In recent years, small molecule inhibitors targeting histone modifying enzymes have become an important strategy for cancer therapy. In this section, we will explore representative drugs targeting histone methylation and acetylation with their mechanisms of action and discuss their clinical translational potential.

Histone methylation is coregulated by methyltransferases (HMT) and demethylases, and HMT plays a crucial role in many cellular processes and has been associated with different types of cancer [316]. Xie et al. found that SETD2, an RNA polymerase II (Pol II)‐associated HMT, catalyzes the cotranscriptional methylation of H3K36me2, and they established a tumor‐suppressor gene model in which SETD2‐mediated activation of an enhancer by H3K36me3 deletion drove the oncogenic transcriptional output by regulating chromatin accessibility. And a mechanism‐based therapeutic strategy for SETD2‐deficient cancers was discovered by targeting specific histone chaperone complexes, including ASF1A/B and SPT16, providing unique therapeutic opportunities [239].

EZH2 is the catalytic subunit of PRC2, which functions as a HMT, and its dysregulation may promote cancer development [317]. Tazemetostat is a first‐in‐class oral EZH2 inhibitor approved by the US FDA for the treatment of follicular lymphoma and epithelioid sarcoma [318]. A recent study revealed that EZH2‐mediated modification of H3K27me3 histone trimethylation decreased chromatin accessibility and that the EZH2 inhibitor Tazemetostat dose‐dependently decreased cell viability and increased lipid peroxidation in HCC cells. This result reveals a novel epigenetic mechanism controlling lipid peroxidation and iron death susceptibility in HCC and provides a theoretical basis for exploring EZH2‐targeted therapies against this malignancy [255]. Other EZH2 inhibitors valemetostat, CPI‐1205, and PF‐06821497 are currently in clinical development [319]. Ku et al. demonstrated that furazamidine (FM) inhibits the PRMT1 and reduces the expression of genes related to H4R3me2 modification, chromatin accessibility, and glycolysis thereby reversing chemoresistance in pancreatic cancer [214]. In addition, histone lysine demethylase (KDM) controls and maintains epigenetic factors that influence chromatin structure and cellular properties, and its dysregulation is associated with a variety of diseases, including malignancies. Zhang et al. showed that demethylase inhibitors of KDM2–7 (e.g., CBA‐1, JDI‐16, PKF118‐310, etc.) play an important role in the treatment of cancer, but the selectivity and intracellular activity of these inhibitors among subfamilies remains to be developed [320]. Although resistance and selectivity challenges still need to be addressed, we cannot deny that drugs targeting histone methylation have become an important strategy for cancer therapy.

PTMs of histones may play a critical role in cancer development and progression by regulating gene transcription, chromatin remodeling, and nuclear structure. Histone acetylation is a well‐studied posttranslational histone modification controlled by the opposing activities of HATs and HDACs, and HDAC inhibitors capable of reestablishing acetylation homeostasis may be useful in cancer therapy [321]. HDAC inhibitors are potent drug molecules that induce histone acetylation at lysine residues and induce open chromatin conformation at TSG loci, leading to tumor suppression [322]. The US FDA approves HDAC inhibitors for the treatment of certain cancers (e.g., T‐cell lymphomas), such as vorinostat (SAHA), romidepsin (i.e., depsipeptide, a bicyclic peptide), and belinostat. Panobinostat (LBH 589) has been approved for the treatment of MM [323]. In a recent study, Wang et al. found that the glycolytic enolase 2 (ENO2) constitutes a useful predictive biomarker and therapeutic target for resistance to antiangiogenic therapy in CRC and revealed a previously undefined and metabolism independent role of the ENO2‐derived metabolite phosphoenolpyruvic acid in regulating resistance to antiangiogenic therapy through its role as an endogenous HDAC1 inhibitor [324]. Yang et al. showed that PCI‐34051, a selective HDAC8 inhibitor, enhanced the efficacy of antitumor immunity and immune checkpoint blockade in HCC in a mouse model [325]. Similarly, Mormino et al. found that HDAC8 regulates human and mouse glioma cell viability and tumor migration through α‐microtubulin acetylation and inhibits NK cell‐mediated cytotoxic activity. Inhibition of HDAC8 by the specific inhibitor PCI‐34051 reduced tumor volume in a mouse model of glioma [326]. Psilopatis et al. indicated that HDAC inhibitors (apicidin, tricosuppressor TSA, and pabinostat LBH589, among others) inhibited tumor growth in vitro and in vivo, enhanced transcription of silenced physiological genes and induced cell cycle arrest and apoptosis in endometrial cancer cells, which could be a promising therapeutic alternative for endometrial cancer [327]. Jia et al. found that LAQ824, a novel pan‐HDAC inhibitor, inhibits PDAC progression and suppresses immune escape by promoting antigen presentation, providing a new strategy for targeting PDAC between epigenetic regulation and immunogenicity [328].

In addition, CBP/P300 is the most well‐studied acyltransferase that mediates multiple types of acylation on histones and nonhistone proteins. Nguyen et al. have determined that acK13–HOXB13 mediated by the HAT CBP/p300 is synergistic with the lineage specificity of a key depot‐resistant CRPC target and with the tumor‐promoting SE of H3K27 acetylation, thus acting as an epigenetic regulator of tumor growth. PSMA‐targeting agents and (R)‐9b may be novel therapeutic modalities for targeting HOXB13–ACK1 axis‐regulated PCs [329]. Welsh et al. showed that the P300 inhibitor GNE‐781, by blocking SE accessibility and inhibiting Myc, IRF4 expression, sensitizing MM to immunomodulatory imide drugs [330]. The above studies suggest that anticancer drugs targeting histone acetylation exert anticancer effects through global modulation of chromatin accessibility, but optimization of selectivity and combination therapy regimens is required.

Through these studies, we can determine that drugs targeting histone modification factors provide a new paradigm for cancer therapy by precisely regulating chromatin accessibility and gene expression. Although EZH2 and HDAC inhibitors, among others, have been successfully translated to the clinic, there is still a need to overcome drug resistance, optimize targeting, and explore combination strategies. Future studies should focus on subtype‐selective inhibitors and epigenetic–immunologic synergistic therapies to expand the beneficiary population.

#### 4.2.3 Targeted Chromatin Remodeling Complexes

Chromatin remodeling complexes can serve as emerging therapeutic targets by regulating chromatin structure and accessibility in an ATP‐dependent manner, affecting gene transcription, DNA repair, and cell fate decisions [331].

##### 4.2.3.1 Targeting SWI/SNF Complexes

The SWI/SNF complex facilitates the exposure of gene regulatory regions by sliding nucleosomes. Notably, mutations in genes encoding subunits of the SCRC are present in more than 20% of human cancers [331]. ARID1A is a core component of the SWI/SNF complex, and Cui et al. showed through their study that the inhibitor of arachidonic acid metabolism, aspirin, selectively inhibits the growth of ARID1A‐deficient CRCs, sensitizing tumors deficient in ARID1A to immunotherapy, providing a promising therapeutic strategy [332]. ARID1B is another ARID1 subfamily member, and ARID1A proteins are mutually exclusive components of the SWI/SNF complex [333]. Zhu et al. found that mutations in the SWI/SNF gene ARID1B lead to impaired DNA damage response and repair as well as altered chromatin accessibility. Notably, NSCLC patients harboring ARID1B mutations exhibited better OS and progression‐free survival following immune checkpoint inhibitor (ICI) therapy, indicating a potential predictive role for ARID1B mutations in ICI response. These findings shed light on the biological and therapeutic significance of ARID1B in lung cancer, emphasizing its potential as a target for precision medicine and immunotherapeutic strategies [306].

All SWI/SNF complexes contain the SMARCA family as the catalytic subunit of the ATPase that drives nucleosome sliding and expulsion [334]. A study on bladder cancer showed that loss of the SWI/SNF complex subunit SMARCB1 increased chromatin accessibility of the STAT3 locus in vitro and drove disease progression in bladder cancer patients. pSTAT3‐selective inhibitor TTI‐101 reduced xenografts originating from the SMARCB1 KO cell line of origin and xenotransplantation originating from SMARCB1‐deficient patient tumor growth in the model and demonstrated in several preclinical models that targeting the IL6/JAK/STAT3 molecular pathway is a potential therapeutic approach for SMARCB1‐deficient bladder cancer [243]. Redin et al. identified a role for SMARCA4, the catalytic subunit of the SWI/SNF complex, as a regulator of metastasis in subtypes of SCLC, with altered chromatin accessibility and enhances NE programs. In addition, the SMARCA4 inhibitor FHD‐286 (Foghorn Therapeutics) drives ERBB pathway activation in SCLC and sensitizes SCLC tumors to afatinib. Ultimately, they designated SMARCA4 as a key regulator of NE state plasticity and identified novel therapeutic strategies for SCLC [335]. With regard to FHD‐286, another study found it to be an orally bioavailable and selective BRG1/BRM inhibitor, which reduced AML burden, improved survival, and attenuated the AML initiation potential of stem cell progenitor cells [336].

Other studies have found that PFI‐3, a recently developed bromodomain inhibitor specifically targeting SWI/SNF chromatin remodelers, effectively blocked chromatin binding of its target bromodomain and dissociated the corresponding SWI/SNF proteins from chromatin, sensitizing several human cancer cell lines to DNA damage induced by chemotherapeutic agents such as adriamycin [337]. Panditharatna et al. inhibited glioma progression by linking the core of the BRG1 ATPase inhibitor to phthalimide and creating a degrader, JQ‐dS‐4. A new strategy for epigenetically targeted therapy of pediatric gliomas targeting the lethal type of ATPase activity of the BAF complex is shown [338]. In conclusion, SWI/SNF subunits are potential therapeutic targets for a wide range of cancers, and further understanding of the exact role of SWI/SNF complex subunits in cancer is needed to further develop new strategies against drug resistance and targeting specificity and to address precision therapy in the context of complex mutations.

##### 4.2.3.2 Targeting Other Complexes

In addition, targeting therapy regarding other chromatin remodeling complexes has been partially investigated in recent years. Chromatin structural domain deconjugating enzyme DNA‐binding protein 4 (CHD4) is a core member of the NuRD complex, and Oyama et al. noted that the dual SMARCA5/CHD4 inhibitor ED2–AD101 sensitized OC cells to cisplatin by decreasing the expression of multidrug resistance 1 (MDR1) [339]. Graca Marques et al. found that the NuRD subunit CHD4 serves as a therapeutic target for Ewing sarcoma, and that CHD4 inhibitors led to an overall increase in DNA accessibility and induction of spontaneous DNA damage, resulting in increased susceptibility to DNA damaging agents. This resulted in inhibition of tumor growth and improved OS [340]. Xu et al. found that BPTF (the largest subunit of the ISW1 complex) is a chromatin remodeling factor in NSCLC, and that an inhibitor of the bromodomain of BPTF called C620‐0696, which inhibits the progression of NSCLC primarily by inhibiting c‐Myc transcription [341]. Nano et al. indicated that sorafenib, a potent inhibitor of RUVBL1 and RUVBL2, which are important members of the chromatin remodeling complex INO80, can inhibit the ATPase activity of the RUVBL1/2 complex, which provides research support for targeting chromatin remodeling complexes [342].

Therefore, we believe that the strategy of targeting chromatin remodeling complexes provides a new dimension for cancer therapy by modulating chromatin accessibility. And more research is still needed in the future to drive the progress of this field toward personalized therapy.

#### 4.2.4 Targeted Transcription Factors

Transcription factors play a crucial role in determining the fate of cells during development and cancer progression [343]. In recent years epigenetic drugs have become an emerging strategy for cancer therapy by targeting the functional domains of transcription factors or modulating the epigenetic modifications they bind.

Li et al. observed that CRC cells metastasized to the liver showed enrichment of HNF4A, a liver‐specific transcription factor belonging to the HNF family. The epigenetic shift favoring liver tropism and its effects on immune response, metabolism, and malignancy heterogeneity of liver metastatic CRC cells at single‐cell resolution were elucidated. These findings reveal a critical role for HNF4A, which may provide an important therapeutic target for liver metastasis and enhancement of immunotherapeutic response in CRC patients [240]. Wang et al. showed that the transcriptional repressor ZBTB18 is a potent chromatin modulator, and loss of its activity enhances chromatin accessibility and transcriptional adaptation, thereby promoting phenotypic changes required for metastasis. In contrast, restoration of ZBTB18 activity reduces chromatin accessibility to promoters of genes that drive metastasis, such as Tgfbr2, thereby preventing activation of the TGFβ1 pathway, which in turn reduces cell migration and invasion [344].

In terms of specific drugs, Holmes’ study led to the discovery of MYC as a transcription factor, and its inhibitor MYCi975 selectively altered the MYC and MAX cis‐groups and modulated the epigenomic landscape to regulate target gene expression, sensitizing drug‐resistant PC cells to ENZ, and estrogen‐receptor‐positive breast cancer cells to 4‐hydroxytamoxifen [345]. Chen et al. found that the master transcription factor ONECUT2 regulates gene expression by a mechanism that includes promoter binding, enhanced genome‐wide chromatin accessibility, and SE reprogramming. The OC2 inhibitor CSRM‐617 suppressed the genealogical plasticity reprogramming induced by the AR signaling inhibitor ENZ, and targeted inhibition of OC2 may play an important role in blocking or delaying the emergence of desmoplasma‐resistant PC [285]. In a similar study, Leppanen et al. found that the AR inhibitor ENZ increased chromatin accessibility and expression of the cognate frameshift transcription factor SIX2, which promotes stem‐like reprogramming, NE differentiation, and AR inhibitor resistance, suggesting that targeted SIX2 inhibition may represent a promising approach to overcoming the cellular plasticity mechanisms a promising therapeutic strategy; however, as a transcription factor, direct inhibition of SIX2 may be challenging [286].

Through these studies, we can easily see that epigenetic drugs targeting transcription factors provide new options for cancer therapy by interfering with transcription complex assembly or function and specifically modulating chromatin accessibility of oncogenes.

#### 4.2.5 Targeting ncRNAs

Although ncRNAs do not encode proteins, they play a key role in epigenetic regulation. In recent years, epigenetic drugs targeting ncRNAs have provided new directions for precision cancer therapy by directly or indirectly modulating their functions. Based on the latest research advances, this section discusses the clinical applications of these drugs and the challenges associated with their translation.

Tang et al. reported that LINC01057 as lncRNA is a regulator of NF‐κB signaling, leads to effective chromatin accessibility at NF‐κB‐responsive promoters, promotes MES differentiation, and is a potential target for therapeutic intervention in the MES subtype of GBM. LINC01057 inhibition suppresses proliferation, invasion, and radioresistance of GBM cells in vitro and in vivo inhibited tumor growth [346]. Zhang et al. identified LINC01977 as a cancer testis lncRNA that was hijacked by SEs, which promoted proliferation and invasion in vitro and in vivo. In early‐stage LUAD, higher chromatin accessibility and high TGF‐β expression were observed in the SE region of LINC01977, and they suggested that LINC01977 hijacked by SE may be a valuable therapeutic target, especially for the treatment of early‐stage LUAD [231]. A study on breast cancer progression found that Malat1 lncRNA plays a key role in regulating breast cancer pathogenesis, and that treatment of Malat1‐specific ASO resulted in a dramatic shift of breast tumors to a highly differentiated, less invasive state, representing a potential therapy to inhibit breast cancer progression. It also indicates that future use of patient‐derived xenograft models as well as organoids can further investigate the efficacy of Malat1 ASO as a therapeutic agent to reduce tumor progression [347]. Ye et al. discovered a novel magnetic thermosensitive cationic liposome drug carrier for codelivery of oxaliplatin (OXA) and antisense lncRNA of MDC1 (MDC1‐AS) to cervical cancer cells, which enhances in vitro and in vivo inhibition of the growth of cervical cancer cells and serves as a novel targeted therapeutic system for cervical cancer [348]. Several other studies have also corroborated that circRNA vaccines and circRNA‐based therapeutic platforms have superior applications in the treatment of melanoma [349, 350, 351].

The prospect of targeting ncRNAs for the treatment of cancer is clear, although more research is needed in the future that needs to be further validated to drive the translation of this field to the clinic.

The above multiple studies on therapeutic targets suggest that epigenetic drugs targeting chromatin accessibility provide a multidimensional intervention strategy for cancer therapy by intervening in DNA methylation, histone modification, chromatin remodeling, transcription factors, and ncRNAs. Despite the challenges of selectivity and drug resistance, the development of precision editing technologies and the integration of multiomics analyses hold great promise for translating personalized epigenetic therapies based on chromatin dynamics into clinical practice in the future. This advancement is expected to drive cancer treatment into the “era of epigenetic precision.” Representative drugs targeting different targets are summarized in Table 1.

**TABLE 1:** Representative epigenetic drugs based on different targets for cancer treatment.

Target	Drugs	Mechanism	Function	References
DNMTs	Azacitidine	Nucleoside analogs that irreversibly bind to DNMT	Reversing DNA hypermethylation, inducing DNA damage, and inhibiting cancer progression	[352]
DNMTs	Decitabine	Nucleoside analogs that bind irreversibly to DNMT	Reverses aberrant DNA hypermethylation to exert antitumor effects	[269, 311]
DNMTs	Zebularine	Nucleoside analogs, inhibits DNMT and cytidine deaminase	Sensitize cGAS–STING pathway to enhance antitumor immunity	[313]
DNMTs	Guadecitabine	Nucleoside analog, inhibits DNMT	Increase HLA‐I accessibility and expression to inhibit prostate cancer	[314]
DNMT3B	Nanaomycin A	Binds to the catalytic site of DNMT3B	Sensitize HCC cells to sorafenib	[353]
DNMT1	MG‐98	Antisense oligodeoxyribonucleotide	Antitumor activity, well tolerated	[354]
DNMT1	GSK3685032	Selective inhibitor of DNMT1, competes with the active site loop of DNMT1	Induces DNA methylation deletion, transcriptional activation, and cancer cell growth inhibition	[355]
HDACs	Vorinostat	Competitive binding to the catalytic site of HDAC	Induces apoptosis, impedes cell cycle progression, and inhibits cancer cell proliferation	[356, 357]
HDACs	Romidepsin	Interaction near the active site	Induces apoptosis and inhibits cancer progression	[358]
HDACs	Belinostat	Prevents acetyl group removal	Induces cell cycle arrest and reduces tumor cell proliferation	[359]
HDACs	Panobinostat	Inhibits aggregation of misfolded proteins and disrupts the cell cycle	Induces metabolic reprogramming and inhibits cancer progression	[314, 360]
HDACs	Trichostatin A	Competitively binds to the catalytic site of HDAC	Inhibit the growth of different cancer cells through cycle arrest and apoptosis	[357, 361]
HDAC8	PCI‐34051	Selective HDAC8 inhibitor	Enhances antitumor immunity and immune checkpoint blockade in hepatocellular carcinoma	[325]
HDAC1/2	MGCD0103	Inhibits benzamide group‐dependent HDAC, induces hyperacetylation of histones	Induction of apoptosis with broad‐spectrum antitumor activity	[362]
HDAC1	CI‐994	Class I‐specific HDACi, inhibits benzamide moiety‐dependent HDAC	Inhibits proliferation and induces apoptosis in vitro and in vivo	[363]
HDAC1/2/3/10	Tucidinostat	Novel benzamide‐based histone deacetylase inhibitor	Remodels tumor epigenome and activates antitumor immunity	[364]
HDACs	LAQ824	Novel pan‐histone deacetylase inhibitor	Inhibits tumor proliferation, inhibits epithelial–mesenchymal transition and induces apoptosis	[328]
HMT	Tazemetostat	Specifically inhibits the methyltransferase activity of EZH2	Reduces H3K27me3 levels and decreases HCC cell viability	[255]
HMT	Valemetostat	EZH1/EZH2 dual‐target inhibitors	Reduce H3K27me3 level more thoroughly, overcome single target drug resistance	[365]
HMT	GSK126	Highly selective and potent EZH2 inhibitor, competes with S‐adenosyl‐methionine	Upregulates tumor suppressor genes to inhibit HCC	[269]
HMT	Pinometostat	DOT1L inhibitor, competitively binds to S‐adenosyl‐methionine binding site	Blocked H3K79 methylation and inhibited MLL fusion protein‐driven leukemia cell proliferation	[366]
HMT	UNC0642	Inhibits G9a/GLP activity, reduces H3K9me2 labeling	Reduce the level of H3K9me2, restore oncogenic factors, and reduce the expression of oncogenic related proteins	[367]
PRMT	Furamidine	Targets enzyme active domains	Inhibit pancreatic tumor growth and reverse chemotherapy resistance in pancreatic cancer	[214]
PRMT	EPZ015666	Highly selective PRMT5 inhibitor, competitive inhibitor of S‐adenosyl‐methionine	Antitumor activity through reprogramming of T‐cell‐mediated responses	[368]
HDM	Phenelzine	Monoamine oxidase‐A inhibitor	Antidepressant, also found to reverse enzalutamide resistance in desmoplasia‐resistant prostate cancer	[369]
HAT	C646	Competitively binds p300/CBP catalytic domain and inhibits H3K27ac	Inhibits LINC00501 levels and inhibits gastric cancer growth in vitro and in vivo	[370]
HAT	A‐485	Highly selective p300/CBP catalytic domain inhibitor	Attenuates glycine/serine metabolism and inhibits proliferation of hepatocellular carcinoma cells	[371]
BRD/BET	Pelabresib	Binds to the bromodomain of BRD4 and blocks its binding to acetylated histones	Potent cytotoxicity, limiting tumor cell proliferation and survival	[372]
BRD/BET	Molibresib	Competitively binds to the bromo domain of BET proteins	Combination therapy with endocrine therapy may overcome endocrine resistance	[373]
BRD/BET	JQ1	Blocks the binding of BRD4 to acetylated histones by blocking its binding	Inhibits tumor growth by decreasing c‐Myc expression in endometrial cancer	[374]
SMARCA2/4	FHD‐286	Binds to the ATPase domain of SMARCA2/4 and inhibits chromatin unwinding and gene transcription activation	Induces loss of neuroendocrine features in lung cancer and sensitizes SCLC tumors to afatinib	[335]
SMARCA2/4	AU‐15330	PROTAC degrader of SMARCA2/4 that selectively degrades target proteins via the ubiquitin–proteasome system	Eliminates the structure of cis‐regulatory elements that bind to CHD6 and inhibits renal cancer growth	[375]
SMARCA2/4	PFI‐3	Binds to the bromodomain of SMARCA2/4 and prevents its binding to acetylated histones	Targeting SWI/SNF sensitizes cancer cells to DNA damage	[337]
SMARCA2	A947	Selective SMARCA2 protein hydrolysis targets chimeric molecules	Inhibited SMARCA4 mutant solid tumors	[376]
SMARCA4	JQ‐dS‐4	Competitive binding to the bromo domain of BRD4 blocks its binding to acetylated histones	Inhibits progression of gliomas	[338]
SMARCA2/4	ADAADi	Inhibitor of the ATPase structural domain of ATP‐dependent chromatin remodeling proteins	Ability to block migration and invasiveness of cancer cells and promote apoptosis of cancer cells	[377]
CHD4	ED2–AD101	Dual‐targeted inhibitor of CHD4/SMARCA5	Sensitized ovarian cancer cells to cisplatin	[339]
BPTF	C620‐0696	Inhibitor of the BPTF bromodomain	Inhibits NSCLC progression by inhibiting c‐Myc transcription	[341]
MYC	MYCi975	Binds directly to MYC and disrupts MYC/MAX dimerization	Sensitize drug‐resistant prostate cancer cells to enzalutamide	[345]
ONECUT2	CSRM‐617	Suppresses lineage plasticity reprogramming induced by enzalutamide	Blocking or delaying the emergence of desmoplasia‐resistant prostate cancer	[285]

### 4.3 Chromatin Accessibility in Combination Therapy

**FIGURE 4:**
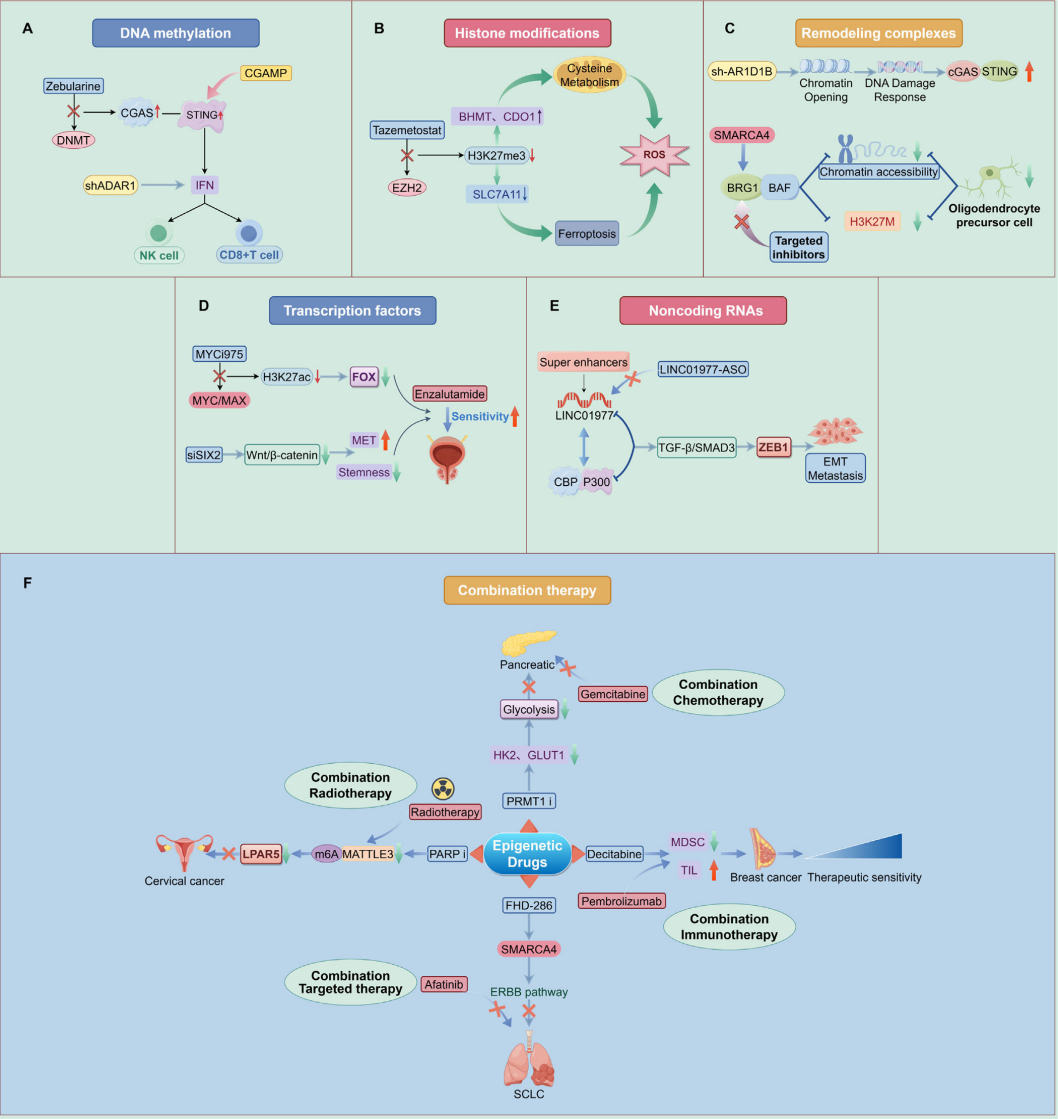
Chromatin accessibility in cancer therapeutic targets and combination therapy strategies. (A) Targeting DNA methylation: Zebularine specifically sensitizes the cGAS–STING pathway and promotes CD8 T‐cell and NK‐cell infiltration, thereby enhancing antitumor immunity. (B) Targeting histone modification: The EZH2 inhibitor Tazemetostat enhances lipid ROS accumulation by epigenetically modulating genes associated with cysteine metabolism and ferroptosis suppression. (C) Targeting chromatin remodeling complexes: ARID1B deficiency leads to impaired DNA damage response and activation of the cGAS–STING pathway in NSCLC; an inhibitor targeting the BRG1–BAF complex reduces the level of H3K27M, which reduces the maintenance of glioma stem cells in an oligodendrocyte precursor cell‐like state. (D) Targeting transcription factors: (1) MYCi975 disrupts the dimerization structure of MYC and MAX, thereby reducing H3K27ac levels and target gene expression; (2) targeting inhibitors of SIX2 downregulates the Wnt/β‐catenin pathway and induces mesenchymal–epithelial transition (MET). These two mechanisms sensitize drug‐resistant prostate cancer to enzalutamide. (E) Targeting noncoding RNA: Inhibition of LINC01977 hijacked by a super‐enhancer reduces epithelial–mesenchymal transition by downregulating the TGF‐β/SMAD3 pathway. (F) Combination of epigenetic drugs with chemotherapy, immunotherapy, targeted therapy, and radiotherapy.

Based on the above review, we can see that chromatin‐accessible targeted therapeutics have become an important strategy for cancer treatment by modulating epigenetic modifications to remodel chromatin structure. However, there are still challenges such as selectivity and drug resistance. Therefore, the research direction in recent years has also gradually favored its combination with chemotherapy, immunotherapy, targeted therapy, and radiotherapy to exert synergistic effects (Figure 4). We also summarized current clinical trials of different epigenetic drugs for combination therapy of cancer (Table 2).

**TABLE 2:** Clinical trials of epigenetic drugs in combination with other treatments.

	Epigenetic drug	Combination treatment	Clinical trial ID	Cancer type(s)	Status	Phase of clinical trial
Combination chemotherapy	Azacitidine or decitabine	Cytarabine, doxorubicin, etoposide, etc.	NCT03164057	AML	Active, not recruiting	II
	Decitabine	Cytarabine	NCT03417427	AML	Unknown	II
	Azacytidine	Fludarabine, cytarabine	NCT01861002	ALL, AML	Completed	I
	Decitabine	Tetrahydrouridine	NCT02847000	Pancreatic cancer	Completed	I
	Chidamide	Epirubicin, cyclophosphamide, docetaxel	NCT05400993	Breast cancer	Unknown	II
	Decitabine, vorinostat	Fludarabine, cytarabine	NCT03263936	AML	Completed	I
	Decitabine	Idarubicin, cytarabine	NCT01607645	AML, MDS	Terminated	II
	Decitabine, panobinostat	Temozolomide	NCT00925132	Metastatic melanoma	Terminated	I/II
	Azacitidine	Homoharringtonie, cytarabine	NCT04248595	AML	Unknown	II
	CC‐486	Abraxane	NCT01933061	Metastatic melanoma	Withdrawn	II
	5‐Azacitidine	Carboplatin, paclitaxel	NCT01209520	NSCLC	Completed	Not Applicable
	RRx‐001	Cisplatin, etoposide	NCT02489903	NSCLC, ovarian cancer	Completed	II
	Decitabine	Cytarabine, etoposide, mitoxantrone hydrochloride	NCT01729845	AML, myelodysplastic syndromes	Completed	I/II
	RRx‐001	Gemcitabine, cisplatin	NCT02452970	Advanced cholangiocarcinoma	Terminated	II
	Decitabine	Daunorubicin, cytarabine	NCT02085408	AML	Completed	III
	Chidamide	CMOP regimen	NCT05896813	Peripheral T‐cell lymphoma	Unknown	Not Applicable
	Decitabine	Temozolomide	NCT00715793	Metastatic melanoma	Completed	I/II
Combination immunotherapy	Chidamide, azacitidine	Sintilimab	NCT05008666	ENKTL	Unknown	II
	Decitabine	Nivolumab	NCT02664181	NSCLC	Completed	II
	Chidamide	Anti‐PD‐1 antibody	NCT04414969	NK/​T cell lymphoma	Recruiting	II
	Guadecitabine	Pembrolizumab	NCT03220477	Advanced lung cancer	Active, not recruiting	I
	Guadecitabine	Atezolizumab	NCT03179943	Urothelial carcinoma	Active, not recruiting	II
	Tetrahydrouridine–decitabine	Nivolumab	NCT02664181	NSCLC	Completed	II
	EDO‐S101	Nivolumab	NCT03903458	Advanced melanoma	Unknown	I
	SGI‐110	Ipilimumab	NCT02608437	Metastatic melanoma	Unknown	I
	Azacitidine	Pembrolizumab	NCT02959437	Colorectal cancer, NSCLC	Terminated	I/II
	Guadecitabine	Atezolizumab	NCT03206047	Ovarian, Fallopian tube, primary peritoneal cancer	Completed	I/II
	CC‐486	Pembrolizumab	NCT02546986	NSCLC	Active, not recruiting	II
	Decitabine	Pembrolizumab	NCT03233724	NSCLC, esophageal carcinomas	Terminated	I/II
	Decitabine	Nivolumab	NCT05089370	Mucosal melanoma	Active, not recruiting	I/II
Combination targeted therapy	Decitabine	Eltrombopag	NCT02446145	AML	Terminated	II
	Decitabine	Anlotinib	NCT04611711	Digestive system tumors	Unknown	I/II
Combination radiotherapy	Decitabine	Radiotherapy	NCT01707004	AML	Completed	II
	Vorinostat	Radiotherapy	NCT00821951	NSCLC	Completed	I
	Vorinostat	Radiotherapy	NCT00838929	Brain metastases	Completed	I
	Panobinostat	Radiotherapy	NCT00670553	Prostate, esophageal, head and neck	Completed	I
Combination endocrine therapy	Decitabine, LBH589	Tamoxifen	NCT01194908	Breast cancer	Terminated	I/II
	CC‐486	Fulvestrant	NCT02374099	Metastatic breast cancer	Terminated	II
	Decitabine	Enzalutamide	NCT03709550	Metastatic castration resistant prostate cancer	Withdrawn	I/II
Multiple combined therapy	Azacytidine	Carboplatin, paclitaxel, durvalumab, etc.	NCT06694454	NSCLC	Not yet recruiting	I/II
	Azacitidine	Avelumab, utomilumab, gemcitabine, oxaliplatin	NCT02951156	DLBCL	Terminated	III
	Decitabine	Adebrelimab, paclitaxel, gemcitabine	NCT06454448	Metastatic pancreatic cancer	Recruiting	I/II
	Chidamide	Rituximab, gemcitabine plus oxaliplatin	NCT04022005	DLBCL	Completed	II
	Azacytidine	Carboplatin, paclitaxel, durvalumab	NCT06694454	NSCLC	Not yet recruiting	I/II
	Vorinostat	Pembrolizumab, tamoxifen	NCT04190056	Breast cancer	Terminated	II
	CC‐486	nab‐Paclitaxel IV, duravalumab	NCT02250326	NSCLC	Completed	II
	Decitabine	Radiotherapy, pembrolizumab	NCT03445858	Solid tumors, lymphoma	Active, not recruiting	I
	Vorinostat	Radiotherapy, temozolomide	NCT00731731	Glioblastoma multiforme	Completed	I/II
	Tazemetostat	Radiotherapy, docetaxel	NCT05151588	Sinonasal carcinoma	Not yet recruiting	II
	Vorinostat	Radiotherapy, temsirolimus	NCT02420613	Diffuse intrinsic pontine glioma	Completed	I
	Vorinostat	Radiotherapy, cisplatin	NCT05608369	Head and neck squamous cell carcinoma	Withdrawn	II
	Belinostat	Radiotherapy, temozolomide	NCT02137759	GBM	Unknown	II
	Tazemetostat	Radiotherapy, docetaxel, 5‐FU	NCT05151588	Sinonasal carcinoma	Not yet recruiting	II
	Azacitidine, romidepsin	nab‐Paclitaxel, gemcitabine, durvalumab	NCT04257448	PDAC	Unknown	I/II
	Chidamide	Regorafenib, iparomlimab, tuvonralimab	NCT06930118	Advanced colorectal cancer	Recruiting	II

NIH clinical trial database: www.clinicaltrials.gov.

#### 4.3.1 Combination Chemotherapy

In terms of combination chemotherapy, Gu et al. found that the PRMT1 controls chromatin accessibility related to cancer cell metabolism and is a key regulator of pancreatic cancer development. Combining its inhibitors (FM and TCE) with gemcitabine represents an effective therapeutic approach for patients with pancreatic cancer, particularly those with Gem resistance [214]. Following a study by Oyama et al. who found that CHD4 mediates platinum sensitization by modulating MDR1 expression in OC, the CHD4/SMARCA5 inhibitor ED2–AD101 and cisplatin showed synergistic effects, suggesting that CHD4 inhibition has the potential to be a novel therapeutic strategy in combination with platinum agents [339]. Brown et al. found that sequential treatment with DNMTi azacitidine and carboplatin slowed high‐grade plasmacytoid OC cell growth, as well as demethylated and upregulated pathways involved in the immune response, suggesting that this combination could be used to increase the response of patients who are resistant to multiple therapies, such as platinum‐based ICIs [378].

#### 4.3.2 Combination Immunotherapy

In terms of combination immunotherapy, Weng et al. found that the DNMTi decitabine enhanced the formation of immune synapses between ex vivo expanded allogeneic γδ T cells and cancer cells and enhanced the antitumor immunity of γδ T cells. This result supports the potential of combining DNMTis with γδ T cell‐based immunotherapy for lung cancer treatment [379]. Li et al. used a mouse model to show that the combination of decitabine and anti‐PD‐L1 therapy was effective in reducing RCC tumor load, particularly in SETD2‐deficient renal cancers, which underscores the synergistic potential [380]. Similarly, Zhang et al. found that DNMTi zebularine stimulated cGAS–STING–NF‐κB/IFNβ signaling to enhance tumor cell immunogenicity, thereby promoting effective CD4+ and CD8+ T cell‐mediated tumor cell killing. This finding supports the use of combination regimens including DNMTi and immunotherapy for cancer treatment [381]. Bear found that treatment with decitabine and pembrolizumab in the neoadjuvant preadjuvant window sensitized breast cancer to standard NCT by recruiting TIL to tumor tissue and was well tolerated by treatment [382].

#### 4.3.3 Combination Targeted Therapy

In terms of combination targeted therapy, Redin et al. found that the SMARCA4 inhibitor FHD‐286, a subunit of the SWI/SNF complex, in combination with afatinib, showed significant effects in slowing SCLC tumor growth compared with the two drugs alone, even in PDX originating from tumors after several lines of treatment, supporting the potential of this combination as a therapeutic strategy for SCLC [335]. Shim et al. evaluated a combination strategy of olaparib and DNMTi 5‐AZA in epithelial OC (EOC) cells with a significant antitumor effect, suggesting that this combination therapy may be a potential therapeutic strategy for EOC [383]. Lai et al. found that the selective DNMT3b inhibitor, nanomycin A, significantly increased the sensitivity of HCC cells to sorafenib, and that targeting DNMT3b showed synergistic effects with sorafenib in the treatment of sorafenib‐resistant HCC, providing a therapeutic strategy for patients with HCC that expresses cancer stemness traits [353]. After Gomez's study, it was found that the DNMTs and the ADAR1 coinhibition reduced tumor load and prolonged survival in an immunocompetent mouse model of OC. This suggests that combining epigenetically induced transposable element transcription with inhibition of RNA editing represents another novel therapeutic strategy [312].

#### 4.3.4 Combination Radiotherapy

In terms of combination radiotherapy, Sun et al. showed that NMS‐P118, an inhibitor targeting METTL3, inhibited the growth of xenograft tumors in mice and exhibited synergistic effects with in vivo radiotherapy, establishing a novel radiotherapy combination therapy strategy by inactivating the PARP‐1–METTL3–LPAR5 axis [384]. Abbotts et al. found that DNMTis induced a BRCAness phenotype that sensitized NSCLC to PARP inhibitors and ionizing radiation [385]. Ullrich et al. found that decitabine alone and in combination with valproic acid VPA increased SSTR2 levels in pheochromocytomas as well as uptake of radiotracer in vitro, a study that demonstrated that epigenetic modifiers and peptide receptor radionuclide therapy combination has potential for clinical application [386].

**FIGURE 5:**
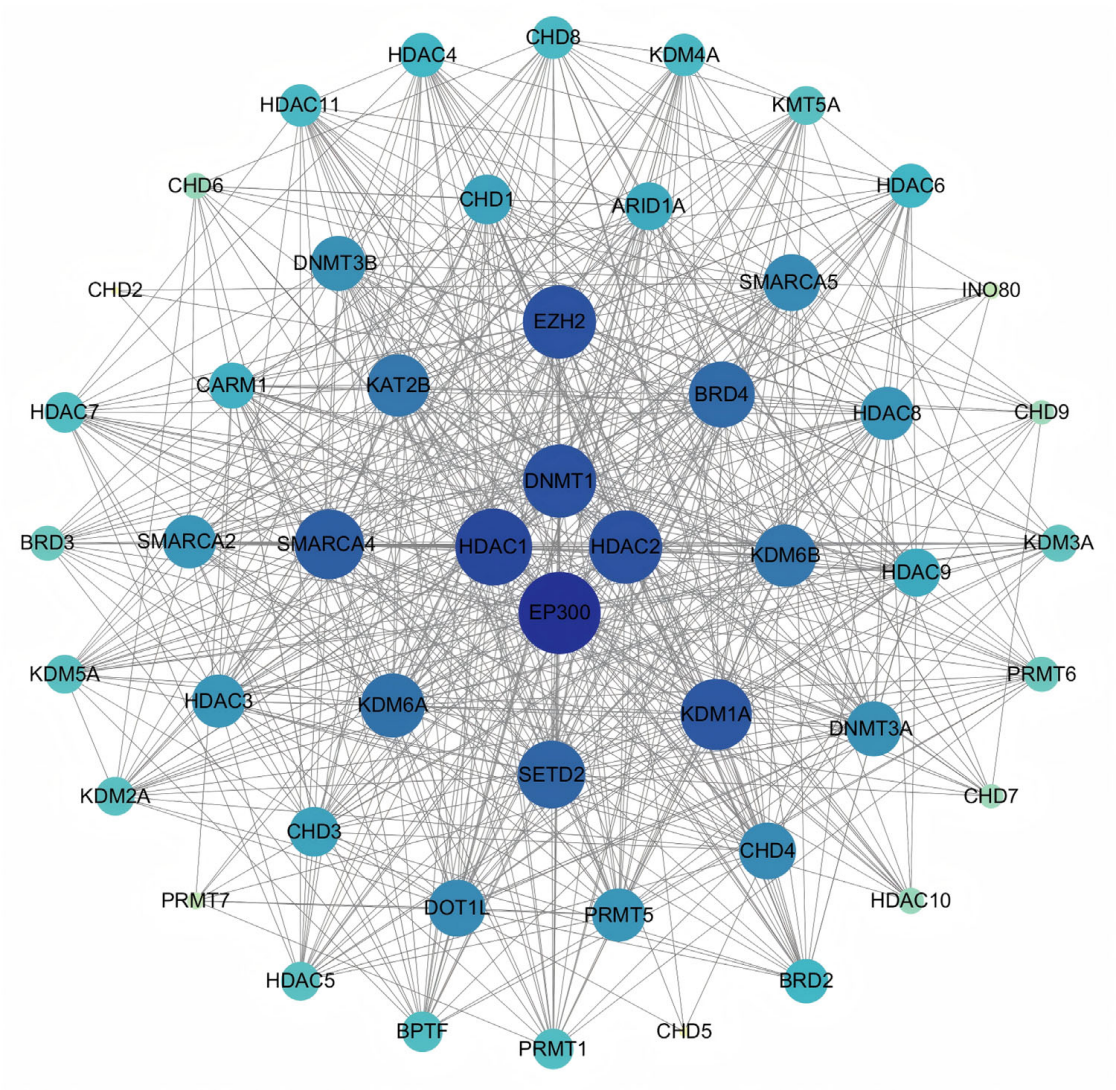
Protein–protein interaction (PPI) network involving distinct proteins affecting chromatin accessibility. This protein interaction network was constructed based on the STRING database, encompassing 50 core proteins extensively studied to date (including those frequently cited herein). The roles of these proteins in regulating chromatin accessibility have been thoroughly investigated. Each node represents a protein; larger nodes typically denote hub proteins within the network, potentially possessing significant regulatory functions. Connecting lines symbolize interactions between proteins, with thicker lines indicating stronger and more reliable evidence supporting the interaction.

Collectively, these findings indicate that combining chromatin accessibility‐targeted agents with conventional therapies markedly improves anticancer efficacy through multimechanistic synergy. Preclinical and early clinical trials have validated its potential, but further solutions are needed to address toxicity management, drug resistance mechanisms, and individualized treatment regimen design. Future research should focus on precise combination strategies and translational biomarkers to advance this field into clinical practice. Furthermore, we utilized the STRING database (https://string‐db.org/) to investigate the interactions between certain key proteins or molecules involved in regulating chromatin accessibility in this study (Figure 5).

Therapeutic targeting of chromatin accessibility is rapidly advancing from preclinical validation toward clinical application. To comprehend the efficacy, limitations, and translational potential of these strategies, critical evaluations across diverse model systems are required. Mouse models (e.g., gene‐edited mice, PDX) have played a vital role in elucidating mechanisms and assessing in vivo efficacy, while emerging human models such as PDOs and 3D bioprinted tumors replicate human tumor biology and microenvironmental features with unprecedented precision. The table below synthesizes findings from these diverse models, highlighting therapeutic principles, species‐specific variations, and the critical role of advanced human models in mitigating clinical translation risks (Table 3).

**TABLE 3:** Therapeutic strategies targeting chromatin accessibility: a crossmodel comparison.

Therapeutic target/strategy	Cancer type/process	Key findings in different models	Consensus and discrepancies	Potential implications/translational insights
Targeting DNA methylation				
DNA methyltransferase inhibitors (DNMTis)	Myelodysplastic syndrome/acute myeloid leukemia	Human clinical trials: Azacitidine and decitabine have been approved for the treatment of MDS and AML, reactivating silenced tumor suppressor genes through hypomethylation [387].	Consensus: DNMTis exert antitumor effects through demethylation in both human and mouse models.	Human‐derived models such as PDOs can better predict clinical responses and drug resistance, thereby guiding patient stratification.
		Mouse model: In an AML mouse model, DNMT inhibitors were demonstrated to induce tumor differentiation and prolong survival [388].	Differences: Mouse models often demonstrate more pronounced efficacy to DNMT inhibitors than human patients, suggesting that the tumor microenvironment and heterogeneity in human tumors are more complex.	
		Patient‐derived organoids (PDOs): Leukemia PDOs are employed to assess sensitivity to DNMT inhibitors and to identify mechanisms of resistance [389].		
Targeting histone modifications				
BET bromodomain inhibitor	Lymphoma/multiple myeloma/colon cancer	Human clinical trials: Preliminary efficacy has been demonstrated in multiple myeloma and lymphoma, though single‐agent activity remains limited and resistance develops readily [390].	Consensus: BET proteins are key transcriptional coactivators, and their inhibition suppresses tumor growth across multiple models.	The value of employing advanced human models, such as 3D models, for testing drug permeability and combination strategies prior to clinical trials has been emphasized.
		Mouse model: In lymphoma patient‐derived xenograft (PDX) models, BET inhibitors effectively downregulate the expression of key oncogenes such as MYC [391].	Discrepancies: The toxicity and drug resistance observed in human clinical trials prove difficult to fully replicate in mouse models.	
		3D bioprinted models: For evaluating the permeability and efficacy of epigenetic drugs within simulated tumor spatial structures [392].		
Targeting chromatin remodeling complexes				
SWI/SNF complex (e.g., ARID1A deficiency)	Breast cancer/ovarian cancer/colorectal cancer	Human studies: ARID1A mutations occur in certain breast cancers, leading to altered chromatin accessibility and potentially conferring therapeutic vulnerability [393].	Consensus: Deletion of SWI/SNF complex subunits creates novel therapeutic dependencies in humans and mice.	Provides a theoretical foundation for precision therapies based on synthetic lethality. PDOs can be employed to validate the efficacy of treatments targeting mutations in specific chromatin remodeling complexes.
		Mouse model: Synthetic lethality with EZH2 inhibitors was validated in an ARID1A‐deficient ovarian cancer mouse model [394].	Differences: The intensity and specificity of synthetic lethal effects may vary depending on genetic background and cellular environment, necessitating validation in human‐derived models.	
		Colorectal cancer PDOs: Research has confirmed that chromatin remodeling complexes are key drivers of colorectal cancer progression and represent potential therapeutic targets [395].		
Targeting transcription factors				
AP‐1 transcription factor family	Colorectal cancer	Human organoid research: Utilizing colorectal cancer CiPDOs models, AP‐1 has been identified as pivotal in maintaining tumor oncofetal state plasticity. Inhibiting AP‐1 diminishes this plasticity whilst enhancing chemotherapy sensitivity [396].	Consensus: Transcription factors such as AP‐1 play a central role in driving tumor plasticity and malignant progression.	CiPDOs provide a unique platform for investigating human‐specific transcription factor targets associated with cellular state plasticity.
		Mouse models: Frequently employed to validate the in vivo function of transcription factors in tumorigenesis and tumor progression.	Differences: The CiPDOs model has for the first time stably captured a human CRC‐specific “fetal‐like state” in vitro, revealing the unique and pivotal role of AP‐1 within it. This particular cellular state proves difficult to maintain stably over extended periods and study effectively in mouse models.	
NF‐κB pathway	Triple‐negative breast cancer	Human cell lines/mouse models: Research indicates that the NF‐κB pathway mediates chemotherapy resistance in triple‐negative breast cancer (TNBC). Inhibiting this pathway downregulates cellular activity and enhances chemotherapy sensitivity [397].	Consensus: NF‐κB is a key pathway in promoting survival and inflammatory responses, and its activation is associated with therapeutic resistance.	Combination therapies or local tumor delivery strategies may represent more viable approaches for targeting such key pathways.
			Differences: The pathway's critical role in normal immunity limits the therapeutic window for systemic inhibition, suggesting the need for tumor‐specific targeting strategies.	
Targeting noncoding RNAs				
miRNA	Triple‐negative breast cancer	Human cell lines/mouse models: Radiation prevents tumor progression by inhibiting the miR‐93‐5p/EphA4/NF‐κB pathway in triple‐negative breast cancer [398].	Consensus: Noncoding RNAs are key regulators of chromatin states and gene expression.	This reveals novel opportunities for overcoming resistance by combining targeted epigenetic modulators with standard therapies such as radiotherapy.
			Differences: The expression and function of noncoding RNAs are typically highly cell‐type and species‐specific.	
Combination chemotherapy				
PRMT1 inhibitor + gemcitabine	Pancreatic cancer	Human cell lines/mouse models: Pharmacological inhibition of PRMT1 in combination with gemcitabine has a synergistic effect on pancreatic tumor growth in vitro and in vivo [399].	Consensus: Drugs targeting chromatin accessibility can reshape tumor cell states, thereby sensitizing them to conventional chemotherapy.	Support the combination of epigenetic therapies with standard chemotherapy to overcome drug resistance and enhance therapeutic efficacy.
			Discrepancy: The extent of synergistic effects may vary due to tumor heterogeneity and requires validation in models more closely resembling human tumors.	
Combination immunotherapy				
EZH2 inhibitor + immune checkpoint inhibitor (ICI)	Multiple solid tumors	Human clinical trials: EZH2 inhibition enhances therapeutic efficacy by directly acting on CAR‐T cells, thereby improving immunotherapy outcomes in patients with B‐cell lymphoma [400].	Consensus: Altering chromatin states can modulate the tumor immune microenvironment, thereby enhancing the efficacy of immunotherapy.	This underscores the urgent need to develop complex 3D models incorporating immune components—such as organoid‐immune cell coculture systems—to more accurately predict the efficacy of combined immunotherapies.
		Human cell lines/mouse models: EZH2 inhibitors have been demonstrated to enhance CD8+ T cell infiltration and function, producing synergistic antitumor effects when combined with anti‐PD‐1 antibodies [401, 402].	Discrepancies: The mouse immune system differs from that of humans, and current human in vitro models require refinement in immunological coculture approaches.	
		Limitations of 3D bioprinting/PDOs: Current PDOs and bioprinted models typically lack a complete immune microenvironment, restricting their use in simulating interactions with immunotherapy [403].		
Combination targeted therapy				
Targeted drug delivery	Hepatic metastatic colorectal cancer	3D bioprinted models: An advanced 3D bioprinted liver metastasis colorectal cancer (CRC) model demonstrates that oncolytic viruses carrying 5‐fluorouracil prodrugs can specifically target and penetrate CRC tumor regions, achieving targeted chemotherapy effects equivalent to higher doses of systemic administration [404].	Consensus: Virus‐mediated targeted delivery enhances the tumor specificity of epigenetic drugs or chemotherapeutic agents.	Provides robust proof‐of‐concept for virus‐based targeted therapeutic strategies and demonstrates the unique advantages of 3D bioprinted models in evaluating complex therapeutic modalities such as viral delivery
			Distinction: For the first time, 3D bioprinted models provide a visual demonstration of the spatial specificity of this targeted delivery and local activation within human‐derived tissue—an achievement difficult to replicate in conventional mouse models.	
Combination radiotherapy				
BET inhibitor/NF‐κB pathway inhibitor + radiotherapy	Breast cancer	Human cell lines/mouse models: Research indicates that targeting the NF‐κB–MIR155HG axis or BET proteins can reverse radiotherapy resistance in breast cancer stem cells, enhancing radiotherapy efficacy in both in vitro and in vivo models [405].	Consensus: Epigenetic regulators serve as key mediators of radiotherapy resistance.	Demonstrated the clinical potential of drugs targeting chromatin accessibility as radiotherapy sensitizers
			Discrepancy: In vivo studies of radiotherapy response remain highly dependent on models that fully replicate the tumor microenvironment and immune system.	

## 5 Conclusion and Outlook

### 5.1 Summaries and Shortcomings

In this review, we systematically elaborate on the structural basis and dynamic regulatory mechanisms of chromatin accessibility. Existing studies have demonstrated that chromatin accessibility, acting as a “gateway” to epigenetic regulation, is modulated by a variety of epigenetic regulatory mechanisms. Herein, we summarize the mechanisms of DNA methylation, histone modification, ncRNA, chromatin remodeling complexes, transcription factors, and chromatin 3D structure that regulate chromatin accessibility and thus affect tumor cell fate. In addition, this review explores the multidimensional role of chromatin accessibility in tumorigenesis and progression, including tumorigenesis, progression, metabolic reprogramming, angiogenesis, tumor stemness, immunity and microenvironment, and tumor drug resistance. Notably, existing studies are mostly based on traditional tissue‐level high‐throughput sequencing technologies (e.g., ATAC‐seq), which are difficult to portray chromatin accessibility heterogeneity at the single‐cell level in the tumor microenvironment. Although single‐cell multiomics technologies (scATAC‐seq combined with scRNA‐seq) and spatial transcriptomics (e.g., Stereo‐seq) have been studied by some scholars but have not yet been popularized [406, 407, 408], it is difficult to reveal the epigenetic basis of cell‐to‐cell interactions in the tumor microenvironment. The integration of multiomics data can resolve the driving mechanisms of accessibility changes in specific tumor types and reveal cell subpopulation‐specific regulatory networks. In addition, the dynamic regulation mechanism of chromatin accessibility is ambiguous and lacks real‐time tracking tools. A combination of real‐time dynamic monitoring techniques, such as single‐molecule live‐cell RNA imaging using CRISPR–Csm [409] and time‐resolved epigenomics (CUT&Tag time‐series analysis) [410, 411], is needed to reveal the molecular mechanisms at a deeper level.

Moreover, this review provides a comprehensive discussion around chromatin‐accessible tumor markers, epigenetic drug development, targeted therapies and combination strategies. In terms of therapeutic strategies, drugs targeting epigenetic nodes such as DNA methylation (e.g. decitabine) [311], histone modification (e.g. the EZH2 inhibitor Tazemetostat) [255], chromatin remodeling complexes (e.g. the SWI/SNF inhibitor FHD‐286) [335], and ncRNAs (e.g. MALAT1 ASO) [347] and other epigenetic nodes are in clinical or preclinical studies. Combination chemotherapy, ICIs, and other regimens have shown synergistic potential [214, 335, 380]. Due to space constraints, this review does not address the side effects of epigenetic drugs.

However, the efficacy of epigenetic drugs (e.g., DNMT, HDAC inhibitors) is limited by the epigenetic plasticity of tumor cells. Considering that current studies are mostly based on cellular and animal models, we believe that the establishment of an organoid model will be helpful to gain a deeper understanding of the dynamic regulatory mechanisms of chromatin accessibility and to develop precise therapeutic strategies targeting the open regions of chromatin [412, 413]. In addition, although the excellent anticancer efficacy of epigenetic drugs has been demonstrated, the development of highly selective inhibitors, new strategies for drug resistance, and target specificity requires further investigation. Moreover, although some studies have been conducted using combination strategies with different target epigenetic drugs [269, 314], more research is needed to provide theoretical support at the mechanistic level and to provide more optimized combination strategies.

In addition, the current therapeutic hotspots focus on the combination of epigenetic drugs with conventional therapies (chemotherapy, immunotherapy, targeted therapy, and radiotherapy) [378, 382, 383, 384], which can significantly improve the anticancer efficacy through the synergy of multiple mechanisms. Preclinical and early clinical trials have validated its potential, but large‐sample cohort studies are still needed to validate it through both mechanistic studies and clinical trials. Future research should focus on precise combination strategies to advance this field toward clinical translation and promote cancer treatment into the “era of individualized epigenetic precision.”

### 5.2 Challenges and Prospects

As research advances, novel research strategies and techniques are now making more significant breakthroughs as research continues to progress.

Proteolysis‐targeting chimera (PROTAC), which enables functional studies of chromatin regulatory factors by inducing targeted protein degradation, has emerged as a valuable tool and garnered considerable attention from researchers in academia and the medical field [414]. PROTAC, as a pioneering novel therapeutic approach, redefines the principles of traditional drug discovery. It operates in an event‐driven manner, inducing ubiquitin modification for the clearance of pathogenic proteins through transient binding mechanisms based on the ubiquitin–proteasome system [415]. The PROTAC technology has been successfully used for the degradation of many oncogenic targets to overcome drug resistance, such as targeting AR in ENZ‐resistant PC [416], targeting ER in drug‐resistant breast cancer [417], targeting BTK in ibrutinib‐resistant lymphomas [418], and targeting proteins in BET denervation‐resistant PC [419, 420]. Moreover, combination therapy of PROTAC with epigenetic drugs has also shown promise, such as HDAC–PROTAC (SIRT2 degrader), which can provide higher selectivity and specificity while reducing off‐target effects [421]. Moreover, PROTAC technology opens a new chapter in precision therapy by transforming traditionally nondruggable proteins into interventional targets through a “catalytic degradation” mechanism [414].

However, PROTACs face substantial hurdles in becoming a successful drug discovery approach, primarily related to uncertainty in efficacy, technical challenges, and high development costs. Given the relatively small number of clinical candidates available, it remains to be seen whether PROTAC will become a clinically useful anticancer drug, and more in vitro data and pharmacokinetic studies are needed to pave the way for its application in the clinic. Therefore, the great potential of PROTAC technology in cancer therapy remains to be more fully explored and exploited.

In addition, in recent years, CRISPR and CRISPR‐associated proteins have become a revolutionary gene editing tool, and the CRISPR/Cas technology has also demonstrated great potential in tumor therapy by systematically targeting specific gene loci and regulating chromatin accessibility [422]. A team from Zhejiang University developed a cryo‐shock tumor cell delivery CRISPR–Cas system that triggered synthetic lethality and prolonged survival in mice by CDK4 ablation in KRAS‐mutant NSCLC [423]. Several studies have demonstrated that CRISPR/Cas gene editing can be used to overcome drug resistance in a variety of cancers and that different resistance mechanisms can be targeted using this technology [424, 425, 426, 427]. In addition, in vivo genome‐wide CRISPR screening can be used to identify targets that modulate drug resistance or immunotherapy sensitivity in a variety of cancers. using genome‐wide CRISPR screening, Cao's team found that CD28 in cancer cells promotes immune escape by stabilizing *PD‐L1* mRNA, providing a novel target for overcoming anti‐PD‐1 resistance [428]. Belk et al. identified a new target for anti‐PD‐1 resistance through a series of in vitro and in vivo CRISPR–Cas9 screening systems to identify chromatin remodeling factors that limit T‐cell persistence and demonstrate that modulation of epigenetic status improves T‐cell responses in cancer immunotherapy.

But translating CRISPR/Cas to in vivo gene editing poses significant challenges that need to be addressed, including concerns about specificity, safety, and efficient delivery [429]. Based on several studies, Xu et al. suggest that nanotechnology‐based delivery of CRISPR/Cas9 for cancer gene editing and immunotherapy paves the way for its clinical translation, and that nanotechnology‐based delivery of gene editing using CRISPR/Cas9 is a new dawn in the field of cancer therapy [430]. Thus, although still in its infancy, the CRISPR system offers great promise in the discovery of important cancer genes and therapeutic targets and will continue to play an irreplaceable role in the future.

Nowadays, with the generation‐by‐generation development of artificial intelligence, the integration of single‐cell multiomics and artificial intelligence, interdisciplinary fusion, and big data‐driven are becoming increasingly prominent. The scBridge algorithm developed by Sichuan University effectively overcomes the interference of cellular heterogeneity on multiomics analysis by iteratively integrating scRNA‐seq and scATAC‐seq data [406]. The construction of AI prediction platforms, such as the training of deep learning models based on the The Cancer Genome Atlas and ICGC databases (e.g., XGraphCDS, scMultiomeGRN) [431, 432], has enabled the prediction of patient‐specific chromatin accessibility targets and drug sensitivity from genetic pathways, realizing a leap from “static typing” to “dynamic management” in cancer therapy.

PROTAC and CRISPR technologies are driving chromatin accessibility research from descriptive analysis to precision intervention, but their clinical translation is still limited by delivery efficiency, editing accuracy, and multiomics integration capability. In the future, it is necessary to break through the technical bottleneck through interdisciplinary cooperation, to realize the personalized epigenetic therapy of “time‐space‐target,” to promote the chromatin accessibility markers from scientific research to the clinical leap, and ultimately to rewrite the clinical practice of tumor treatment.

## 6 Conclusion

**FIGURE 6:**
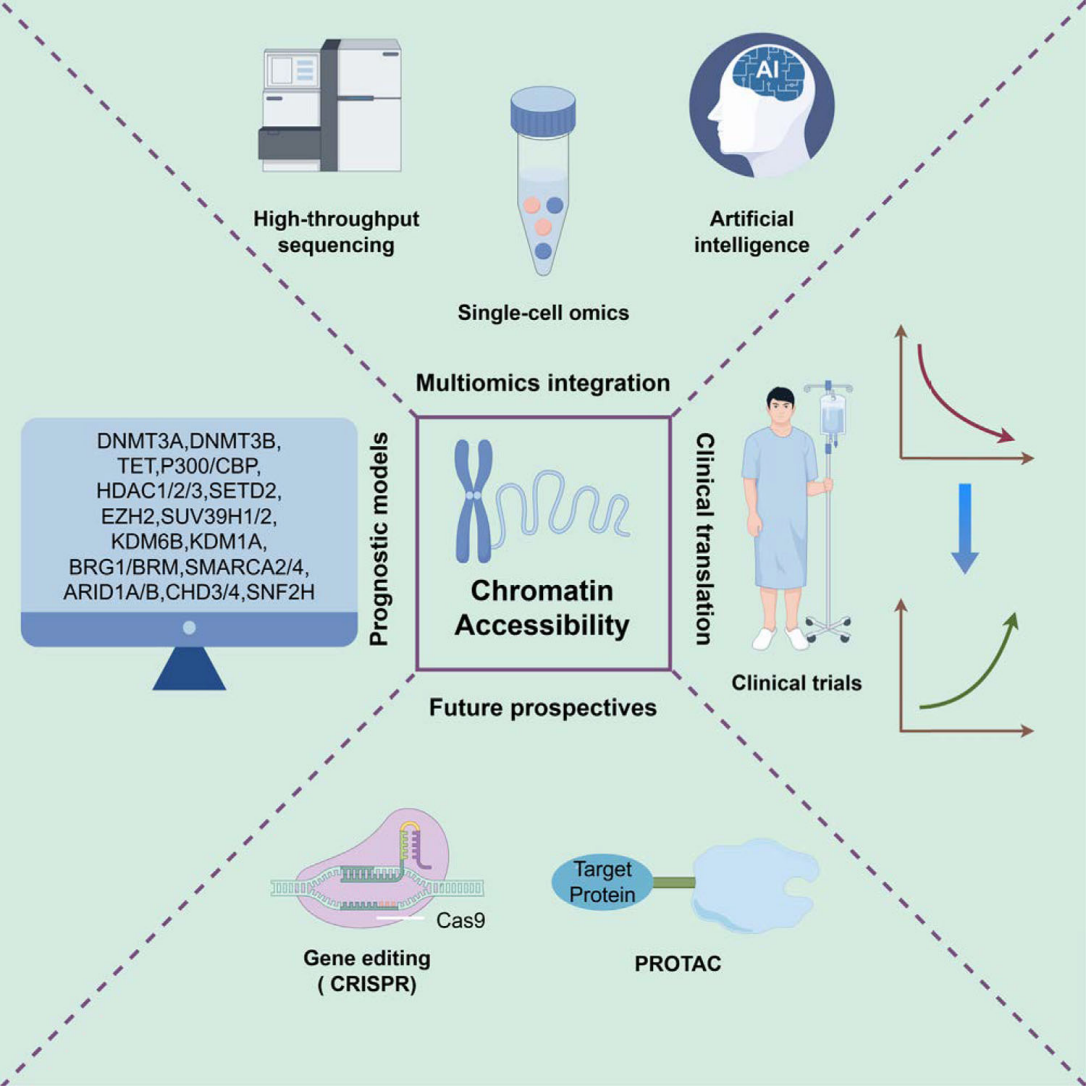
Future perspectives and clinical translation of chromatin accessibility researches. Prognostic modeling based on chromatin accessibility, future perspectives on chromatin accessibility research through multiomics integration and emerging technologies, and its translation into clinical trials and prognostic analysis.

Chromatin accessibility, as a core hub of epigenetic regulation, has been gradually transformed from basic mechanism investigation to clinical application and is moving from static description to dynamic resolution and precise intervention. Despite the challenges of heterogeneity, technical resolution, and therapeutic safety, with the rapid development of single‐cell technology, PROTAC‐targeted therapy, and CRISPR gene editing tools, it is expected to realize a new paradigm of tumor therapy that emphasizes both spatio‐temporal regulation and individualized intervention. However, there are still many challenges in the complexity, technical bottleneck, and clinical translation of tumor epigenetic regulation. Multiomics integration, artificial intelligence, and interdisciplinary innovation will be the key to break through the current bottlenecks, which is expected to reveal the panoramic regulatory network of chromatin accessibility in tumorigenesis and development, and ultimately promote the epigenetic‐based precision tumor therapy to enter into a new stage of comprehensive leap (Figure 6).

## Author Contributions

Wentao Xia, Kun Ding, and Yansu Chen drafted the manuscript and prepared the figures. Min Jiang and Yefei Huang revised the manuscript text. All authors have read and approved the manuscript.

## Funding

This study was supported in part by the National Natural Science Foundation of China (82473588), the 2023 Qing Lan Project (Chen Yansu), the Graduate Research Innovation Plan of Jiangsu Province (KYCX23_2964), the Natural Science Foundation of Jiangsu Province (BK20241038), the Natural Science Fund for Colleges and Universities in Jiangsu Province (24KJD320005), and the General Program of Basic Science (Natural Science) Research for Colleges and Universities in Jiangsu Province (24KJB330010).

## Conflicts of Interest

The authors declare no conflicts of interest.

## Ethics Statement

The authors have nothing to report.

## Data Availability

The authors have nothing to report.

## References

[1] P. A. Jones and S. B. Baylin , “The Epigenomics of Cancer,” Cell 128, no. 4 (2007): 683–692. 17320506 10.1016/j.cell.2007.01.029 PMC3894624

[2] S. Zhou , A. E. Treloar , and M. Lupien , “Emergence of the Noncoding Cancer Genome: A Target of Genetic and Epigenetic Alterations,” Cancer Discovery 6, no. 11 (2016): 1215–1229. 27807102 10.1158/2159-8290.CD-16-0745 PMC5117653

[3] T. Chiba , H. Marusawa , and T. Ushijima , “Inflammation‐associated Cancer Development in Digestive Organs: Mechanisms and Roles for Genetic and Epigenetic Modulation,” Gastroenterology 143, no. 3 (2012): 550–563. 22796521 10.1053/j.gastro.2012.07.009

[4] C. H. Waddington , “The Epigenotype. 1942,” International Journal of Epidemiology 41, no. 1 (2012): 10–13. 22186258 10.1093/ije/dyr184

[5] T. B. Toh , J. J. Lim , and E. K. Chow , “Epigenetics in Cancer Stem Cells,” Molecular Cancer 16, no. 1 (2017): 29. 28148257 10.1186/s12943-017-0596-9 PMC5286794

[6] A. Zhao , H. Zhou , J. Yang , M. Li , and T. Niu , “Epigenetic Regulation in Hematopoiesis and Its Implications in the Targeted Therapy of Hematologic Malignancies,” Signal Transduct Target Ther 8, no. 1 (2023): 71. 36797244 10.1038/s41392-023-01342-6 PMC9935927

[7] V. Davalos and M. Esteller , “Cancer Epigenetics in Clinical Practice,” CA: A Cancer Journal for Clinicians 73, no. 4 (2023): 376–424. 36512337 10.3322/caac.21765

[8] S. L. Klemm , Z. Shipony , and W. J. Greenleaf , “Chromatin Accessibility and the Regulatory Epigenome,” Nature Reviews Genetics 20, no. 4 (2019): 207–220. 10.1038/s41576-018-0089-8 30675018

[9] P. Gujral , V. Mahajan , and A. C. Lissaman , “Ponnampalam AP. Histone Acetylation and the Role of Histone Deacetylases in Normal Cyclic Endometrium,” Reproductive Biology and Endocrinology [Electronic Resource]: RB&E 18, no. 1 (2020): 84. 32791974 10.1186/s12958-020-00637-5 PMC7425564

[10] Z. Zhong , S. Feng , S. H. Duttke , et al., “DNA Methylation‐linked Chromatin Accessibility Affects Genomic Architecture in Arabidopsis,” PNAS 118, no. 5 (2021): e2023347118. 33495321 10.1073/pnas.2023347118 PMC7865151

[11] J. R. Dixon , S. Selvaraj , F. Yue , et al., “Topological Domains in Mammalian Genomes Identified by Analysis of Chromatin Interactions,” Nature 485, no. 7398 (2012): 376–380. 22495300 10.1038/nature11082 PMC3356448

[12] F. C. Grandi , H. Modi , L. Kampman , and M. R. Corces , “Chromatin Accessibility Profiling by ATAC‐seq,” Nature Protocols 17, no. 6 (2022): 1518–1552. 35478247 10.1038/s41596-022-00692-9 PMC9189070

[13] D. Alonso‐Curbelo , Y. J. Ho , C. Burdziak , et al., “A Gene‐environment‐induced Epigenetic Program Initiates Tumorigenesis,” Nature 590, no. 7847 (2021): 642–648. 33536616 10.1038/s41586-020-03147-x PMC8482641

[14] X. Shi , Y. Li , Q. Yuan , et al., “Integrated Profiling of human Pancreatic Cancer Organoids Reveals Chromatin Accessibility Features Associated With Drug Sensitivity,” Nature Communications 13, no. 1 (2022): 2169. 10.1038/s41467-022-29857-6 PMC9023604 35449156

[15] N. J. Lodato , A. Rampersaud , and D. J. Waxman , “Impact of CAR Agonist Ligand TCPOBOP on Mouse Liver Chromatin Accessibility,” Toxicological Sciences 164, no. 1 (2018): 115–128. 29617930 10.1093/toxsci/kfy070 PMC6016691

[16] X. Sun , S. C. Wang , Y. Wei , et al., “Arid1a has Context‐Dependent Oncogenic and Tumor Suppressor Functions in Liver Cancer,” Cancer Cell 32, no. 5 (2017): 574–589. 29136504 10.1016/j.ccell.2017.10.007 PMC5728182

[17] F. Tang , D. Xu , S. Wang , et al., “Chromatin Profiles Classify Castration‐resistant Prostate Cancers Suggesting Therapeutic Targets,” Science 376, no. 6596 (2022): e1505. 10.1126/science.abe1505 PMC9299269 35617398

[18] P. Verma , Y. Zhou , Z. Cao , et al., “ALC1 links Chromatin Accessibility to PARP Inhibitor Response in Homologous Recombination‐deficient Cells,” Nature Cell Biology 23, no. 2 (2021): 160–171. 33462394 10.1038/s41556-020-00624-3 PMC7880902

[19] Y. Xue , J. L. Morris , K. Yang , et al., “SMARCA4/2 loss Inhibits Chemotherapy‐induced Apoptosis by Restricting IP3R3‐mediated Ca(2+) Flux to Mitochondria,” Nature Communications 12, no. 1 (2021): 5404. 10.1038/s41467-021-25260-9 PMC8438089 34518526

[20] A. R. Mansisidor and V. I. Risca , “Chromatin Accessibility: Methods, Mechanisms, and Biological Insights,” Nucleus 13, no. 1 (2022): 236–276. 36404679 10.1080/19491034.2022.2143106 PMC9683059

[21] Y. Chen , R. Liang , Y. Li , et al., “Chromatin Accessibility: Biological Functions, Molecular Mechanisms and Therapeutic Application,” Signal Transduct Target Ther 9, no. 1 (2024): 340. 39627201 10.1038/s41392-024-02030-9 PMC11615378

[22] E. I. Campos and D. Reinberg , “Histones: Annotating Chromatin,” Annual Review of Genetics 43 (2009): 559–599. 10.1146/annurev.genet.032608.103928 19886812

[23] K. Luger , A. W. Mader , R. K. Richmond , D. F. Sargent , and T. J. Richmond , “Crystal Structure of the Nucleosome Core Particle at 2.8 a Resolution,” Nature 389, no. 6648 (1997): 251–260. 9305837 10.1038/38444

[24] N. Kaplan , I. K. Moore , Y. Fondufe‐Mittendorf , et al., “The DNA‐encoded Nucleosome Organization of a Eukaryotic Genome,” Nature 458, no. 7236 (2009): 362–366. 19092803 10.1038/nature07667 PMC2658732

[25] M. De Cecco , S. W. Criscione , E. J. Peckham , et al., “Genomes of Replicatively Senescent Cells Undergo Global Epigenetic Changes Leading to Gene Silencing and Activation of Transposable Elements,” Aging Cell 12, no. 2 (2013): 247–256. 23360310 10.1111/acel.12047 PMC3618682

[26] S. J. Mcbryant , V. H. Adams , and J. C. Hansen , “Chromatin Architectural Proteins,” Chromosome Research 14, no. 1 (2006): 39–51. 16506095 10.1007/s10577-006-1025-x

[27] R. E. Thurman , E. Rynes , R. Humbert , et al., “The Accessible Chromatin Landscape of the human Genome,” Nature 489, no. 7414 (2012): 75–82. 22955617 10.1038/nature11232 PMC3721348

[28] V. W. Zhou , A. Goren , and B. E. Bernstein , “Charting Histone Modifications and the Functional Organization of Mammalian Genomes,” Nature Reviews Genetics 12, no. 1 (2011): 7–18. 10.1038/nrg2905 21116306

[29] M. Okano , D. W. Bell , D. A. Haber , and E. Li , “DNA Methyltransferases Dnmt3a and Dnmt3b Are Essential for De Novo Methylation and Mammalian Development,” Cell 99, no. 3 (1999): 247–257. 10555141 10.1016/s0092-8674(00)81656-6

[30] F. Spada , A. Haemmer , D. Kuch , et al., “DNMT1 but Not Its Interaction With the Replication Machinery Is Required for Maintenance of DNA Methylation in human Cells,” Journal of Cell Biology 176, no. 5 (2007): 565–571. 17312023 10.1083/jcb.200610062 PMC2064015

[31] A. L. Mattei , N. Bailly , and A. Meissner , “DNA Methylation: A Historical Perspective,” Trends in Genetics 38, no. 7 (2022): 676–707. 35504755 10.1016/j.tig.2022.03.010

[32] J. F. Kribelbauer , X. Lu , R. Rohs , R. S. Mann , and H. J. Bussemaker , “Toward a Mechanistic Understanding of DNA Methylation Readout by Transcription Factors,” Journal of Molecular Biology 432, no. 6 (2020): 1801–1815. 31689433 10.1016/j.jmb.2019.10.021 PMC6961349

[33] M. Casado‐Pelaez , A. Bueno‐Costa , and M. Esteller , “Single Cell Cancer Epigenetics,” Trends in cancer 8, no. 10 (2022): 820–838. 35821003 10.1016/j.trecan.2022.06.005

[34] H. Zhang , Z. Lang , and J. Zhu , “Dynamics and Function of DNA Methylation in Plants,” Nature Reviews Molecular Cell Biology 19, no. 8 (2018): 489–506. 29784956 10.1038/s41580-018-0016-z

[35] A. V. Lee , K. A. Nestler , and K. B. Chiappinelli , “Therapeutic Targeting of DNA Methylation Alterations in Cancer,” Pharmacology & Therapeutics 258 (2024): 108640. 38570075 10.1016/j.pharmthera.2024.108640

[36] T. Kameda , M. M. Suzuki , A. Awazu , and Y. Togashi , “Structural Dynamics of DNA Depending on Methylation Pattern,” Physical Review E 103, no. 1‐1 (2021): 12404. 10.1103/PhysRevE.103.012404 33601517

[37] S. Li , Y. Peng , D. Landsman , and A. R. Panchenko , “DNA Methylation Cues in Nucleosome Geometry, Stability and Unwrapping,” Nucleic Acids Research 50, no. 4 (2022): 1864–1874. 35166834 10.1093/nar/gkac097 PMC8881801

[38] D. Buitrago , M. Labrador , J. P. Arcon , et al., “Impact of DNA Methylation on 3D Genome Structure,” Nature Communications 12, no. 1 (2021): 3243. 10.1038/s41467-021-23142-8 PMC8163762 34050148

[39] S. Rao , T. Chiu , J. F. Kribelbauer , R. S. Mann , H. J. Bussemaker , and R. Rohs , “Systematic Prediction of DNA Shape Changes due to CpG Methylation Explains Epigenetic Effects on Protein‐DNA Binding,” Epigenetics & chromatin 11, no. 1 (2018): 6. 29409522 10.1186/s13072-018-0174-4 PMC5800008

[40] J. Y. Lee and T. Lee , “Effects of DNA Methylation on the Structure of Nucleosomes,” Journal of the American Chemical Society 134, no. 1 (2012): 173–175. 22148575 10.1021/ja210273w PMC3257366

[41] Y. Peng , S. Li , A. Onufriev , D. Landsman , and A. R. Panchenko , “Binding of Regulatory Proteins to Nucleosomes Is Modulated by Dynamic Histone Tails,” Nature Communications 12, no. 1 (2021): 5280. 10.1038/s41467-021-25568-6 PMC8421395 34489435

[42] A. D. Klocko , M. Uesaka , T. Ormsby , et al., “Nucleosome Positioning by an Evolutionarily Conserved Chromatin Remodeler Prevents Aberrant DNA Methylation in Neurospora,” Genetics 211, no. 2 (2019): 563–578. 30554169 10.1534/genetics.118.301711 PMC6366918

[43] C. K. Collings and J. N. Anderson , “Links Between DNA Methylation and Nucleosome Occupancy in the human Genome,” Epigenetics & chromatin 10, no. 18 (2017): 18. 28413449 10.1186/s13072-017-0125-5 PMC5387343

[44] R. Nakamura , A. Uno , M. Kumagai , S. Morishita , and H. Takeda , “Hypomethylated Domain‐enriched DNA Motifs Prepattern the Accessible Nucleosome Organization in Teleosts,” Epigenetics & chromatin 10, no. 1 (2017): 44. 28931432 10.1186/s13072-017-0152-2 PMC5607494

[45] C. Lovkvist , K. Sneppen , and J. O. Haerter , “Exploring the Link Between Nucleosome Occupancy and DNA Methylation,” Frontiers in Genetics 8 (2017): 232. 29379519 10.3389/fgene.2017.00232 PMC5771128

[46] C. Lovkvist , I. B. Dodd , K. Sneppen , and J. O. Haerter , “DNA Methylation in human Epigenomes Depends on Local Topology of CpG Sites,” Nucleic Acids Research 44, no. 11 (2016): 5123–5132. 26932361 10.1093/nar/gkw124 PMC4914085

[47] J. Yoo , S. Park , C. Maffeo , T. Ha , and A. Aksimentiev , “DNA Sequence and Methylation Prescribe the inside‐out Conformational Dynamics and Bending Energetics of DNA Minicircles,” Nucleic Acids Research 49, no. 20 (2021): 11459–11475. 34718725 10.1093/nar/gkab967 PMC8599915

[48] A. Osakabe , F. Adachi , Y. Arimura , K. Maehara , Y. Ohkawa , and H. Kurumizaka , “Influence of DNA Methylation on Positioning and DNA Flexibility of Nucleosomes With Pericentric Satellite DNA,” Open Biol 5, no. 10 (2015): 150128. 26446621 10.1098/rsob.150128 PMC4632512

[49] S. A. Wajed , P. W. Laird , and T. R. Demeester , “DNA Methylation: An Alternative Pathway to Cancer,” Annals of Surgery 234, no. 1 (2001): 10–20. 11420478 10.1097/00000658-200107000-00003 PMC1421942

[50] D. Cheishvili , L. Boureau , and M. Szyf , “DNA Demethylation and Invasive Cancer: Implications for Therapeutics,” British Journal of Pharmacology 172, no. 11 (2015): 2705–2715. 25134627 10.1111/bph.12885 PMC4439869

[51] D. Sun , X. Gan , L. Liu , et al., “DNA Hypermethylation Modification Promotes the Development of Hepatocellular Carcinoma by Depressing the Tumor Suppressor Gene ZNF334,” Cell death & disease 13, no. 5 (2022): 446. 35534462 10.1038/s41419-022-04895-6 PMC9085879

[52] K. K. Wong , “DNMT1: A Key Drug Target in Triple‐negative Breast Cancer,” Seminars in Cancer Biology 72 (2021): 198–213. 32461152 10.1016/j.semcancer.2020.05.010

[53] W. Zhang , D. Klinkebiel , C. J. Barger , et al., “Global DNA Hypomethylation in Epithelial Ovarian Cancer: Passive Demethylation and Association With Genomic Instability,” Cancers (Basel) 12, no. 3 (2020): 764. 32213861 10.3390/cancers12030764 PMC7140107

[54] Y. Endo , K. Suzuki , Y. Kimura , et al., “Genome‑Wide DNA Hypomethylation Drives a More Invasive Pancreatic Cancer Phenotype and Has Predictive Occult Distant Metastasis and Prognosis Potential,” International Journal of Oncology 60, no. 6 (2022): 61. 35419613 10.3892/ijo.2022.5351 PMC9015190

[55] F. Na , X. Pan , J. Chen , et al., “KMT2C deficiency Promotes Small Cell Lung Cancer Metastasis Through DNMT3A‐mediated Epigenetic Reprogramming,” Nat Cancer 3, no. 6 (2022): 753–767. 35449309 10.1038/s43018-022-00361-6 PMC9969417

[56] H. Guo , J. A. Vuille , B. S. Wittner , et al., “DNA Hypomethylation Silences Anti‐tumor Immune Genes in Early Prostate Cancer and CTCs,” Cell 186, no. 13 (2023): 2765–2782. 37327786 10.1016/j.cell.2023.05.028 PMC10436379

[57] H. Tian , C. Liu , J. Yu , et al., “PHF14 enhances DNA Methylation of SMAD7 Gene to Promote TGF‐beta‐driven Lung Adenocarcinoma Metastasis,” Cell Discovery 9, no. 1 (2023): 41. 37072414 10.1038/s41421-023-00528-0 PMC10113255

[58] C. C. Wong , J. Xu , X. Bian , et al., “In Colorectal Cancer Cells With Mutant KRAS, SLC25A22‐Mediated Glutaminolysis Reduces DNA Demethylation to Increase WNT Signaling, Stemness, and Drug Resistance,” Gastroenterology 159, no. 6 (2020): 2163–2180. 32814111 10.1053/j.gastro.2020.08.016

[59] J. E. Audia and R. M. Campbell , “Histone Modifications and Cancer,” Cold Spring Harbor perspectives in biology 8, no. 4 (2016): a19521. 10.1101/cshperspect.a019521 PMC4817802 27037415

[60] N. P. Nurse , I. Jimenez‐Useche , I. T. Smith , and C. Yuan , “Clipping of Flexible Tails of Histones H3 and H4 Affects the Structure and Dynamics of the Nucleosome,” Biophysical Journal 104, no. 5 (2013): 1081–1088. 23473491 10.1016/j.bpj.2013.01.019 PMC3870800

[61] M. A. Dawson and T. Kouzarides , “Cancer Epigenetics: From Mechanism to Therapy,” Cell 150, no. 1 (2012): 12–27. 22770212 10.1016/j.cell.2012.06.013

[62] A. J. Bannister and T. Kouzarides , “Regulation of Chromatin by Histone Modifications,” Cell Research 21, no. 3 (2011): 381–395. 21321607 10.1038/cr.2011.22 PMC3193420

[63] G. Millan‐Zambrano , A. Burton , A. J. Bannister , and R. Schneider , “Histone Post‐translational Modifications—cause and Consequence of Genome Function,” Nature Reviews Genetics 23, no. 9 (2022): 563–580. 10.1038/s41576-022-00468-7 35338361

[64] L. Sun , H. Zhang , and P. Gao , “Metabolic Reprogramming and Epigenetic Modifications on the Path to Cancer,” Protein Cell 13, no. 12 (2022): 877–919. 34050894 10.1007/s13238-021-00846-7 PMC9243210

[65] V. Morales and H. Richard‐Foy , “Role of Histone N‐terminal Tails and Their Acetylation in Nucleosome Dynamics,” Molecular and Cellular Biology 20, no. 19 (2000): 7230–7237. 10982840 10.1128/mcb.20.19.7230-7237.2000 PMC86277

[66] P. Tropberger and R. Schneider , “Scratching the (lateral) Surface of Chromatin Regulation by Histone Modifications,” Nature structural & molecular biology 20, no. 6 (2013): 657–661. 10.1038/nsmb.2581 23739170

[67] T. Kouzarides , “SnapShot: Histone‐modifying Enzymes,” Cell 128, no. 4 (2007): 802. 17320515 10.1016/j.cell.2007.02.018

[68] M. Wenes , A. Jaccard , T. Wyss , et al., “The Mitochondrial Pyruvate Carrier Regulates Memory T Cell Differentiation and Antitumor Function,” Cell metabolism 34, no. 5 (2022): 731–746. 35452600 10.1016/j.cmet.2022.03.013 PMC9116152

[69] S. Sheu , S. Upadhyayula , V. Dupuy , et al., “A Serotonergic Axon‐cilium Synapse Drives Nuclear Signaling to Alter Chromatin Accessibility,” Cell 185, no. 18 (2022): 3390–3407. 36055200 10.1016/j.cell.2022.07.026 PMC9789380

[70] W. He , Q. Li , and X. Li , “Acetyl‐CoA Regulates Lipid Metabolism and Histone Acetylation Modification in Cancer,” Biochim Biophys Acta Rev Cancer 1878, no. 1 (2023): 188837. 36403921 10.1016/j.bbcan.2022.188837

[71] P. Miziak , M. Baran , L. Borkiewicz , T. Trombik , and A. Stepulak , “Acetylation of Histone h3 in Cancer Progression and Prognosis,” International Journal of Molecular Sciences 25, no. 20 (2024): 10982. 39456765 10.3390/ijms252010982 PMC11507103

[72] J. C. Black , C. Van Rechem , and J. R. Whetstine , “Histone Lysine Methylation Dynamics: Establishment, Regulation, and Biological Impact,” Molecular Cell 48, no. 4 (2012): 491–507. 23200123 10.1016/j.molcel.2012.11.006 PMC3861058

[73] A. Barski , S. Cuddapah , K. Cui , et al., “High‐resolution Profiling of Histone Methylations in the human Genome,” Cell 129, no. 4 (2007): 823–837. 17512414 10.1016/j.cell.2007.05.009

[74] Y. Li , X. Chen , and C. Lu , “The Interplay Between DNA and Histone Methylation: Molecular Mechanisms and Disease Implications,” Embo Reports 22, no. 5 (2021): e51803. 33844406 10.15252/embr.202051803 PMC8097341

[75] Y. Song , F. Wu , and J. Wu , “Targeting Histone Methylation for Cancer Therapy: Enzymes, Inhibitors, Biological Activity and Perspectives,” Journal of hematology & oncology 9, no. 1 (2016): 49. 27316347 10.1186/s13045-016-0279-9 PMC4912745

[76] X. Wang , H. Zhao , L. Lv , L. Bao , X. Wang , and S. Han , “Prognostic Significance of EZH2 Expression in Non‐Small Cell Lung Cancer: A Meta‐analysis,” Scientific Reports 6, no. 19239 (2016). 10.1038/srep19239 PMC4709684 26754405

[77] N. C. Sutopo , J. H. Kim , and J. Y. Cho , “Role of Histone Methylation in Skin Cancers: Histone Methylation‐modifying Enzymes as a New Class of Targets for Skin Cancer Treatment,” Biochim Biophys Acta Rev Cancer 1878, no. 3 (2023): 188865. 36841366 10.1016/j.bbcan.2023.188865

[78] D. Rossetto , N. Avvakumov , and J. Cote , “Histone Phosphorylation: A Chromatin Modification Involved in Diverse Nuclear Events,” Epigenetics 7, no. 10 (2012): 1098–1108. 22948226 10.4161/epi.21975 PMC3469451

[79] A. Sawicka and C. Seiser , “Histone H3 Phosphorylation—a Versatile Chromatin Modification for Different Occasions,” Biochimie 94, no. 11 (2012): 2193–2201. 22564826 10.1016/j.biochi.2012.04.018 PMC3480636

[80] D. Komar and P. Juszczynski , “Rebelled Epigenome: Histone H3S10 Phosphorylation and H3S10 Kinases in Cancer Biology and Therapy,” Clin Epigenetics 12, no. 1 (2020): 147. 33054831 10.1186/s13148-020-00941-2 PMC7556946

[81] Y. Geffen , S. Anand , Y. Akiyama , et al., “Pan‐cancer Analysis of Post‐translational Modifications Reveals Shared Patterns of Protein Regulation,” Cell 186, no. 18 (2023): 3945–3967. 37582358 10.1016/j.cell.2023.07.013 PMC10680287

[82] H. van Attikum and S. M. Gasser , “Crosstalk Between Histone Modifications During the DNA Damage Response,” Trends in Cell Biology 19, no. 5 (2009): 207–217. 19342239 10.1016/j.tcb.2009.03.001

[83] B. Artegiani , L. van Voorthuijsen , R. G. H. Lindeboom , et al., “Probing the Tumor Suppressor Function of BAP1 in CRISPR‐Engineered human Liver Organoids,” Cell Stem Cell 24, no. 6 (2019): 927–943. 31130514 10.1016/j.stem.2019.04.017

[84] F. Merkuri , M. Rothstein , and M. Simoes‐Costa , “Histone Lactylation Couples Cellular Metabolism With Developmental Gene Regulatory Networks,” Nature Communications 15, no. 1 (2024): 90. 10.1038/s41467-023-44121-1 PMC10762033 38167340

[85] Y. Xu , W. Meng , Y. Dai , et al., “Anaerobic Metabolism Promotes Breast Cancer Survival via Histone‐3 Lysine‐18 Lactylation Mediating PPARD Axis,” Cell Death Discov 11, no. 1 (2025): 54. 39922804 10.1038/s41420-025-02334-x PMC11807217

[86] D. Wang , G. Du , X. Chen , et al., “Zeb1‐controlled Metabolic Plasticity Enables Remodeling of Chromatin Accessibility in the Development of Neuroendocrine Prostate Cancer,” Cell Death and Differentiation 31, no. 6 (2024): 779–791. 38654072 10.1038/s41418-024-01295-5 PMC11164927

[87] Y. Jing , D. Ding , G. Tian , et al., “Semisynthesis of Site‐specifically Succinylated Histone Reveals That Succinylation Regulates Nucleosome Unwrapping Rate and DNA Accessibility,” Nucleic Acids Research 48, no. 17 (2020): 9538–9549. 32766790 10.1093/nar/gkaa663 PMC7515725

[88] N. Kamo , T. Kujirai , H. Kurumizaka , H. Murakami , G. Hayashi , and A. Okamoto , “Organoruthenium‐catalyzed Chemical Protein Synthesis to Elucidate the Functions of Epigenetic Modifications on Heterochromatin Factors,” Chemical Science 12, no. 16 (2021): 5926–5937. 35342540 10.1039/d1sc00731a PMC8872386

[89] R. Smith , S. Zentout , M. Rother , et al., “HPF1‐dependent Histone ADP‐ribosylation Triggers Chromatin Relaxation to Promote the Recruitment of Repair Factors at Sites of DNA Damage,” Nature structural & molecular biology 30, no. 5 (2023): 678–691. 10.1038/s41594-023-00977-x 37106138

[90] R. Martinez‐Zamudio and H. C. Ha , “Histone ADP‐ribosylation Facilitates Gene Transcription by Directly Remodeling Nucleosomes,” Molecular and Cellular Biology 32, no. 13 (2012): 2490–2502. 22547677 10.1128/MCB.06667-11 PMC3434492

[91] B. J. Lukasak , M. M. Mitchener , L. Kong , et al., “TGM2‐mediated Histone Transglutamination Is Dictated by Steric Accessibility,” PNAS 119, no. 43 (2022): e2086295177. 10.1073/pnas.2208672119 PMC9618071 36256821

[92] F. Rossin , F. Ciccosanti , M. D'Eletto , L. Occhigrossi , G. M. Fimia , and M. Piacentini , “Type 2 Transglutaminase in the Nucleus: The New Epigenetic Face of a Cytoplasmic Enzyme,” Cellular and Molecular Life Sciences 80, no. 2 (2023): 52. 36695883 10.1007/s00018-023-04698-8 PMC9874183

[93] S. C. Lee , D. W. Adams , J. J. Ipsaro , et al., “Chromatin Remodeling of Histone H3 Variants by DDM1 Underlies Epigenetic Inheritance of DNA Methylation,” Cell 186, no. 19 (2023): 4100–4116. 37643610 10.1016/j.cell.2023.08.001 PMC10529913

[94] E. R. Feierman , S. Louzon , N. A. Prescott , et al., “Histone Variant H2BE Enhances Chromatin Accessibility in Neurons to Promote Synaptic Gene Expression and Long‐term Memory,” Molecular Cell 84, no. 15 (2024): 2822–2837. 39025074 10.1016/j.molcel.2024.06.025 PMC11316635

[95] A. Nikolic , F. Maule , A. Bobyn , et al., “MacroH2A2 antagonizes Epigenetic Programs of Stemness in Glioblastoma,” Nature Communications 14, no. 1 (2023): 3062. 10.1038/s41467-023-38919-2 PMC10224928 37244935

[96] D. Filipescu , S. Carcamo , A. Agarwal , et al., “MacroH2A restricts Inflammatory Gene Expression in Melanoma Cancer‐associated Fibroblasts by Coordinating Chromatin Looping,” Nature Cell Biology 25, no. 9 (2023): 1332–1345. 37605008 10.1038/s41556-023-01208-7 PMC10495263

[97] J. S. Mattick , P. P. Amaral , P. Carninci , et al., “Long Non‐coding RNAs: Definitions, Functions, Challenges and Recommendations,” Nature Reviews Molecular Cell Biology 24, no. 6 (2023): 430–447. 36596869 10.1038/s41580-022-00566-8 PMC10213152

[98] J. Beermann , M. Piccoli , J. Viereck , and T. Thum , “Non‐coding RNAs in Development and Disease: Background, Mechanisms, and Therapeutic Approaches,” Physiological Reviews 96, no. 4 (2016): 1297–1325. 27535639 10.1152/physrev.00041.2015

[99] M. A. Diamantopoulos , P. Tsiakanikas , and A. Scorilas , “Non‐coding RNAs: The Riddle of the Transcriptome and Their Perspectives in Cancer,” Annals of translational medicine 6, no. 12 (2018): 241. 30069443 10.21037/atm.2018.06.10 PMC6046292

[100] J. Wei , K. Huang , C. Yang , and C. Kang , “Non‐coding RNAs as Regulators in Epigenetics (Review),” Oncology Reports 37, no. 1 (2017): 3–9. 27841002 10.3892/or.2016.5236

[101] A. Luts and F. Sundler , “Peptide‐containing Nerve Fibers in the respiratory Tract of the Ferret,” Cell and Tissue Research 258, no. 2 (1989): 259–267. 2582477 10.1007/BF00239446

[102] D. P. Bartel , “Metazoan MicroRNAs,” Cell 173, no. 1 (2018): 20–51. 29570994 10.1016/j.cell.2018.03.006 PMC6091663

[103] B. Shui , T. S. Beyett , Z. Chen , et al., “Oncogenic K‐Ras Suppresses Global miRNA Function,” Molecular Cell 83, no. 14 (2023): 2509–2523. 37402366 10.1016/j.molcel.2023.06.008 PMC10527862

[104] W. Lv , W. Jiang , H. Luo , et al., “Long Noncoding RNA lncMREF Promotes Myogenic Differentiation and Muscle Regeneration by Interacting With the Smarca5/p300 Complex,” Nucleic Acids Research 50, no. 18 (2022): 10733–10755. 36200826 10.1093/nar/gkac854 PMC9561262

[105] E. Martinez‐Terroba , L. M. Plasek‐Hegde , I. Chiotakakos , et al., “Overexpression of Malat1 Drives Metastasis Through Inflammatory Reprogramming of the Tumor Microenvironment,” Science Immunology 9, no. 96 (2024): h5462. 10.1126/sciimmunol.adh5462 PMC12087577 38875320

[106] S. Deng , J. Zhang , J. Su , et al., “RNA M(6)A Regulates Transcription via DNA Demethylation and Chromatin Accessibility,” Nature Genetics 54, no. 9 (2022): 1427–1437. 36071173 10.1038/s41588-022-01173-1

[107] R. Li , H. Zhao , X. Huang , et al., “Super‐enhancer RNA M(6)A Promotes Local Chromatin Accessibility and Oncogene Transcription in Pancreatic Ductal Adenocarcinoma,” Nature Genetics 55, no. 12 (2023): 2224–2234. 37957340 10.1038/s41588-023-01568-8

[108] S. Deng , J. Zhang , J. Su , et al., “RNA M(6)A Regulates Transcription via DNA Demethylation and Chromatin Accessibility,” Nature Genetics 54, no. 9 (2022): 1427–1437. 36071173 10.1038/s41588-022-01173-1

[109] R. Li , H. Zhao , X. Huang , et al., “Super‐enhancer RNA M(6)A Promotes Local Chromatin Accessibility and Oncogene Transcription in Pancreatic Ductal Adenocarcinoma,” Nature Genetics 55, no. 12 (2023): 2224–2234. 37957340 10.1038/s41588-023-01568-8

[110] B. W. L. Lee , Y. H. Chuah , J. Yoon , et al., “METTL8 links mt‐tRNA M(3)C Modification to the HIF1α/RTK/Akt Axis to Sustain GBM Stemness and Tumorigenicity,” Cell death & disease 15, no. 5 (2024): 338. 38744809 10.1038/s41419-024-06718-2 PMC11093979

[111] N. Mashtalir , H. Suzuki , D. P. Farrell , et al., “A Structural Model of the Endogenous human BAF Complex Informs Disease Mechanisms,” Cell 183, no. 3 (2020): 802–817. 33053319 10.1016/j.cell.2020.09.051 PMC7717177

[112] J. Yuan , K. Chen , W. Zhang , and Z. Chen , “Structure of human Chromatin‐remodelling PBAF Complex Bound to a Nucleosome,” Nature 605, no. 7908 (2022): 166–171. 35477757 10.1038/s41586-022-04658-5

[113] A. A. Reyes , R. D. Marcum , and Y. He , “Structure and Function of Chromatin Remodelers,” Journal of Molecular Biology 433, no. 14 (2021): 166929. 33711345 10.1016/j.jmb.2021.166929 PMC8184634

[114] C. Kadoch , D. C. Hargreaves , C. Hodges , et al., “Proteomic and Bioinformatic Analysis of Mammalian SWI/SNF Complexes Identifies Extensive Roles in human Malignancy,” Nature Genetics 45, no. 6 (2013): 592–601. 23644491 10.1038/ng.2628 PMC3667980

[115] Y. Han , A. A. Reyes , S. Malik , and Y. He , “Cryo‐EM Structure of SWI/SNF Complex Bound to a Nucleosome,” Nature 579, no. 7799 (2020): 452–455. 32188938 10.1038/s41586-020-2087-1 PMC7319049

[116] K. Ahmad , S. Brahma , and S. Henikoff , “Epigenetic Pioneering by SWI/SNF family Remodelers,” Molecular Cell 84, no. 2 (2024): 194–201. 38016477 10.1016/j.molcel.2023.10.045 PMC10842064

[117] T. Owen‐Hughes , R. T. Utley , J. Cote , C. L. Peterson , and J. L. Workman , “Persistent Site‐specific Remodeling of a Nucleosome Array by Transient Action of the SWI/SNF Complex,” Science 273, no. 5274 (1996): 513–516. 8662543 10.1126/science.273.5274.513

[118] C. Hodges , J. G. Kirkland , and G. R. Crabtree , “The Many Roles of BAF (mSWI/SNF) and PBAF Complexes in Cancer,” Cold Spring Harbor perspectives in medicine 6, no. 8 (2016): a026930. 27413115 10.1101/cshperspect.a026930 PMC4968166

[119] C. Kadoch and G. R. Crabtree , “Mammalian SWI/SNF Chromatin Remodeling Complexes and Cancer: Mechanistic Insights Gained From human Genomics,” Science Advances 1, no. 5 (2015): e1500447. 26601204 10.1126/sciadv.1500447 PMC4640607

[120] M. H. Bailey , C. Tokheim , E. Porta‐Pardo , et al., “Comprehensive Characterization of Cancer Driver Genes and Mutations,” Cell 173, no. 2 (2018): 371–385. 29625053 10.1016/j.cell.2018.02.060 PMC6029450

[121] I. Versteege , N. Sevenet , J. Lange , et al., “Truncating Mutations of hSNF5/INI1 in Aggressive Paediatric Cancer,” Nature 394, no. 6689 (1998): 203–206. 9671307 10.1038/28212

[122] R. Murakami , N. Matsumura , J. B. Brown , et al., “Exome Sequencing Landscape Analysis in Ovarian Clear Cell Carcinoma Shed Light on Key Chromosomal Regions and Mutation Gene Networks,” American Journal of Pathology 187, no. 10 (2017): 2246–2258. 28888422 10.1016/j.ajpath.2017.06.012

[123] S. Asaka , Y. Liu , Z. Yu , et al., “ARID1A regulates Progesterone Receptor Expression in Early Endometrial Endometrioid Carcinoma Pathogenesis,” Modern Pathology 36, no. 2 (2023): 100045. 36853791 10.1016/j.modpat.2022.100045

[124] G. Angelico , G. Attanasio , L. Colarossi , et al., “ARID1A mutations in Gastric Cancer: A Review With Focus on Clinicopathological Features, Molecular Background and Diagnostic Interpretation,” Cancers (Basel) 16, no. 11 (2024): 2062. 38893181 10.3390/cancers16112062 PMC11171396

[125] K. C. Helming , X. Wang , and C. W. M. Roberts , “Vulnerabilities of Mutant SWI/SNF Complexes in Cancer,” Cancer Cell 26, no. 3 (2014): 309–317. 25203320 10.1016/j.ccr.2014.07.018 PMC4159614

[126] C. P. Concepcion , S. Ma , L. M. Lafave , et al., “Smarca4 inactivation Promotes Lineage‐Specific Transformation and Early Metastatic Features in the Lung,” Cancer discovery 12, no. 2 (2022): 562–585. 34561242 10.1158/2159-8290.CD-21-0248 PMC8831463

[127] Y. Ding , W. Wang , D. Ma , et al., “Smarca5‐mediated Epigenetic Programming Facilitates Fetal HSPC Development in Vertebrates,” Blood 137, no. 2 (2021): 190–202. 32756943 10.1182/blood.2020005219 PMC7820875

[128] A. Flaus and T. Owen‐Hughes , “Mechanisms for ATP‐dependent Chromatin Remodelling: The Means to the End,” Febs Journal 278, no. 19 (2011): 3579–3595. 21810178 10.1111/j.1742-4658.2011.08281.x PMC4162296

[129] D. Barisic , M. B. Stadler , M. Iurlaro , and D. Schubeler , “Mammalian ISWI and SWI/SNF Selectively Mediate Binding of Distinct Transcription Factors,” Nature 569, no. 7754 (2019): 136–140. 30996347 10.1038/s41586-019-1115-5 PMC6522387

[130] M. L. Bomber , J. Wang , Q. Liu , et al., “Human SMARCA5 Is Continuously Required to Maintain Nucleosome Spacing,” Molecular Cell 83, no. 4 (2023): 507–522. 36630954 10.1016/j.molcel.2022.12.018 PMC9974918

[131] C. R. Clapier , J. Iwasa , B. R. Cairns , and C. L. Peterson , “Mechanisms of Action and Regulation of ATP‐dependent Chromatin‐remodelling Complexes,” Nature Reviews Molecular Cell Biology 18, no. 7 (2017): 407–422. 28512350 10.1038/nrm.2017.26 PMC8127953

[132] H. T. Dao , B. E. Dul , G. P. Dann , G. P. Liszczak , and T. W. Muir , “A Basic Motif Anchoring ISWI to Nucleosome Acidic Patch Regulates Nucleosome Spacing,” Nature Chemical Biology 16, no. 2 (2020): 134–142. 31819269 10.1038/s41589-019-0413-4 PMC6982587

[133] M. Iurlaro , F. Masoni , I. M. Flyamer , et al., “Systematic Assessment of ISWI Subunits Shows That NURF Creates Local Accessibility for CTCF,” Nature Genetics 56, no. 6 (2024): 1203–1212. 38816647 10.1038/s41588-024-01767-x PMC11176080

[134] Y. Li , H. Gong , P. Wang , et al., “The Emerging Role of ISWI Chromatin Remodeling Complexes in Cancer,” Journal of Experimental & Clinical Cancer Research 40, no. 1 (2021): 346. 34736517 10.1186/s13046-021-02151-x PMC8567610

[135] Y. Buganim , I. Goldstein , D. Lipson , et al., “A Novel Translocation Breakpoint Within the BPTF Gene Is Associated With a Pre‐malignant Phenotype,” PLoS ONE 5, no. 3 (2010): e9657. 20300178 10.1371/journal.pone.0009657 PMC2836376

[136] H. Itamochi , T. Oishi , N. Oumi , et al., “Whole‐genome Sequencing Revealed Novel Prognostic Biomarkers and Promising Targets for Therapy of Ovarian Clear Cell Carcinoma,” British Journal of Cancer 117, no. 5 (2017): 717–724. 28728166 10.1038/bjc.2017.228 PMC5572180

[137] J. Perez‐Pena , R. Paez , C. Nieto‐Jimenez , et al., “Mapping Bromodomains in Breast Cancer and Association With Clinical Outcome,” Scientific Reports 9, no. 1 (2019): 5734. 30952871 10.1038/s41598-019-41934-3 PMC6450889

[138] K. Pietrzak , R. Kuzyakiv , R. Simon , et al., “TIP5 primes Prostate Luminal Cells for the Oncogenic Transformation Mediated by PTEN‐loss,” PNAS 117, no. 7 (2020): 3637–3647. 32024754 10.1073/pnas.1911673117 PMC7035629

[139] M. Dai , S. Hu , C. Liu , et al., “BPTF Cooperates With p50 NF‐kappaB to Promote COX‐2 Expression and Tumor Cell Growth in Lung Cancer,” American journal of translational research 11, no. 12 (2019): 7398–7409. 31934287 PMC6943470

[140] T. Muhammad , S. F. Pastore , K. Good , J. Ausio , and J. B. Vincent , “Chromatin Gatekeeper and Modifier CHD Proteins in Development, and in Autism and Other Neurological Disorders,” Psychiatric Genetics 33, no. 6 (2023): 213–232. 37851134 10.1097/YPG.0000000000000353

[141] J. F. Flanagan , L. Mi , M. Chruszcz , et al., “Double Chromodomains Cooperate to Recognize the Methylated Histone H3 Tail,” Nature 438, no. 7071 (2005): 1181–1185. 16372014 10.1038/nature04290

[142] H. F. Allen , P. A. Wade , and T. G. Kutateladze , “The NuRD Architecture,” Cellular and Molecular Life Sciences 70, no. 19 (2013): 3513–3524. 23340908 10.1007/s00018-012-1256-2 PMC3652912

[143] L. Lyn‐Cook , B. Word , N. George , B. Lyn‐Cook , and G. Hammons , “Effect of Cigarette Smoke Condensate on Gene Promoter Methylation in human Lung Cells,” Tob Induc Dis 12, no. 1 (2014): 15. 25214829 10.1186/1617-9625-12-15 PMC4160916

[144] P. Dey , M. P. Ponnusamy , S. Deb , and S. K. Batra , “Human RNA Polymerase II‐association Factor 1 (hPaf1/PD2) Regulates Histone Methylation and Chromatin Remodeling in Pancreatic Cancer,” PLoS ONE 6, no. 10 (2011): e26926. 22046413 10.1371/journal.pone.0026926 PMC3203178

[145] M. Fatemi , T. A. Paul , G. M. Brodeur , B. Shokrani , H. Brim , and H. Ashktorab , “Epigenetic Silencing of CHD5, a Novel Tumor‐suppressor Gene, Occurs in Early Colorectal Cancer Stages,” Cancer 120, no. 2 (2014): 172–180. 24243398 10.1002/cncr.28316 PMC3947327

[146] R. Fueyo , S. Iacobucci , S. Pappa , et al., “Lineage Specific Transcription Factors and Epigenetic Regulators Mediate TGFbeta‐dependent Enhancer Activation,” Nucleic Acids Research 46, no. 7 (2018): 3351–3365. 29438503 10.1093/nar/gky093 PMC5909450

[147] S. Badodi , A. Dubuc , X. Zhang , et al., “Convergence of BMI1 and CHD7 on ERK Signaling in Medulloblastoma,” Cell reports 21, no. 10 (2017): 2772–2784. 29212025 10.1016/j.celrep.2017.11.021 PMC5732319

[148] A. J. Morrison and X. Shen , “Chromatin Remodelling Beyond Transcription: The INO80 and SWR1 Complexes,” Nature Reviews Molecular Cell Biology 10, no. 6 (2009): 373–384. 19424290 10.1038/nrm2693 PMC6103619

[149] Z. Ren , W. Zhao , D. Li , et al., “INO80‐Dependent Remodeling of Transcriptional Regulatory Network Underlies the Progression of Heart Failure,” Circulation 149, no. 14 (2024): 1121–1138. 38152931 10.1161/CIRCULATIONAHA.123.065440

[150] A. Lafon , S. Taranum , F. Pietrocola , et al., “INO80 chromatin Remodeler Facilitates Release of RNA Polymerase II From chromatin for Ubiquitin‐Mediated Proteasomal Degradation,” Molecular Cell 60, no. 5 (2015): 784–796. 26656161 10.1016/j.molcel.2015.10.028 PMC4760348

[151] G. J. Gowans , A. N. Schep , K. M. Wong , D. A. King , W. J. Greenleaf , and A. J. Morrison , “INO80 chromatin Remodeling Coordinates Metabolic Homeostasis With Cell Division,” Cell reports 22, no. 3 (2018): 611–623. 29346761 10.1016/j.celrep.2017.12.079 PMC5949282

[152] P. Chakraborty and T. Magnuson , “INO80 regulates Chromatin Accessibility to Facilitate Suppression of Sex‐linked Gene Expression During Mouse Spermatogenesis,” PLos Genet 20, no. 10 (2024): e1011431. 39405305 10.1371/journal.pgen.1011431 PMC11508167

[153] S. Hur , E. Park , J. Han , et al., “Roles of human INO80 Chromatin Remodeling Enzyme in DNA Replication and Chromosome Segregation Suppress Genome Instability,” Cellular and Molecular Life Sciences 67, no. 13 (2010): 2283–2296. 20237820 10.1007/s00018-010-0337-3 PMC11115786

[154] M. Papamichos‐Chronakis , S. Watanabe , O. J. Rando , and C. L. Peterson , “Global Regulation of H2A.Z Localization by the INO80 Chromatin‐remodeling Enzyme Is Essential for Genome Integrity,” Cell 144, no. 2 (2011): 200–213. 21241891 10.1016/j.cell.2010.12.021 PMC3035940

[155] S. Negrini , V. G. Gorgoulis , and T. D. Halazonetis , “Genomic Instability–an Evolving Hallmark of Cancer,” Nature Reviews Molecular Cell Biology 11, no. 3 (2010): 220–228. 20177397 10.1038/nrm2858

[156] S. Lee , H. Lee , S. Hur , et al., “INO80 haploinsufficiency Inhibits Colon Cancer Tumorigenesis via Replication Stress‐induced Apoptosis,” Oncotarget 8, no. 70 (2017): 115041–115053. 29383140 10.18632/oncotarget.22984 PMC5777752

[157] J. A. Belk , W. Yao , N. Ly , et al., “Genome‐wide CRISPR Screens of T Cell Exhaustion Identify Chromatin Remodeling Factors That Limit T Cell Persistence,” Cancer Cell 40, no. 7 (2022): 768–786. 35750052 10.1016/j.ccell.2022.06.001 PMC9949532

[158] L. Prendergast , U. L. Mcclurg , R. Hristova , et al., “Resolution of R‐loops by INO80 Promotes DNA Replication and Maintains Cancer Cell Proliferation and Viability,” Nature Communications 11, no. 1 (2020): 4534. 10.1038/s41467-020-18306-x PMC7484789 32913330

[159] F. Spitz and E. E. M. Furlong , “Transcription Factors: From Enhancer Binding to Developmental Control,” Nature Reviews Genetics 13, no. 9 (2012): 613–626. 10.1038/nrg3207 22868264

[160] P. Weidemuller , M. Kholmatov , E. Petsalaki , and J. B. Zaugg , “Transcription Factors: Bridge Between Cell Signaling and Gene Regulation,” Proteomics 21, no. 23‐24 (2021): e2000034. 34314098 10.1002/pmic.202000034

[161] L. A. Mirny , “Nucleosome‐mediated Cooperativity Between Transcription Factors,” PNAS 107, no. 52 (2010): 22534–22539. 21149679 10.1073/pnas.0913805107 PMC3012490

[162] K. J. Brennan , M. Weilert , S. Krueger , et al., “Chromatin Accessibility in the Drosophila Embryo Is Determined by Transcription Factor Pioneering and Enhancer Activation,” Developmental Cell 58, no. 19 (2023): 1898–1916. 37557175 10.1016/j.devcel.2023.07.007 PMC10592203

[163] D. Benveniste , H. Sonntag , G. Sanguinetti , and D. Sproul , “Transcription Factor Binding Predicts Histone Modifications in human Cell Lines,” PNAS 111, no. 37 (2014): 13367–13372. 25187560 10.1073/pnas.1412081111 PMC4169916

[164] Y. Muto , P. C. Wilson , N. Ledru , et al., “Single Cell Transcriptional and Chromatin Accessibility Profiling Redefine Cellular Heterogeneity in the Adult human Kidney,” Nature Communications 12, no. 1 (2021): 2190. 10.1038/s41467-021-22368-w PMC8044133 33850129

[165] T. J. Hansen and E. Hodges , “ATAC‐STARR‐seq Reveals Transcription Factor‐bound Activators and Silencers Within Chromatin‐accessible Regions of the human Genome,” Genome Research 32, no. 8 (2022): 1529–1541. 35858748 10.1101/gr.276766.122 PMC9435738

[166] Y. Wang , Y. Yu , L. Li , et al., “Bile Acid‐dependent Transcription Factors and Chromatin Accessibility Determine Regional Heterogeneity of Intestinal Antimicrobial Peptides,” Nature Communications 14, no. 1 (2023): 5093. 10.1038/s41467-023-40565-7 PMC10444805 37607912

[167] Z. Liu , Y. Hu , H. Xie , et al., “Single‐Cell Chromatin Accessibility Analysis Reveals the Epigenetic Basis and Signature Transcription Factors for the Molecular Subtypes of Colorectal Cancers,” Cancer discovery 14, no. 6 (2024): 1082–1105. 38445965 10.1158/2159-8290.CD-23-1445

[168] J. Chen , I. F. Lopez‐Moyado , H. Seo , et al., “NR4A transcription Factors Limit CAR T Cell Function in Solid Tumours,” Nature 567, no. 7749 (2019): 530–534. 30814732 10.1038/s41586-019-0985-x PMC6546093

[169] L. Helminen , J. Huttunen , M. Tulonen , et al., “Chromatin Accessibility and Pioneer Factor FOXA1 Restrict Glucocorticoid Receptor Action in Prostate Cancer,” Nucleic Acids Research 52, no. 2 (2024): 625–642. 38015476 10.1093/nar/gkad1126 PMC10810216

[170] J. Dekker and L. Mirny , “The 3D Genome as Moderator of Chromosomal Communication,” Cell 164, no. 6 (2016): 1110–1121. 26967279 10.1016/j.cell.2016.02.007 PMC4788811

[171] I. Y. Quiroga , J. H. Ahn , G. G. Wang , and D. Phanstiel , “Oncogenic Fusion Proteins and Their Role in Three‐dimensional Chromatin Structure, Phase Separation, and Cancer,” Current opinion in genetics & development 74, no. 101901 (2022): 101901. 35427897 10.1016/j.gde.2022.101901 PMC9156545

[172] N. J. Kirkland , S. H. Skalak , A. J. Whitehead , et al., “Age‐dependent Lamin Changes Induce Cardiac Dysfunction via Dysregulation of Cardiac Transcriptional Programs,” Nat Aging 3, no. 1 (2023): 17–33. 36845078 10.1038/s43587-022-00323-8 PMC9956937

[173] N. G. Bediaga , H. D. Coughlan , T. M. Johanson , et al., “Multi‐level Remodelling of Chromatin Underlying Activation of human T Cells,” Scientific Reports 11, no. 1 (2021): 528. 33436846 10.1038/s41598-020-80165-9 PMC7804404

[174] S. S. P. Rao , M. H. Huntley , N. C. Durand , et al., “A 3D Map of the human Genome at Kilobase Resolution Reveals Principles of Chromatin Looping,” Cell 159, no. 7 (2014): 1665–1680. 25497547 10.1016/j.cell.2014.11.021 PMC5635824

[175] V. Ramani , J. Shendure , and Z. Duan , “Understanding Spatial Genome Organization: Methods and Insights,” Genomics, Proteomics & Bioinformatics 14, no. 1 (2016): 7–20. 10.1016/j.gpb.2016.01.002 PMC4792841 26876719

[176] Q. Szabo , F. Bantignies , and G. Cavalli , “Principles of Genome Folding Into Topologically Associating Domains,” Science Advances 5, no. 4 (2019): w1668. 10.1126/sciadv.aaw1668 PMC6457944 30989119

[177] T. Waldman , “Emerging Themes in Cohesin Cancer Biology,” Nature Reviews Cancer 20, no. 9 (2020): 504–515. 32514055 10.1038/s41568-020-0270-1

[178] K. C. Akdemir , V. T. Le , S. Chandran , et al., “Disruption of Chromatin Folding Domains by Somatic Genomic Rearrangements in human Cancer,” Nature Genetics 52, no. 3 (2020): 294–305. 32024999 10.1038/s41588-019-0564-y PMC7058537

[179] P. Sui , Z. Wang , P. Zhang , and F. Pan , “Three‐dimensional Chromatin Landscapes in MLLr AML,” Exp Hematol Oncol 13, no. 1 (2024): 56. 38778427 10.1186/s40164-024-00523-5 PMC11110396

[180] H. Luo , G. Zhu , M. A. Eshelman , et al., “HOTTIP‐dependent R‐loop Formation Regulates CTCF Boundary Activity and TAD Integrity in Leukemia,” Molecular Cell 82, no. 4 (2022): 833–851. 35180428 10.1016/j.molcel.2022.01.014 PMC8985430

[181] Q. Lai , K. Hamamoto , H. Luo , et al., “NPM1 mutation Reprograms Leukemic Transcription Network via Reshaping TAD Topology,” Leukemia 37, no. 8 (2023): 1732–1736. 37365294 10.1038/s41375-023-01942-9 PMC10400418

[182] R. San Martin , P. Das , R. Dos Reis Marques , et al., “Chromosome Compartmentalization Alterations in Prostate Cancer Cell Lines Model Disease Progression,” Journal of Cell Biology 221, no. 2 (2022): e202104108. 34889941 10.1083/jcb.202104108 PMC8669499

[183] B. Ren , J. Yang , C. Wang , et al., “High‐resolution Hi‐C Maps Highlight Multiscale 3D Epigenome Reprogramming During Pancreatic Cancer Metastasis,” Journal of hematology & oncology 14, no. 1 (2021): 120. 34348759 10.1186/s13045-021-01131-0 PMC8336101

[184] M. T. Maurano , R. Humbert , E. Rynes , et al., “Systematic Localization of Common Disease‐associated Variation in Regulatory DNA,” Science (New York, NY) 337, no. 6099 (2012): 1190–1195. 10.1126/science.1222794 PMC3771521 22955828

[185] A. Mortazavi , S. Pepke , C. Jansen , et al., “Integrating and Mining the Chromatin Landscape of Cell‐type Specificity Using Self‐organizing Maps,” Genome Research 23, no. 12 (2013): 2136–2148. 24170599 10.1101/gr.158261.113 PMC3847782

[186] J. D. Buenrostro , P. G. Giresi , L. C. Zaba , H. Y. Chang , and W. J. Greenleaf , “Transposition of Native Chromatin for Fast and Sensitive Epigenomic Profiling of Open Chromatin, DNA‐binding Proteins and Nucleosome Position,” Nature Methods 10, no. 12 (2013): 1213–1238. 24097267 10.1038/nmeth.2688 PMC3959825

[187] A. J. Rubin , K. R. Parker , A. T. Satpathy , et al., “Coupled Single‐Cell CRISPR Screening and Epigenomic Profiling Reveals Causal Gene Regulatory Networks,” Cell 176, no. 1‐2 (2019): 361–376. 30580963 10.1016/j.cell.2018.11.022 PMC6329648

[188] S. Hu , L. Molina , J. Tao , et al., “NOTCH‐YAP1/TEAD‐DNMT1 Axis Drives Hepatocyte Reprogramming Into Intrahepatic Cholangiocarcinoma,” Gastroenterology 163, no. 2 (2022): 449–465. 35550144 10.1053/j.gastro.2022.05.007 PMC9329208

[189] N. Kumasaka , A. J. Knights , and D. J. Gaffney , “High‐resolution Genetic Mapping of Putative Causal Interactions Between Regions of Open Chromatin,” Nature Genetics 51, no. 1 (2019): 128–137. 30478436 10.1038/s41588-018-0278-6 PMC6330062

[190] H. Zhang , V. K. Polavarapu , P. Xing , et al., “Profiling Chromatin Accessibility in Formalin‐fixed Paraffin‐embedded Samples,” Genome Research 32, no. 1 (2022): 150–161. 34261731 10.1101/gr.275269.121 PMC8744681

[191] P. Guo , Y. Chen , L. Mao , et al., “Spatial Profiling of Chromatin Accessibility in Formalin‐fixed Paraffin‐embedded Tissues,” Nature Communications 16, no. 1 (2025): 5945. 10.1038/s41467-025-60882-3 PMC12217551 40595535

[192] J. D. Buenrostro , B. Wu , U. M. Litzenburger , et al., “Single‐cell Chromatin Accessibility Reveals Principles of Regulatory Variation,” Nature 523, no. 7561 (2015): 486–490. 26083756 10.1038/nature14590 PMC4685948

[193] K. Zhang , J. D. Hocker , M. Miller , et al., “A Single‐cell Atlas of Chromatin Accessibility in the human Genome,” Cell 184, no. 24 (2021): 5985–6001. 34774128 10.1016/j.cell.2021.10.024 PMC8664161

[194] A. T. Satpathy , J. M. Granja , K. E. Yost , et al., “Massively Parallel Single‐cell Chromatin Landscapes of human Immune Cell Development and Intratumoral T Cell Exhaustion,” Nature Biotechnology 37, no. 8 (2019): 925–936. 10.1038/s41587-019-0206-z PMC7299161 31375813

[195] G. Li , S. Fu , S. Wang , et al., “A Deep Generative Model for Multi‐view Profiling of Single‐cell RNA‐seq and ATAC‐seq Data,” Genome biology 23, no. 1 (2022): 20. 35022082 10.1186/s13059-021-02595-6 PMC8756637

[196] S. Ma , B. Zhang , L. M. Lafave , et al., “Chromatin Potential Identified by Shared Single‐Cell Profiling of RNA and Chromatin,” Cell 183, no. 4 (2020): 1103–1116. 33098772 10.1016/j.cell.2020.09.056 PMC7669735

[197] L. Tang , J. Zhang , Y. Shao , et al., “Joint Analysis of Chromatin Accessibility and Gene Expression in the Same Single Cells Reveals Cancer‐specific Regulatory Programs,” Cell Systems 16, no. 5 (2025): 101266. 40262617 10.1016/j.cels.2025.101266

[198] I. Launonen , I. Niemiec , M. Hincapié‐Otero , et al., “Chemotherapy Induces Myeloid‐driven Spatially Confined T Cell Exhaustion in Ovarian Cancer,” Cancer Cell 42, no. 12 (2024): 2045–2063. 39658541 10.1016/j.ccell.2024.11.005 PMC13359034

[199] Y. Liu , A. Sinjab , J. Min , et al., “Conserved Spatial Subtypes and Cellular Neighborhoods of Cancer‐associated Fibroblasts Revealed by Single‐cell Spatial Multi‐omics,” Cancer Cell 43, no. 5 (2025): 905–924. 40154487 10.1016/j.ccell.2025.03.004 PMC12074878

[200] Z. Du , H. Zheng , B. Huang , et al., “Allelic Reprogramming of 3D Chromatin Architecture During Early Mammalian Development,” Nature 547, no. 7662 (2017): 232–235. 28703188 10.1038/nature23263

[201] B. Ren , J. Yang , C. Wang , et al., “High‐resolution Hi‐C Maps Highlight Multiscale 3D Epigenome Reprogramming During Pancreatic Cancer Metastasis,” Journal of hematology & oncology 14, no. 1 (2021): 120. 34348759 10.1186/s13045-021-01131-0 PMC8336101

[202] P. Wu , T. Li , R. Li , et al., “3D genome of Multiple Myeloma Reveals Spatial genome Disorganization Associated With Copy Number Variations,” Nature Communications 8, no. 1 (2017): 1937. 10.1038/s41467-017-01793-w PMC5715138 29203764

[203] K. L. Mortenson , C. Dawes , E. R. Wilson , et al., “3D genomic Analysis Reveals Novel Enhancer‐hijacking Caused by Complex Structural Alterations That Drive Oncogene Overexpression,” Nature Communications 15, no. 1 (2024): 6130. 10.1038/s41467-024-50387-w PMC11271278 39033128

[204] T. S. Hsieh , G. Fudenberg , A. Goloborodko , and O. J. Rando , “Micro‐C XL: Assaying Chromosome Conformation From the Nucleosome to the Entire Genome,” Nature Methods 13, no. 12 (2016): 1009–1011. 27723753 10.1038/nmeth.4025

[205] Y. Guo , K. Krismer , M. Closser , H. Wichterle , and D. K. Gifford , “High Resolution Discovery of Chromatin Interactions,” Nucleic Acids Research 47, no. 6 (2019): e35. 30953075 10.1093/nar/gkz051 PMC6451139

[206] Q. Zhou , S. Cheng , S. Zheng , et al., “ChromLoops: A Comprehensive Database for Specific Protein‐mediated Chromatin Loops in Diverse Organisms,” Nucleic Acids Research 51, no. D1 (2023): D57–D69. 36243984 10.1093/nar/gkac893 PMC9825580

[207] J. Wang , T. Y. Huang , Y. Hou , et al., “Epigenomic Landscape and 3D Genome Structure in Pediatric High‐grade Glioma,” Science Advances 7, no. 23 (2021): g4126. 10.1126/sciadv.abg4126 PMC10166578 34078608

[208] L. Yang , F. Chen , H. Zhu , et al., “3D genome Alterations Associated With Dysregulated HOXA13 Expression in High‐risk T‐lineage Acute Lymphoblastic Leukemia,” Nature Communications 12, no. 1 (2021): 3708. 10.1038/s41467-021-24044-5 PMC8211852 34140506

[209] F. Noack , S. Vangelisti , N. Ditzer , F. Chong , M. Albert , and B. Bonev , “Joint Epigenome Profiling Reveals Cell‐type‐specific Gene Regulatory Programmes in human Cortical Organoids,” Nature Cell Biology 25, no. 12 (2023): 1873–1883. 37996647 10.1038/s41556-023-01296-5 PMC10709149

[210] R. Wang , Y. Mao , W. Wang , et al., “Systematic Evaluation of Colorectal Cancer Organoid System by Single‐cell RNA‐Seq Analysis,” Genome biology 23, no. 1 (2022): 106. 35484598 10.1186/s13059-022-02673-3 PMC9047329

[211] B. Hu , R. Wang , D. Wu , et al., “A Promising New Model: Establishment of Patient‐Derived Organoid Models Covering HPV‐Related Cervical Pre‐Cancerous Lesions and Their Cancers,” Advanced science (Weinheim, Baden‐Wurttemberg, Germany) 11, no. 12 (2024): e2302340. 38229169 10.1002/advs.202302340 PMC10966527

[212] R. A. Weinberg , “Oncogenes and Tumor Suppressor Genes,” CA: A Cancer Journal for Clinicians 44, no. 3 (1994): 160–170. 7621068 10.3322/canjclin.44.3.160

[213] P. Li and W. J. Leonard , “Chromatin Accessibility and Interactions in the Transcriptional Regulation of T Cells,” Frontiers in immunology 9, no. 2738 (2018): 2738. 30524449 10.3389/fimmu.2018.02738 PMC6262064

[214] B. Ku , D. Eisenbarth , S. Baek , et al., “PRMT1 promotes Pancreatic Cancer Development and Resistance to Chemotherapy,” Cell Rep Med 5, no. 3 (2024): 101461. 38460517 10.1016/j.xcrm.2024.101461 PMC10983040

[215] Y. Xie , M. Sahin , T. Wakamatsu , et al., “SETD2 regulates Chromatin Accessibility and Transcription to Suppress Lung Tumorigenesis,” JCI Insight 8, no. 4 (2023): e154120. 36810256 10.1172/jci.insight.154120 PMC9977508

[216] B. Wu , S. Mei , E. H. Chen , Y. Zheng , and D. Pan , “YAP Induces an Oncogenic Transcriptional Program Through TET1‐mediated Epigenetic Remodeling in Liver Growth and Tumorigenesis,” Nature Genetics 54, no. 8 (2022): 1202–1213. 35835915 10.1038/s41588-022-01119-7 PMC9357225

[217] J. Gu , J. Zhou , Q. Chen , et al., “Tumor Metabolite Lactate Promotes Tumorigenesis by Modulating MOESIN Lactylation and Enhancing TGF‐beta Signaling in Regulatory T Cells,” Cell reports 39, no. 12 (2022): 110986. 35732125 10.1016/j.celrep.2022.110986

[218] T. Wang , Z. Ye , Z. Li , et al., “Lactate‐induced Protein Lactylation: A Bridge Between Epigenetics and Metabolic Reprogramming in Cancer,” Cell Proliferation 56, no. 10 (2023): e13478. 37060186 10.1111/cpr.13478 PMC10542650

[219] T. Ding , H. Xu , X. Zhang , et al., “Prohibitin 2 Orchestrates Long Noncoding RNA and Gene Transcription to Accelerate Tumorigenesis,” Nature Communications 15, no. 1 (2024): 8385. 10.1038/s41467-024-52425-z PMC11436821 39333493

[220] P. Yu , D. Hou , B. Chang , et al., “SMARCA5 reprograms AKR1B1‐mediated Fructose Metabolism to Control Leukemogenesis,” Developmental Cell 59, no. 15 (2024): 1954–1971. 38776924 10.1016/j.devcel.2024.04.023

[221] X. Chen , M. Huang , D. Fan , et al., “CARM1 hypermethylates the NuRD Chromatin Remodeling Complex to Promote Cell Cycle Gene Expression and Breast Cancer Development,” Nucleic Acids Research 52, no. 12 (2024): 6811–6829. 38676947 10.1093/nar/gkae329 PMC11229315

[222] W. Liu , Y. Zeng , X. Hao , et al., “JARID2 coordinates With the NuRD Complex to Facilitate Breast Tumorigenesis Through Response to Adipocyte‐derived Leptin,” Cancer Commun (Lond) 43, no. 10 (2023): 1117–1142. 37658635 10.1002/cac2.12479 PMC10565380

[223] B. Wang , S. Zhang , Y. Guo , et al., “CBX2 as a Therapeutic Target in Colorectal Cancer: Insights Into the Altered Chromatin Accessibility via RUNX1‐CBX2‐MAP4K1 Axis,” Oncogene 44, no. 13 (2025): 909–926. 40082555 10.1038/s41388-025-03331-1

[224] S. Preston‐Alp , L. B. Caruso , C. Su , et al., “Decitabine Disrupts EBV Genomic Epiallele DNA Methylation Patterns Around CTCF Binding Sites to Increase Chromatin Accessibility and Lytic Transcription in Gastric Cancer,” MBio 14, no. 5 (2023): e39623. 10.1128/mbio.00396-23 PMC10653948 37606370

[225] N. Jing , X. Du , Y. Liang , et al., “PAX6 promotes Neuroendocrine Phenotypes of Prostate Cancer via Enhancing MET/STAT5A‐mediated Chromatin Accessibility,” Journal of Experimental & Clinical Cancer Research 43, no. 1 (2024): 144. 38745318 10.1186/s13046-024-03064-1 PMC11094950

[226] K. A. Orlando , A. K. Douglas , A. Abudu , et al., “Re‐expression of SMARCA4/BRG1 in Small Cell Carcinoma of Ovary, Hypercalcemic Type (SCCOHT) Promotes an Epithelial‐Like Gene Signature Through an AP‐1‐dependent Mechanism,” Elife 9 (2020): e59073. 33355532 10.7554/eLife.59073 PMC7813545

[227] L. Li , J. Kim , W. Lu , et al., “HMGA1 chromatin Regulators Induce Transcriptional Networks Involved in GATA2 and Proliferation During MPN Progression,” Blood 139, no. 18 (2022): 2797–2815. 35286385 10.1182/blood.2021013925 PMC9074401

[228] X. Sun , O. Klingbeil , B. Lu , et al., “BRD8 maintains Glioblastoma by Epigenetic Reprogramming of the p53 Network,” Nature 613, no. 7942 (2023): 195–202. 36544023 10.1038/s41586-022-05551-x PMC10189659

[229] Y. Chen , R. Zhuo , L. Sun , et al., “Super‐enhancer‐driven IRF2BP2 Enhances ALK Activity and Promotes Neuroblastoma Cell Proliferation,” Neuro‐oncol 26, no. 10 (2024): 1878–1894. 38864832 10.1093/neuonc/noae109 PMC11449008

[230] L. Cao , W. Li , J. Yang , et al., “Inflammatory Cytokine‐induced Expression of MASTL Is Involved in Hepatocarcinogenesis by Regulating Cell Cycle Progression,” Oncology letters 17, no. 3 (2019): 3163–3172. 30867746 10.3892/ol.2019.9983 PMC6396276

[231] T. Zhang , W. Xia , X. Song , et al., “Super‐enhancer Hijacking LINC01977 Promotes Malignancy of Early‐stage Lung Adenocarcinoma Addicted to the Canonical TGF‐beta/SMAD3 Pathway,” Journal of hematology & oncology 15, no. 1 (2022): 114. 35982471 10.1186/s13045-022-01331-2 PMC9389757

[232] R. Moscona , S. M. Janssen , M. Elchebly , A. I. Papadakis , E. Rubin , and A. Spatz , “BORIS/CTCFL‐mediated Chromatin Accessibility Alterations Promote a Pro‐invasive Transcriptional Signature in Melanoma Cells,” Pigment cell & melanoma research 36, no. 3‐4 (2023): 299–313. 37082838 10.1111/pcmr.13089

[233] S. Zhang , P. Li , J. Li , et al., “Chromatin Accessibility Uncovers KRAS‐driven FOSL2 Promoting Pancreatic Ductal Adenocarcinoma Progression Through Up‐regulation of CCL28,” British Journal of Cancer 129, no. 3 (2023): 426–443. 37380804 10.1038/s41416-023-02313-y PMC10403592

[234] C. Zheng , M. Liu , Y. Ge , Y. Qian , and H. Fan , “HBx Increases Chromatin Accessibility and ETV4 Expression to Regulate Dishevelled‐2 and Promote HCC Progression,” Cell death & disease 13, no. 2 (2022): 116. 35121725 10.1038/s41419-022-04563-9 PMC8816937

[235] M. F. Emmons , R. L. Bennett , A. Riva , et al., “HDAC8‐mediated Inhibition of EP300 Drives a Transcriptional state That Increases Melanoma Brain Metastasis,” Nature Communications 14, no. 1 (2023): 7759. 10.1038/s41467-023-43519-1 PMC10686983 38030596

[236] S. E. Pierce , J. M. Granja , M. R. Corces , et al., “LKB1 inactivation Modulates Chromatin Accessibility to Drive Metastatic Progression,” Nature Cell Biology 23, no. 8 (2021): 915–924. 34341533 10.1038/s41556-021-00728-4 PMC8355205

[237] Y. Wu , Y. Zhao , L. Huan , et al., “An LTR Retrotransposon‐Derived Long Noncoding RNA lncMER52A Promotes Hepatocellular Carcinoma Progression by Binding p120‐Catenin,” Cancer Research 80, no. 5 (2020): 976–987. 31874857 10.1158/0008-5472.CAN-19-2115

[238] M. Zhang , Z. Z. Liu , K. Aoshima , et al., “CECR2 drives Breast Cancer Metastasis by Promoting NF‐kappaB Signaling and Macrophage‐mediated Immune Suppression,” Science Translational Medicine 14, no. 630 (2022): f5473. 10.1126/scitranslmed.abf5473 PMC9003667 35108062

[239] Y. Xie , M. Sahin , S. Sinha , et al., “SETD2 loss Perturbs the Kidney Cancer Epigenetic Landscape to Promote Metastasis and Engenders Actionable Dependencies on Histone Chaperone Complexes,” Nat Cancer 3, no. 2 (2022): 188–202. 35115713 10.1038/s43018-021-00316-3 PMC8885846

[240] S. Li , M. Yang , S. Teng , et al., “Chromatin Accessibility Dynamics in Colorectal Cancer Liver Metastasis: Uncovering the Liver Tropism at Single Cell Resolution,” Pharmacological Research 195, no. 106896 (2023): 106896. 37633511 10.1016/j.phrs.2023.106896

[241] W. D. Pontius , E. S. Hong , Z. J. Faber , et al., “Temporal Chromatin Accessibility Changes Define Transcriptional States Essential for Osteosarcoma Metastasis,” Nature Communications 14, no. 1 (2023): 7209. 10.1038/s41467-023-42656-x PMC10632377 37938582

[242] Y. Liu , X. Wang , Y. Zhu , et al., “The CTCF/LncRNA‐PACERR Complex Recruits E1A Binding Protein p300 to Induce Pro‐tumour Macrophages in Pancreatic Ductal Adenocarcinoma via Directly Regulating PTGS2 Expression,” Clinical and translational medicine 12, no. 2 (2022): e654. 35184402 10.1002/ctm2.654 PMC8858628

[243] C. S. Amara , K. R. Kami Reddy , and Y. Yuntao , “The IL6/JAK/STAT3 Signaling Axis Is a Therapeutic Vulnerability in SMARCB1‐deficient Bladder Cancer,” Nature Communications 15, no. 1 (2024): 1373. 10.1038/s41467-024-45132-2 PMC10867091 38355560

[244] D. Han , M. Labaf , Y. Zhao , et al., “Androgen Receptor Splice Variants Drive Castration‐resistant Prostate Cancer Metastasis by Activating Distinct Transcriptional Programs,” Journal of Clinical Investigation 134, no. 11 (2024): e168649. 38687617 10.1172/JCI168649 PMC11142739

[245] P. S. Ward and C. B. Thompson , “Metabolic Reprogramming: A Cancer Hallmark Even warburg Did Not Anticipate,” Cancer Cell 21, no. 3 (2012): 297–308. 22439925 10.1016/j.ccr.2012.02.014 PMC3311998

[246] N. N. Pavlova and C. B. Thompson , “The Emerging Hallmarks of Cancer Metabolism,” Cell metabolism 23, no. 1 (2016): 27–47. 26771115 10.1016/j.cmet.2015.12.006 PMC4715268

[247] Z. Zhang , Y. Gao , Y. Qian , et al., “The Lyn/RUVBL1 Complex Promotes Colorectal Cancer Liver Metastasis by Regulating Arachidonic Acid Metabolism Through Chromatin Remodeling,” Adv Sci (Weinh) 12, no. 5 (2025): e2406562. 39665272 10.1002/advs.202406562 PMC11792055

[248] C. Zhang , L. Chen , Y. Liu , et al., “Downregulated METTL14 Accumulates BPTF That Reinforces Super‐enhancers and Distal Lung Metastasis via Glycolytic Reprogramming in Renal Cell Carcinoma,” Theranostics 11, no. 8 (2021): 3676–3693. 33664855 10.7150/thno.55424 PMC7914369

[249] X. Liu , Z. Li , Z. Wang , et al., “Chromatin Remodeling Induced by ARID1A Loss in Lung Cancer Promotes Glycolysis and Confers JQ1 Vulnerability,” Cancer Research 82, no. 5 (2022): 791–804. 34987057 10.1158/0008-5472.CAN-21-0763

[250] Z. Liu , H. Chen , L. Zheng , L. Sun , and L. Shi , “Angiogenic Signaling Pathways and Anti‐angiogenic Therapy for Cancer,” Signal Transduct Target Ther 8, no. 1 (2023): 198. 37169756 10.1038/s41392-023-01460-1 PMC10175505

[251] L. Bao , A. Kumar , M. Zhu , et al., “SAP30 promotes Breast Tumor Progression by Bridging the Transcriptional Corepressor SIN3 Complex and MLL1,” Journal of Clinical Investigation 133, no. 17 (2023): e168362. 37655663 10.1172/JCI168362 PMC10471174

[252] Q. Huang , D. Wu , J. Zhao , et al., “TFAM Loss Induces Nuclear Actin Assembly Upon mDia2 Malonylation to Promote Liver Cancer Metastasis,” Embo Journal 41, no. 11 (2022): e110324. 35451091 10.15252/embj.2021110324 PMC9156967

[253] L. Zhang , J. Lu , R. Liu , et al., “Chromatin Accessibility Analysis Reveals That TFAP2A Promotes Angiogenesis in Acquired Resistance to Anlotinib in Lung Cancer Cells,” Acta Pharmacologica Sinica 41, no. 10 (2020): 1357–1365. 32415222 10.1038/s41401-020-0421-7 PMC7608858

[254] S. Bastola , M. S. Pavlyukov , N. Sharma , et al., “Endothelial‐secreted Endocan Activates PDGFRA and Regulates Vascularity and Spatial Phenotype in Glioblastoma,” Nature Communications 16, no. 1 (2025): 471. 10.1038/s41467-024-55487-1 PMC11707362 39773984

[255] J. Lee , C. You , G. Kwon , et al., “Integration of Epigenomic and Transcriptomic Profiling Uncovers EZH2 Target Genes Linked to Cysteine Metabolism in Hepatocellular Carcinoma,” Cell death & disease 15, no. 11 (2024): 801. 39516467 10.1038/s41419-024-07198-0 PMC11549485

[256] R. Fontana , A. Mestre‐Farrera , and J. Yang , “Update on Epithelial‐Mesenchymal Plasticity in Cancer Progression,” Annu Rev Pathol 19 (2024): 133–156. 37758242 10.1146/annurev-pathmechdis-051222-122423 PMC10872224

[257] G. E. Thomas , G. Egan , L. Garcia‐Prat , et al., “The Metabolic Enzyme Hexokinase 2 Localizes to the Nucleus in AML and Normal Haematopoietic Stem and Progenitor Cells to Maintain Stemness,” Nature Cell Biology 24, no. 6 (2022): 872–884. 35668135 10.1038/s41556-022-00925-9 PMC9203277

[258] S. Munoz‐Galvan , E. M. Verdugo‐Sivianes , J. M. Santos‐Pereira , P. Estevez‐Garcia , and A. Carnero , “Essential Role of PLD2 in Hypoxia‐induced Stemness and Therapy Resistance in Ovarian Tumors,” Journal of Experimental & Clinical Cancer Research 43, no. 1 (2024): 57. 38403587 10.1186/s13046-024-02988-y PMC10895852

[259] L. Wang , C. Deng , Q. Luo , et al., “Inhibition of Arid1a Increases Stem/Progenitor Cell‐Like Properties of Liver Cancer,” Cancer Letters 546 (2022): 215869. 35964817 10.1016/j.canlet.2022.215869

[260] M. Hagiwara , A. Fushimi , N. Yamashita , et al., “MUC1‐C Activates the PBAF Chromatin Remodeling Complex in Integrating Redox Balance With Progression of human Prostate Cancer Stem Cells,” Oncogene 40, no. 30 (2021): 4930–4940. 34163028 10.1038/s41388-021-01899-y PMC8321896

[261] D. Kufe , “Dependence on MUC1‐C in Progression of Neuroendocrine Prostate Cancer,” International Journal of Molecular Sciences 24, no. 4 (2023): 3719. 36835130 10.3390/ijms24043719 PMC9967814

[262] S. Song , L. Wang , X. Jiang , et al., “CircHULC Accelerates the Growth of human Liver Cancer Stem Cells by Enhancing Chromatin Reprogramming and Chromosomal Instability via Autophagy,” Cell Signalling 109 (2023): 110772. 37321526 10.1016/j.cellsig.2023.110772

[263] A. Nikolic , F. Maule , A. Bobyn , et al., “MacroH2A2 antagonizes Epigenetic Programs of Stemness in Glioblastoma,” Nature Communications 14, no. 1 (2023): 3062. 10.1038/s41467-023-38919-2 PMC10224928 37244935

[264] D. S. Vinay , E. P. Ryan , G. Pawelec , et al., “Immune Evasion in Cancer: Mechanistic Basis and Therapeutic Strategies,” Seminars in Cancer Biology 35 (2015): S185–S198. Suppl. 25818339 10.1016/j.semcancer.2015.03.004

[265] K. Kumar , A. Pareek , and R. Kaur , “SWI/SNF Complex‐mediated Chromatin Remodeling in Candida glabrata Promotes Immune Evasion,” Iscience 27, no. 4 (2024): 109607. 38632999 10.1016/j.isci.2024.109607 PMC11022050

[266] G. Leuzzi , A. Vasciaveo , A. Taglialatela , et al., “SMARCAL1 is a Dual Regulator of Innate Immune Signaling and PD‐L1 Expression That Promotes Tumor Immune Evasion,” Cell 187, no. 4 (2024): 861–881. 38301646 10.1016/j.cell.2024.01.008 PMC10980358

[267] E. Chen , J. Wu , J. Huang , et al., “FLI1 promotes IFN‐gamma‐induced Kynurenine Production to Impair Anti‐tumor Immunity,” Nature Communications 15, no. 1 (2024): 4590. 10.1038/s41467-024-48397-9 PMC11139667 38816360

[268] B. Zhuo , Q. Zhang , T. Xie , et al., “Integrative Epigenetic Analysis Reveals AP‐1 Promotes Activation of Tumor‐infiltrating Regulatory T Cells in HCC,” Cellular and Molecular Life Sciences 80, no. 4 (2023): 103. 36941472 10.1007/s00018-023-04746-3 PMC11071886

[269] L. Zhang , H. Li , and R. Shereda , “DNMT and EZH2 Inhibitors Synergize to Activate Therapeutic Targets in Hepatocellular Carcinoma,” Cancer Letters 548 (2022): 215899. 36087682 10.1016/j.canlet.2022.215899 PMC9563073

[270] K. Ranjan , B. K. Rajendran , I. U. Deen , et al., “IL‐4 Mediated TAP2 Downregulation Is a Dominant and Reversible Mechanism of Immune Evasion and Immunotherapy Resistance in Non‐small Cell Lung Cancer,” Molecular cancer 24, no. 1 (2025): 80. 40091029 10.1186/s12943-025-02276-z PMC11912681

[271] X. Cui , D. Huo , Q. Wang , et al., “RUNX1/NPM1/H3K4me3 complex Contributes to Extracellular Matrix Remodeling via Enhancing FOSL2 Transcriptional Activation in Glioblastoma,” Cell death & disease 15, no. 1 (2024): 98. 38286983 10.1038/s41419-024-06481-4 PMC10825180

[272] H. Zhang , G. Chen , X. Feng , et al., “Targeting WDxR Motif Reprograms Immune Microenvironment and Inhibits Hepatocellular Carcinoma Progression,” EMBO Molecular Medicine 15, no. 5 (2023): e15924. 36947051 10.15252/emmm.202215924 PMC10165360

[273] S. Zahraeifard , Z. Xiao , J. Y. So , et al., “Loss of Tumor Suppressors Promotes Inflammatory Tumor Microenvironment and Enhances LAG3+T Cell Mediated Immune Suppression,” Nature Communications 15, no. 1 (2024): 5873. 10.1038/s41467-024-50262-8 PMC11245525 38997291

[274] L. Ka‐Yue Chow , D. Lai‐Shun Chung , L. Tao , et al., “Epigenomic Landscape Study Reveals Molecular Subtypes and EBV‐associated Regulatory Epigenome Reprogramming in Nasopharyngeal Carcinoma,” EBioMedicine 86 (2022): 104357. 36371985 10.1016/j.ebiom.2022.104357 PMC9663866

[275] X. Zheng , Y. Luo , Y. Xiong , et al., “Tumor Cell‐intrinsic SETD2 Inactivation Sensitizes Cancer Cells to Immune Checkpoint Blockade Through the NR2F1‐STAT1 Pathway,” Journal for ImmunoTherapy of Cancer 11, no. 12 (2023): e007678. 38056895 10.1136/jitc-2023-007678 PMC10711831

[276] Y. Wu , J. Zhao , H. Zhu , et al., “SPACE: A Web Server for Linking Chromatin Accessibility With Clinical Phenotypes and the Immune Microenvironment in Pan‐cancer Analysis,” Cell Mol Immunol 17, no. 12 (2020): 1294–1296. 32238917 10.1038/s41423-020-0416-9 PMC7784913

[277] H. Meng , H. Miao , Y. Zhang , et al., “YBX1 promotes Homologous Recombination and Resistance to Platinum‐induced Stress in Ovarian Cancer by Recognizing m5C Modification,” Cancer Letters 597 (2024): 217064. 38880223 10.1016/j.canlet.2024.217064

[278] B. Zhang , Q. Long , S. Wu , et al., “KDM4 orchestrates Epigenomic Remodeling of Senescent Cells and Potentiates the Senescence‐Associated Secretory Phenotype,” Aging Cell 24, no. 10 (2025): e70194. 40849896 10.1111/acel.70194 PMC12507408

[279] Y. Nan , Q. Luo , X. Wu , et al., “DLGAP1‐AS2‐Mediated Phosphatidic Acid Synthesis Activates YAP Signaling and Confers Chemoresistance in Squamous Cell Carcinoma,” Cancer Research 82, no. 16 (2022): 2887–2903. 35731019 10.1158/0008-5472.CAN-22-0717

[280] F. J. de Miguel , C. Gentile , W. W. Feng , et al., “Mammalian SWI/SNF Chromatin Remodeling Complexes Promote Tyrosine Kinase Inhibitor Resistance in EGFR‐mutant Lung Cancer,” Cancer Cell 41, no. 8 (2023): 1516–1534. 37541244 10.1016/j.ccell.2023.07.005 PMC10957226

[281] X. Li , S. Wang , Y. Xie , et al., “Deacetylation Induced Nuclear Condensation of HP1gamma Promotes Multiple Myeloma Drug Resistance,” Nature Communications 14, no. 1 (2023): 1290. 10.1038/s41467-023-37013-x PMC9998874 36894562

[282] Y. Xue , T. Yin , S. Yuan , et al., “CYP1B1 promotes PARPi‐resistance via Histone H1.4 Interaction and Increased Chromatin Accessibility in Ovarian Cancer,” Drug Resistance Updates 77 (2024): 101151. 39395328 10.1016/j.drup.2024.101151

[283] S. Ma , T. Tang , G. Probst , et al., “Transcriptional Repression of Estrogen Receptor Alpha by YAP Reveals the Hippo Pathway as Therapeutic Target for ER(+) Breast Cancer,” Nature Communications 13, no. 1 (2022): 1061. 10.1038/s41467-022-28691-0 PMC8881512 35217640

[284] R. Blawski and E. Toska , “A Unique FOXA1‐Associated Chromatin state Dictates Therapeutic Resistance in Lobular Breast Cancer,” Cancer Research 82, no. 20 (2022): 3668–3670. 36245246 10.1158/0008-5472.CAN-22-2594 PMC10084780

[285] C. Qian , Q. Yang , M. Rotinen , et al., “ONECUT2 acts as a Lineage Plasticity Driver in Adenocarcinoma as Well as Neuroendocrine Variants of Prostate Cancer,” Nucleic Acids Research 52, no. 13 (2024): 7740–7760. 38932701 10.1093/nar/gkae547 PMC11260453

[286] N. Leppanen , H. Kaljunen , E. Takala , et al., “SIX2 promotes Cell Plasticity via Wnt/Beta‐catenin Signalling in Androgen Receptor Independent Prostate Cancer,” Nucleic Acids Research 52, no. 10 (2024): 5610–5623. 38554106 10.1093/nar/gkae206 PMC11162805

[287] D. G. Coffey , P. Ataca Atilla , E. Atilla , et al., “Single‐cell Analysis of the Multiple Myeloma Microenvironment After Gamma‐secretase Inhibition and CAR T‐cell Therapy,” Blood 145, no. 2 (2025): 220–233. 39374522 10.1182/blood.2024025231 PMC11738034

[288] S. Aminov , O. Giricz , D. T. Melnekoff , et al., “Immunotherapy‐resistant Acute Lymphoblastic Leukemia Cells Exhibit Reduced CD19 and CD22 Expression and BTK Pathway Dependency,” Journal of Clinical Investigation 134, no. 8 (2024): e175199. 38376944 10.1172/JCI175199 PMC11014656

[289] Y. Tu , H. Wu , C. Zhong , et al., “Pharmacological Activation of STAT1‐GSDME Pyroptotic Circuitry Reinforces Epigenetic Immunotherapy for Hepatocellular Carcinoma,” Gut 74, no. 4 (2025): 613–627. 39486886 10.1136/gutjnl-2024-332281 PMC12013592

[290] Y. Yang , X. Jia , Q. Lu , et al., “Exosomal DEK Removes Chemoradiotherapy Resistance by Triggering Quiescence Exit of Breast Cancer Stem Cells,” Oncogene 41, no. 18 (2022): 2624–2637. 35351996 10.1038/s41388-022-02278-x

[291] D. S. Nin , C. Wujanto , T. Z. Tan , et al., “GAGE Mediates Radio Resistance in Cervical Cancers via the Regulation of Chromatin Accessibility,” Cell reports 36, no. 9 (2021): 109621. 34469741 10.1016/j.celrep.2021.109621

[292] M. Svetlicic , A. Bomhard , C. Sterr , et al., “Alpha Radiation as a Way to Target Heterochromatic and Gamma Radiation‐Exposed Breast Cancer Cells,” Cells 9, no. 5 (2020): 1165. 32397212 10.3390/cells9051165 PMC7291130

[293] P. Ulz , G. G. Thallinger , M. Auer , et al., “Inferring Expressed Genes by Whole‐genome Sequencing of Plasma DNA,” Nature Genetics 48, no. 10 (2016): 1273–1278. 27571261 10.1038/ng.3648

[294] J. Zhang , J. Li , J. B. Saucier , et al., “Non‐invasive Prenatal Sequencing for Multiple Mendelian Monogenic Disorders Using Circulating Cell‐free Fetal DNA,” Nature Medicine 25, no. 3 (2019): 439–447. 10.1038/s41591-018-0334-x 30692697

[295] J. Li , J. Chen , X. Sun , et al., “Uncovering Chromatin Accessibility and Cancer Diagnostic Potential via Cell‐free DNA Utilization,” Sci Bull (Beijing) 69, no. 19 (2024): 2987–2992. 38664094 10.1016/j.scib.2024.04.013

[296] Y. Bian , Y. Gao , and H. Lin , “Non‐invasive Diagnosis of Esophageal Cancer by a Simplified Circulating Cell‐free DNA Methylation Assay Targeting OTOP2 and KCNA3: A Double‐blinded, Multicenter, Prospective Study,” Journal of hematology & oncology 17, no. 1 (2024): 47. 38890756 10.1186/s13045-024-01565-2 PMC11186155

[297] P. Taklifi , F. Palizban , and M. Mehrmohamadi , “Integrating Chromatin Accessibility States in the Design of Targeted Sequencing Panels for Liquid Biopsy,” Scientific Reports 12, no. 1 (2022): 10447. 35729208 10.1038/s41598-022-14675-z PMC9213477

[298] X. Liu , Y. Peng , and J. Wang , “Integrative Analysis of DNA Methylation and Gene Expression Profiles Identified Potential Breast Cancer‐specific Diagnostic Markers,” Bioscience Reports 40, no. 5 (2020): BSR20201053. 32412047 10.1042/BSR20201053 PMC7263199

[299] P. Ulz , S. Perakis , Q. Zhou , et al., “Inference of Transcription Factor Binding From Cell‐free DNA Enables Tumor Subtype Prediction and Early Detection,” Nature Communications 10, no. 1 (2019): 4666. 10.1038/s41467-019-12714-4 PMC6789008 31604930

[300] A. Doebley , M. Ko , H. Liao , et al., “A Framework for Clinical Cancer Subtyping From Nucleosome Profiling of Cell‐free DNA,” Nature Communications 13, no. 1 (2022): 7475. 10.1038/s41467-022-35076-w PMC9719521 36463275

[301] Y. Wu , N. V. Terekhanova , W. Caravan , et al., “Epigenetic and Transcriptomic Characterization Reveals Progression Markers and Essential Pathways in Clear Cell Renal Cell Carcinoma,” Nature Communications 14, no. 1 (2023): 1681. 10.1038/s41467-023-37211-7 PMC10042888 36973268

[302] M. Yuan , M. E. Barefoot , K. Peterson , et al., “Loss of ANCO1 Expression Regulates Chromatin Accessibility and Drives Progression of Early‐Stage Triple‐Negative Breast Cancer,” International Journal of Molecular Sciences 24, no. 14 (2023): 11505. 37511268 10.3390/ijms241411505 PMC10380654

[303] K. Xu , W. Zhang , C. Wang , et al., “Integrative Analyses of scRNA‐seq and scATAC‐seq Reveal CXCL14 as a Key Regulator of Lymph Node Metastasis in Breast Cancer,” Human Molecular Genetics 30, no. 5 (2021): 370–380. 33564857 10.1093/hmg/ddab042

[304] L. M. Lafave , V. K. Kartha , S. Ma , et al., “Epigenomic state Transitions Characterize Tumor Progression in Mouse Lung Adenocarcinoma,” Cancer Cell 38, no. 2 (2020): 212–228. 32707078 10.1016/j.ccell.2020.06.006 PMC7641015

[305] S. Dhara , S. Chhangawala , H. Chintalapudi , et al., “Pancreatic Cancer Prognosis Is Predicted by an ATAC‐array Technology for Assessing Chromatin Accessibility,” Nature Communications 12, no. 1 (2021): 3044. 10.1038/s41467-021-23237-2 PMC8144607 34031415

[306] G. Zhu , J. Liu , Y. Li , et al., “ARID1B deficiency Leads to Impaired DNA Damage Response and Activated cGAS‐STING Pathway in Non‐Small Cell Lung Cancer,” Journal of Cancer 15, no. 9 (2024): 2601–2612. 38577613 10.7150/jca.91955 PMC10988295

[307] Y. Huang , J. C. Lai , P. Peng , K. Wei , and K. Wu , “Chromatin Accessibility Analysis Identifies GSTM1 as a Prognostic Marker in human Glioblastoma Patients,” Clin Epigenetics 13, no. 1 (2021): 201. 34732244 10.1186/s13148-021-01181-8 PMC8565064

[308] C. M. Niemeyer , C. Flotho , D. B. Lipka , et al., “Response to Upfront Azacitidine in Juvenile Myelomonocytic Leukemia in the AZA‐JMML‐001 Trial,” Blood Adv 5, no. 14 (2021): 2901–2908. 34297046 10.1182/bloodadvances.2020004144 PMC8341358

[309] D. S. Aschenbrenner , “Drug Receives New Indication for Juvenile Myelomonocytic Leukemia,” American Journal of Nursing 122, no. 9 (2022): 25. 10.1097/01.NAJ.0000874108.90975.bc 36005792

[310] L. A. Cowan , S. Talwar , and A. S. Yang , “Will DNA Methylation Inhibitors Work in Solid Tumors? A Review of the Clinical Experience With Azacitidine and Decitabine in Solid Tumors,” Epigenomics 2, no. 1 (2010): 71–86. 22122748 10.2217/epi.09.44

[311] Y. Yamada , V. B. Venkadakrishnan , K. Mizuno , et al., “Targeting DNA Methylation and B7‐H3 in RB1‐deficient and Neuroendocrine Prostate Cancer,” Science Translational Medicine 15, no. 722 (2023): f6732. 10.1126/scitranslmed.adf6732 PMC10954288 37967200

[312] S. Gomez , O. L. Cox , R. R. R. Walker , et al., “Inhibiting DNA Methylation and RNA Editing Upregulates Immunogenic RNA to Transform the Tumor Microenvironment and Prolong Survival in Ovarian Cancer,” Journal for ImmunoTherapy of Cancer 10, no. 11 (2022): e004974. 36343976 10.1136/jitc-2022-004974 PMC9644370

[313] J. Lai , Y. Fu , S. Tian , et al., “Zebularine Elevates STING Expression and Enhances cGAMP Cancer Immunotherapy in Mice,” Molecular Therapy 29, no. 5 (2021): 1758–1771. 33571681 10.1016/j.ymthe.2021.02.005 PMC8116609

[314] T. S. Rodems , E. Heninger , C. N. Stahlfeld , et al., “Reversible Epigenetic Alterations Regulate Class I HLA Loss in Prostate Cancer,” Communications Biology 5, no. 1 (2022): 897. 36050516 10.1038/s42003-022-03843-6 PMC9437063

[315] R. Chen , M. Zhang , Y. Zhou , et al., “The Application of Histone Deacetylases Inhibitors in Glioblastoma,” Journal of Experimental & Clinical Cancer Research 39, no. 1 (2020): 138. 32682428 10.1186/s13046-020-01643-6 PMC7368699

[316] C. Yu and S. Zhuang , “Histone Methyltransferases as Therapeutic Targets for Kidney Diseases,” Frontiers in pharmacology 10 (2019): 1393. 31866860 10.3389/fphar.2019.01393 PMC6908484

[317] J. Qiu , S. Sharma , R. A. Rollins , and T. A. Paul , “The Complex Role of EZH2 in the Tumor Microenvironment: Opportunities and Challenges for Immunotherapy Combinations,” Future Med Chem 12, no. 15 (2020): 1415–1430. 32723083 10.4155/fmc-2020-0072

[318] R. Straining and W. Eighmy , “Tazemetostat: EZH2 Inhibitor,” J Adv Pract Oncol 13, no. 2 (2022): 158–163. 35369397 10.6004/jadpro.2022.13.2.7 PMC8955562

[319] A. Suraweera , K. J. O'Byrne , and D. J. Richard , “Epigenetic Drugs in Cancer Therapy,” Cancer and Metastasis Reviews 44, no. 1 (2025): 37. 40011240 10.1007/s10555-025-10253-7 PMC11865116

[320] L. Zhang , Y. Chen , Z. Li , C. Lin , T. Zhang , and G. Wang , “Development of JmjC‐domain‐containing Histone Demethylase (KDM2‐7) Inhibitors for Cancer Therapy,” Drug Discov Today 28, no. 5 (2023): 103519. 36754142 10.1016/j.drudis.2023.103519

[321] Y. Li and E. Seto , “HDACs and HDAC Inhibitors in Cancer Development and Therapy,” Cold Spring Harbor perspectives in medicine 6, no. 10 (2016): a026831. 27599530 10.1101/cshperspect.a026831 PMC5046688

[322] M. J. Ramaiah , A. D. Tangutur , and R. R. Manyam , “Epigenetic Modulation and Understanding of HDAC Inhibitors in Cancer Therapy,” Life Sciences 277 (2021): 119504. 33872660 10.1016/j.lfs.2021.119504

[323] T. Eckschlager , J. Plch , M. Stiborova , and J. Hrabeta , “Histone Deacetylase Inhibitors as Anticancer Drugs,” International Journal of Molecular Sciences 18, no. 7 (2017): 1414. 28671573 10.3390/ijms18071414 PMC5535906

[324] C. Wang , M. Huang , Y. Lin , et al., “ENO2‐derived Phosphoenolpyruvate Functions as an Endogenous Inhibitor of HDAC1 and Confers Resistance to Antiangiogenic Therapy,” Nat Metab 5, no. 10 (2023): 1765–1786. 37667133 10.1038/s42255-023-00883-y

[325] W. Yang , Y. Feng , J. Zhou , et al., “A Selective HDAC8 Inhibitor Potentiates Antitumor Immunity and Efficacy of Immune Checkpoint Blockade in Hepatocellular Carcinoma,” Science Translational Medicine 13, no. 588 (2021): eaaz6804. 33827976 10.1126/scitranslmed.aaz6804

[326] A. Mormino , G. Cocozza , G. Fontemaggi , et al., “Histone‐deacetylase 8 Drives the Immune Response and the Growth of Glioma,” Glia 69, no. 11 (2021): 2682–2698. 34310727 10.1002/glia.24065 PMC8457175

[327] I. Psilopatis , A. Pergaris , C. Giaginis , and S. Theocharis , “Histone Deacetylase Inhibitors: A Promising Therapeutic Alternative for Endometrial Carcinoma,” Disease Markers 2021 (2021): 7850688. 34804263 10.1155/2021/7850688 PMC8604582

[328] Y. Jia , J. Li , W. Mei , et al., “Pan‐HDAC Inhibitor LAQ824 Inhibits the Progression of Pancreatic Ductal Adenocarcinoma and Suppresses Immune Escape by Promoting Antigen Presentation,” International Immunopharmacology 154 (2025): 114528. 40158429 10.1016/j.intimp.2025.114528

[329] D. T. Nguyen , W. Yang , A. Renganathan , et al., “Acetylated HOXB13 Regulated Super Enhancer Genes Define Therapeutic Vulnerabilities of Castration‐Resistant Prostate Cancer,” Clinical Cancer Research 28, no. 18 (2022): 4131–4145. 35849143 10.1158/1078-0432.CCR-21-3603 PMC9481728

[330] S. J. Welsh , B. G. Barwick , E. W. Meermeier , et al., “Transcriptional Heterogeneity Overcomes Super‐Enhancer Disrupting Drug Combinations in Multiple Myeloma,” Blood Cancer Discov 5, no. 1 (2024): 34–55. 37767768 10.1158/2643-3230.BCD-23-0062 PMC10772542

[331] H. A. Malone and C. W. M. Roberts , “Chromatin Remodellers as Therapeutic Targets,” Nat Rev Drug Discovery 23, no. 9 (2024): 661–681. 39014081 10.1038/s41573-024-00978-5 PMC11534152

[332] L. Cui , R. Liu , S. Han , et al., “Targeting Arachidonic Acid Metabolism Enhances Immunotherapy Efficacy in ARID1A‐Deficient Colorectal Cancer,” Cancer Research 85, no. 5 (2025): 925–941. 39652583 10.1158/0008-5472.CAN-24-1611 PMC11873721

[333] K. C. Helming , X. Wang , B. G. Wilson , et al., “ARID1B is a Specific Vulnerability in ARID1A‐mutant Cancers,” Nature Medicine 20, no. 3 (2014): 251–254. 10.1038/nm.3480 PMC3954704 24562383

[334] P. Mittal and C. W. M. Roberts , “The SWI/SNF Complex in Cancer—biology, Biomarkers and Therapy,” Nature reviews Clinical oncology 17, no. 7 (2020): 435–448. 10.1038/s41571-020-0357-3 PMC8723792 32303701

[335] E. Redin , H. Sridhar , Y. A. Zhan , et al., “SMARCA4 controls state Plasticity in Small Cell Lung Cancer Through Regulation of Neuroendocrine Transcription Factors and REST Splicing,” Journal of hematology & oncology 17, no. 1 (2024): 58. 39080761 10.1186/s13045-024-01572-3 PMC11290012

[336] W. Fiskus , J. Piel , M. Collins , et al., “BRG1/BRM Inhibitor Targets AML Stem Cells and Exerts Superior Preclinical Efficacy Combined With BET or Menin Inhibitor,” Blood 143, no. 20 (2024): 2059–2072. 38437498 10.1182/blood.2023022832 PMC11830987

[337] D. Lee , D. Lee , Y. Hwang , H. Seo , S. Lee , and J. Kwon , “The Bromodomain Inhibitor PFI‐3 Sensitizes Cancer Cells to DNA Damage by Targeting SWI/SNF,” Molecular Cancer Research 19, no. 5 (2021): 900–912. 33208498 10.1158/1541-7786.MCR-20-0289

[338] E. Panditharatna , J. G. Marques , T. Wang , et al., “BAF Complex Maintains Glioma Stem Cells in Pediatric H3K27M Glioma,” Cancer discovery 12, no. 12 (2022): 2880–2905. 36305736 10.1158/2159-8290.CD-21-1491 PMC9716260

[339] Y. Oyama , S. Shigeta , H. Tokunaga , et al., “CHD4 regulates Platinum Sensitivity Through MDR1 Expression in Ovarian Cancer: A Potential Role of CHD4 Inhibition as a Combination Therapy With Platinum Agents,” PLoS ONE 16, no. 6 (2021): e251079. 10.1371/journal.pone.0251079 PMC8221472 34161330

[340] J. Graca Marques , B. Pavlovic , Q. A. Ngo , et al., “The Chromatin Remodeler CHD4 Sustains Ewing Sarcoma Cell Survival by Controlling Global Chromatin Architecture,” Cancer Research 84, no. 2 (2024): 241–257. 37963210 10.1158/0008-5472.CAN-22-3950

[341] J. Xu , Q. Wang , E. L. H. Leung , et al., “Compound C620‐0696, a New Potent Inhibitor Targeting BPTF, the Chromatin‐remodeling Factor in Non‐small‐cell Lung Cancer,” Front Med 14, no. 1 (2020): 60–67. 31104301 10.1007/s11684-019-0694-8

[342] N. Nano , F. Ugwu , T. V. Seraphim , et al., “Sorafenib as an Inhibitor of RUVBL2,” Biomolecules 10, no. 4 (2020): 605. 32295120 10.3390/biom10040605 PMC7226205

[343] S. A. Lambert , A. Jolma , L. F. Campitelli , et al., “The human Transcription Factors,” Cell 172, no. 4 (2018): 650–665. 29425488 10.1016/j.cell.2018.01.029 PMC12908702

[344] R. Wang , A. B. Bhatt , B. A. Minden‐Birkenmaier , et al., “ZBTB18 restricts Chromatin Accessibility and Prevents Transcriptional Adaptations That Drive Metastasis,” Science Advances 9, no. 1 (2023): q3951. 10.1126/sciadv.abq3951 PMC9821869 36608120

[345] A. G. Holmes , J. B. Parker , V. Sagar , et al., “A MYC Inhibitor Selectively Alters the MYC and MAX Cistromes and Modulates the Epigenomic Landscape to Regulate Target Gene Expression,” Science Advances 8, no. 17 (2022): h3635. 10.1126/sciadv.abh3635 PMC9045724 35476451

[346] G. Tang , L. Luo , J. Zhang , et al., “LncRNA LINC01057 Promotes Mesenchymal Differentiation by Activating NF‐kappaB Signaling in Glioblastoma,” Cancer Letters 498 (2021): 152–164. 33130316 10.1016/j.canlet.2020.10.047

[347] G. Arun , S. Diermeier , M. Akerman , et al., “Differentiation of Mammary Tumors and Reduction in Metastasis Upon Malat1 lncRNA Loss,” Genes & development 30, no. 1 (2016): 34–51. 26701265 10.1101/gad.270959.115 PMC4701977

[348] H. Ye , X. Chu , Z. Cao , et al., “A Novel Targeted Therapy System for Cervical Cancer: Co‐Delivery System of Antisense LncRNA of MDC1 and Oxaliplatin Magnetic Thermosensitive Cationic Liposome Drug Carrier,” Int J Nanomedicine 16 (2021): 1051–1066. 33603368 10.2147/IJN.S258316 PMC7886386

[349] H. Li , K. Peng , K. Yang , et al., “Circular RNA Cancer Vaccines Drive Immunity in Hard‐to‐treat Malignancies,” Theranostics 12, no. 14 (2022): 6422–6436. 36168634 10.7150/thno.77350 PMC9475446

[350] J. Yang , J. Zhu , J. Sun , et al., “Intratumoral Delivered Novel Circular mRNA Encoding Cytokines for Immune Modulation and Cancer Therapy,” Mol Ther Nucleic Acids 30 (2022): 184–197. 36156907 10.1016/j.omtn.2022.09.010 PMC9482165

[351] D. Niu , Y. Wu , and J. Lian , “Circular RNA Vaccine in Disease Prevention and Treatment,” Signal Transduct Target Ther 8, no. 1 (2023): 341. 37691066 10.1038/s41392-023-01561-x PMC10493228

[352] H. M. Lee , A. K. Saw , V. K. Morris , et al., “Epigenome Reprogramming Through H3K27 and H3K4 Trimethylation as a Resistance Mechanism to DNA Methylation Inhibition in BRAFV600E‐Mutated Colorectal Cancer,” Clinical Cancer Research 30, no. 22 (2024): 5166–5179. 39269307 10.1158/1078-0432.CCR-24-1166 PMC11829253

[353] S. Lai , Y. Su , C. Chi , et al., “DNMT3b/OCT4 expression Confers Sorafenib Resistance and Poor Prognosis of Hepatocellular Carcinoma Through IL‐6/STAT3 Regulation,” Journal of Experimental & Clinical Cancer Research 38, no. 1 (2019): 474. 31771617 10.1186/s13046-019-1442-2 PMC6878666

[354] R. Plummer , L. Vidal , M. Griffin , et al., “Phase I Study of MG98, an Oligonucleotide Antisense Inhibitor of human DNA Methyltransferase 1, Given as a 7‐day Infusion in Patients With Advanced Solid Tumors,” Clinical Cancer Research 15, no. 9 (2009): 3177–3183. 19383817 10.1158/1078-0432.CCR-08-2859

[355] M. B. Pappalardi , K. Keenan , M. Cockerill , et al., “Discovery of a First‐in‐class Reversible DNMT1‐selective Inhibitor With Improved Tolerability and Efficacy in Acute Myeloid Leukemia,” Nat Cancer 2, no. 10 (2021): 1002–1017. 34790902 PMC8594913

[356] T. Xu , Y. Fang , Y. Gu , et al., “HDAC Inhibitor SAHA Enhances Antitumor Immunity via the HDAC1/JAK1/FGL1 Axis in Lung Adenocarcinoma,” Journal for ImmunoTherapy of Cancer 12, no. 10 (2024): e010077. 39384195 10.1136/jitc-2024-010077 PMC11474878

[357] Z. Yang , W. Su , Q. Zhang , et al., “Lactylation of HDAC1 Confers Resistance to Ferroptosis in Colorectal Cancer,” Adv Sci (Weinh) 12, no. 12 (2025): e2408845. 39888307 10.1002/advs.202408845 PMC11947995

[358] C. Mayr , T. Kiesslich , S. Erber , et al., “HDAC Screening Identifies the HDAC Class i Inhibitor romidepsin as a Promising Epigenetic Drug for Biliary Tract Cancer,” Cancers (Basel) 13, no. 15 (2021): 3862. 34359763 10.3390/cancers13153862 PMC8345689

[359] J. Lobo , C. Guimaraes‐Teixeira , D. Barros‐Silva , et al., “Efficacy of HDAC Inhibitors Belinostat and Panobinostat Against Cisplatin‐Sensitive and Cisplatin‐Resistant Testicular Germ Cell Tumors,” Cancers (Basel) 12, no. 10 (2020): 2903. 33050470 10.3390/cancers12102903 PMC7601457

[360] T. T. T. Nguyen , Y. Zhang , E. Shang , et al., “HDAC Inhibitors Elicit Metabolic Reprogramming by Targeting Super‐enhancers in Glioblastoma Models,” Journal of Clinical Investigation 130, no. 7 (2020): 3699–3716. 32315286 10.1172/JCI129049 PMC7324177

[361] Y. Geng , J. Liu , Y. Xie , et al., “Trichostatin a Promotes GLI1 Degradation and P21 Expression in Multiple Myeloma Cells,” Cancer Manag Res 10 (2018): 2905–2914. 30214285 10.2147/CMAR.S167330 PMC6118243

[362] M. Fournel , C. Bonfils , Y. Hou , et al., “MGCD0103, a Novel Isotype‐selective Histone Deacetylase Inhibitor, Has Broad Spectrum Antitumor Activity in Vitro and in Vivo,” Molecular Cancer Therapeutics 7, no. 4 (2008): 759–768. 18413790 10.1158/1535-7163.MCT-07-2026

[363] V. Marquardt , J. Theruvath , D. Pauck , et al., “Tacedinaline (CI‐994), a Class I HDAC Inhibitor, Targets Intrinsic Tumor Growth and Leptomeningeal Dissemination in MYC‐driven Medulloblastoma While Making Them Susceptible to Anti‐CD47‐induced Macrophage Phagocytosis via NF‐kB‐TGM2 Driven Tumor Inflammation,” Journal for ImmunoTherapy of Cancer 11, no. 1 (2023): e005871. 36639156 10.1136/jitc-2022-005871 PMC9843227

[364] S. Rai , W. S. Kim , K. Ando , et al., “Oral HDAC Inhibitor Tucidinostat in Patients With Relapsed or Refractory Peripheral T‐cell Lymphoma: Phase IIb Results,” Haematologica 108, no. 3 (2023): 811–821. 36200417 10.3324/haematol.2022.280996 PMC9973490

[365] M. Yamagishi , Y. Kuze , S. Kobayashi , et al., “Mechanisms of Action and Resistance in Histone Methylation‐targeted Therapy,” Nature 627, no. 8002 (2024): 221–228. 38383791 10.1038/s41586-024-07103-x PMC10917674

[366] F. Ferrari , L. Arrigoni , H. Franz , et al., “DOT1L‐mediated Murine Neuronal Differentiation Associates With H3K79me2 Accumulation and Preserves SOX2‐enhancer Accessibility,” Nature Communications 11, no. 1 (2020): 5200. 10.1038/s41467-020-19001-7 PMC7562744 33060580

[367] W. Ma , C. Han , J. Zhang , et al., “The Histone Methyltransferase g9a Promotes Cholangiocarcinogenesis Through Regulation of the Hippo Pathway Kinase LATS2 and YAP Signaling Pathway,” Hepatology 72, no. 4 (2020): 1283–1297. 31990985 10.1002/hep.31141 PMC7384937

[368] Y. Jiang , Y. Yuan , M. Chen , et al., “PRMT5 disruption Drives Antitumor Immunity in Cervical Cancer by Reprogramming T Cell‐mediated Response and Regulating PD‐L1 Expression,” Theranostics 11, no. 18 (2021): 9162–9176. 34522232 10.7150/thno.59605 PMC8419032

[369] K. Wang , J. Luo , S. Yeh , et al., “The MAO Inhibitors Phenelzine and Clorgyline Revert Enzalutamide Resistance in Castration Resistant Prostate Cancer,” Nature Communications 11, no. 1 (2020): 2689. 10.1038/s41467-020-15396-5 PMC7264333 32483206

[370] R. Dou , L. Han , C. Yang , et al., “Upregulation of LINC00501 by H3K27 Acetylation Facilitates Gastric Cancer Metastasis Through Activating Epithelial‐mesenchymal Transition and Angiogenesis,” Clinical and translational medicine 13, no. 10 (2023): e1432. 37867401 10.1002/ctm2.1432 PMC10591115

[371] Y. Su , Z. Li , Q. Li , et al., “Oncofetal TRIM71 Drives Liver Cancer Carcinogenesis Through Remodeling CEBPA‐mediated Serine/Glycine Metabolism,” Theranostics 14, no. 13 (2024): 4948–4966. 39267787 10.7150/thno.99633 PMC11388079

[372] K. T. Siu , J. Ramachandran , A. J. Yee , et al., “Preclinical Activity of CPI‐0610, a Novel Small‐molecule Bromodomain and Extra‐terminal Protein Inhibitor in the Therapy of Multiple Myeloma,” Leukemia 31, no. 8 (2017): 1760–1769. 27890933 10.1038/leu.2016.355

[373] D. W. Cescon , J. Hilton , S. Morales Murilo , et al., “A Phase I/II Study of GSK525762 Combined With Fulvestrant in Patients With Hormone Receptor‐positive/HER2‐negative Advanced or Metastatic Breast Cancer,” Clinical Cancer Research 30, no. 2 (2024): 334–343. 37992310 10.1158/1078-0432.CCR-23-0133 PMC10792358

[374] Y. Pang , G. Bai , J. Zhao , et al., “The BRD4 Inhibitor JQ1 Suppresses Tumor Growth by Reducing c‐Myc Expression in Endometrial Cancer,” Journal of translational medicine 20, no. 1 (2022): 336. 35902869 10.1186/s12967-022-03545-x PMC9331486

[375] J. Jin , J. Luo , X. Jin , et al., “Chromatin Helicase CHD6 Establishes Proinflammatory Enhancers and Is a Synthetic Lethal Target in FH‐Deficient Renal Cell Carcinoma,” Cancer Research 85, no. 4 (2025): 675–691. 39589780 10.1158/0008-5472.CAN-24-0787

[376] J. Cantley , X. Ye , E. Rousseau , et al., “Selective PROTAC‐mediated Degradation of SMARCA2 Is Efficacious in SMARCA4 Mutant Cancers,” Nature Communications 13, no. 1 (2022): 6814. 10.1038/s41467-022-34562-5 PMC9649729 36357397

[377] R. Rakesh , U. B. Chanana , S. Hussain , et al., “Altering Mammalian Transcription Networking With ADAADi: An Inhibitor of ATP‐dependent Chromatin Remodeling,” PLoS ONE 16, no. 5 (2021): e251354. 10.1371/journal.pone.0251354 PMC8128233 33999958

[378] M. W. Wong‐Brown , A. van der Westhuizen , and N. A. Bowden , “Sequential Azacitidine and Carboplatin Induces Immune Activation in Platinum‐resistant High‐grade Serous Ovarian Cancer Cell Lines and Primes for Checkpoint Inhibitor Immunotherapy,” BMC cancer 22, no. 1 (2022): 100. 35073851 10.1186/s12885-022-09197-w PMC8787901

[379] R. R. Weng , H. Lu , C. Lin , et al., “Epigenetic Modulation of Immune Synaptic‐cytoskeletal Networks Potentiates Gammadelta T Cell‐mediated Cytotoxicity in Lung Cancer,” Nature Communications 12, no. 1 (2021): 2163. 10.1038/s41467-021-22433-4 PMC8042060 33846331

[380] H. Li , H. J. Jang , K. Rohena‐Rivera , et al., “RNA Mis‐splicing Drives Viral Mimicry Response After DNMTi Therapy in SETD2‐mutant Kidney Cancer,” Cell reports 42, no. 1 (2023): 112016. 36662621 10.1016/j.celrep.2023.112016 PMC10034851

[381] Y. Zhang , H. Zhao , W. Deng , J. Lai , K. Sang , and Q. Chen , “Zebularine Potentiates Anti‐tumor Immunity by Inducing Tumor Immunogenicity and Improving Antigen Processing Through cGAS‐STING Pathway,” Communications Biology 7, no. 1 (2024): 587. 38755254 10.1038/s42003-024-06271-w PMC11099016

[382] H. D. Bear , X. Deng , D. Bandyopadhyay , et al., “T‐cell Immune Checkpoint Inhibition plus Hypomethylation for Locally Advanced HER2‐negative Breast Cancer: A Phase 2 Neoadjuvant Window Trial of decitabine and pembrolizumab Followed by Standard Neoadjuvant Chemotherapy,” Journal for ImmunoTherapy of Cancer 13, no. 2 (2025): e010294. 40021215 10.1136/jitc-2024-010294 PMC11873355

[383] J. Shim , J. Ryu , S. Y. Jeong , et al., “Combination Effect of Poly (ADP‐ribose) Polymerase Inhibitor and DNA Demethylating Agents for Treatment of Epithelial Ovarian Cancer,” Gynecologic Oncology 165, no. 2 (2022): 270–280. 35305818 10.1016/j.ygyno.2022.03.005

[384] X. Sun , C. Bai , H. Li , et al., “PARP1 modulates METTL3 Promoter Chromatin Accessibility and Associated LPAR5 RNA M(6)A Methylation to Control Cancer Cell Radiosensitivity,” Molecular Therapy 31, no. 9 (2023): 2633–2650. 37482682 10.1016/j.ymthe.2023.07.018 PMC10492194

[385] R. Abbotts , M. J. Topper , C. Biondi , et al., “DNA Methyltransferase Inhibitors Induce a BRCAness Phenotype That Sensitizes NSCLC to PARP Inhibitor and Ionizing Radiation,” PNAS 116, no. 45 (2019): 22609–22618. 31591209 10.1073/pnas.1903765116 PMC6842607

[386] M. Ullrich , S. Richter , J. Liers , et al., “Epigenetic Drugs in Somatostatin Type 2 Receptor Radionuclide Theranostics and Radiation Transcriptomics in Mouse Pheochromocytoma Models,” Theranostics 13, no. 1 (2023): 278–294. 36593963 10.7150/thno.77918 PMC9800739

[387] J. A. I. Thoms , F. Yan , H. R. Hampton , et al., “Clinical Response to Azacitidine in MDS Is Associated With Distinct DNA Methylation Changes in HSPCs,” Nature Communications 16, no. 1 (2025): 4451. 10.1038/s41467-025-59796-x PMC12075701 40360497

[388] J. Cany , M. W. H. Roeven , J. S. Hoogstad‐Van Evert , et al., “Decitabine Enhances Targeting of AML Cells by CD34(+) Progenitor‐derived NK Cells in NOD/SCID/IL2Rg(null) Mice,” Blood 131, no. 2 (2018): 202–214. 29138222 10.1182/blood-2017-06-790204 PMC5757681

[389] E. Estey , R. L. Levine , and B. Löwenberg , “Current Challenges in Clinical Development of, “Targeted Therapies”: The Case of Acute Myeloid Leukemia,” Blood 125, no. 16 (2015): 2461–2466. 25762181 10.1182/blood-2015-01-561373

[390] M. A. Dawson , G. Borthakur , B. J. P. Huntly , et al., “A Phase I/II Open‐Label Study of molibresib for the Treatment of Relapsed/Refractory Hematologic Malignancies,” Clinical cancer research: an official journal of the American Association for Cancer Research 29, no. 4 (2023): 711–722. 36350312 10.1158/1078-0432.CCR-22-1284 PMC9932578

[391] Z. Chen , Z. Cao , Y. Wang , et al., “Repressing MYC by Targeting BET Synergizes With Selective Inhibition of PI3Kα Against B Cell Lymphoma,” Cancer Letters 524 (2022): 206–218. 34688842 10.1016/j.canlet.2021.10.022

[392] P. Fonseca , W. Cui , N. Struyf , et al., “A Phenotypic Screening Approach to Target p60AmotL2‐expressing Invasive Cancer Cells,” Journal of experimental & clinical cancer research: CR 43, no. 1 (2024): 107. 38594748 10.1186/s13046-024-03031-w PMC11003180

[393] G. Xu , S. Chhangawala , E. Cocco , et al., “ARID1A determines Luminal Identity and Therapeutic Response in Estrogen‐receptor‐positive Breast Cancer,” Nature Genetics 52, no. 2 (2020): 198–207. 31932695 10.1038/s41588-019-0554-0 PMC7341683

[394] B. G. Bitler , K. M. Aird , A. Garipov , et al., “Synthetic Lethality by Targeting EZH2 Methyltransferase Activity in ARID1A‐mutated Cancers,” Nature Medicine 21, no. 3 (2015): 231–238. 10.1038/nm.3799 PMC4352133 25686104

[395] J. Lv , X. Yu , X. Liu , et al., “The LncRNA STEAP3‐AS1 Promotes Liver Metastasis in Colorectal Cancer by Regulating Histone Lactylation Through Chromatin Remodelling,” Journal of experimental & clinical cancer research: CR 44, no. 1 (2025): 205. 40665344 10.1186/s13046-025-03461-0 PMC12261760

[396] L. Xiong , Y. Xu , Z. Gao , et al., “A Patient‐derived Organoid Model Captures Fetal‐Like Plasticity in Colorectal Cancer,” Cell Research 35, no. 9 (2025): 642–655. 40615635 10.1038/s41422-025-01139-y PMC12408841

[397] E. Kim , Y. Kim , Z. Ji , et al., “ROR Activation by Nobiletin Enhances Antitumor Efficacy via Suppression of IκB/NF‐κB Signaling in Triple‐negative Breast Cancer,” Cell death & disease 13, no. 4 (2022): 374. 35440077 10.1038/s41419-022-04826-5 PMC9018867

[398] C. Pan , S. Shao , Y. Gu , and Q. Ni , “Radiation Prevents Tumor Progression by Inhibiting the miR‑93‑5p/EphA4/NF‑κB Pathway in Triple‑Negative Breast Cancer,” Oncology Reports 49, no. 4 (2023): 78. 36866759 10.3892/or.2023.8515 PMC10018453

[399] B. Ku , D. Eisenbarth , S. Baek , et al., “PRMT1 promotes Pancreatic Cancer Development and Resistance to Chemotherapy,” Cell reports Medicine 5, no. 3 (2024): 101461. 38460517 10.1016/j.xcrm.2024.101461 PMC10983040

[400] Y. Isshiki , X. Chen , M. Teater , et al., “EZH2 inhibition Enhances T Cell Immunotherapies by Inducing Lymphoma Immunogenicity and Improving T Cell Function,” Cancer Cell 43, no. 1 (2025): 49–68. 39642889 10.1016/j.ccell.2024.11.006 PMC11732734

[401] L. Zhou , T. Mudianto , X. Ma , R. Riley , and R. Uppaluri , “Targeting EZH2 Enhances Antigen Presentation, Antitumor Immunity, and Circumvents Anti‐PD‐1 Resistance in Head and Neck Cancer,” Clinical cancer research: an official journal of the American Association for Cancer Research 26, no. 1 (2020): 290–300. 31562203 10.1158/1078-0432.CCR-19-1351 PMC6942613

[402] J. Huang , Q. Yin , Y. Wang , et al., “EZH2 inhibition Enhances PD‐L1 Protein Stability Through USP22‐Mediated Deubiquitination in Colorectal Cancer,” Advanced science (Weinheim, Baden‐Wurttemberg, Germany) 11, no. 23 (2024): e2308045. 38520088 10.1002/advs.202308045 PMC11187912

[403] C. M. Bowen , F. Duzagac , A. Martel‐Martel , et al., “Inhibition of Histone Methyltransferase EZH2 for Immune Interception of Colorectal Cancer in Lynch syndrome,” JCI Insight 10, no. 6 (2025): e177545. 39946195 10.1172/jci.insight.177545 PMC11949072

[404] C. Mcguckin , N. Forraz , C. Milet , et al., “Colorectal Cancer Hepatic Metastasis Modeling by Advanced 3D Bioprinting Allows Demonstration of Oncolytic Viral Chemotherapeutic Delivery,” Cancers (Basel) 17, no. 22 (2025): 3705. 41301067 10.3390/cancers17223705 PMC12651912

[405] Y. Xu , L. Yang , G. Li , and C. Rao , “The Role of NF‐κB/MIR155HG in Regulating the Stemness and Radioresistance in Breast Cancer Stem Cells,” Frontiers in bioscience (Landmark edition) 30, no. 1 (2025): 25810. 39862080 10.31083/FBL25810

[406] Y. Li , D. Zhang , M. Yang , et al., “ScBridge Embraces Cell Heterogeneity in Single‐cell RNA‐seq and ATAC‐seq Data Integration,” Nature Communications 14, no. 1 (2023): 6045. 10.1038/s41467-023-41795-5 PMC10539354 37770437

[407] Z. Yu , Y. Lv , C. Su , et al., “Integrative Single‐Cell Analysis Reveals Transcriptional and Epigenetic Regulatory Features of Clear Cell Renal Cell Carcinoma,” Cancer Research 83, no. 5 (2023): 700–719. 36607615 10.1158/0008-5472.CAN-22-2224 PMC9978887

[408] A. Chen , S. Liao , M. Cheng , et al., “Spatiotemporal Transcriptomic Atlas of Mouse Organogenesis Using DNA Nanoball‐patterned Arrays,” Cell 185, no. 10 (2022): 1777–1792. 35512705 10.1016/j.cell.2022.04.003

[409] C. Xia , D. Colognori , X. S. Jiang , K. Xu , and J. A. Doudna , “Single‐molecule Live‐cell RNA Imaging With CRISPR‐Csm,” Nature Biotechnology (2025). 10.1038/s41587-024-02540-5 PMC12700784 39966655

[410] M. Bartosovic , M. Kabbe , and G. Castelo‐Branco , “Single‐cell CUT&Tag Profiles Histone Modifications and Transcription Factors in Complex Tissues,” Nature Biotechnology 39, no. 7 (2021): 825–835. 10.1038/s41587-021-00869-9 PMC7611252 33846645

[411] Y. Deng , M. Bartosovic , P. Kukanja , et al., “Spatial‐CUT&Tag: Spatially Resolved Chromatin Modification Profiling at the Cellular Level,” Science 375, no. 6581 (2022): 681–686. 35143307 10.1126/science.abg7216 PMC7612972

[412] S. Ji , L. Feng , Z. Fu , et al., “Pharmaco‐proteogenomic Characterization of Liver Cancer Organoids for Precision Oncology,” Science Translational Medicine 15, no. 706 (2023): g3358. 10.1126/scitranslmed.adg3358 PMC10949980 37494474

[413] K. P. Guillen , M. Fujita , A. J. Butterfield , et al., “A human Breast Cancer‐derived Xenograft and Organoid Platform for Drug Discovery and Precision Oncology,” Nat Cancer 3, no. 2 (2022): 232–250. 35221336 10.1038/s43018-022-00337-6 PMC8882468

[414] N. Guedeney , M. Cornu , F. Schwalen , C. Kieffer , and A. S. Voisin‐Chiret , “PROTAC Technology: A New Drug Design for Chemical Biology With Many Challenges in Drug Discovery,” Drug Discov Today 28, no. 1 (2023): 103395. 36228895 10.1016/j.drudis.2022.103395

[415] J. Salami and C. M. Crews , “Waste Disposal‐An Attractive Strategy for Cancer Therapy,” Science 355, no. 6330 (2017): 1163–1167. 28302825 10.1126/science.aam7340

[416] S. Kregel , C. Wang , X. Han , et al., “Androgen Receptor Degraders Overcome Common Resistance Mechanisms Developed During Prostate Cancer Treatment,” Neoplasia 22, no. 2 (2020): 111–119. 31931431 10.1016/j.neo.2019.12.003 PMC6957805

[417] J. Hu , B. Hu , M. Wang , et al., “Discovery of ERD‐308 as a Highly Potent Proteolysis Targeting Chimera (PROTAC) Degrader of Estrogen Receptor (ER),” Journal of Medicinal Chemistry 62, no. 3 (2019): 1420–1442. 30990042 10.1021/acs.jmedchem.8b01572

[418] Y. Sun , N. Ding , Y. Song , et al., “Degradation of Bruton's Tyrosine Kinase Mutants by PROTACs for Potential Treatment of Ibrutinib‐resistant non‐Hodgkin Lymphomas,” Leukemia 33, no. 8 (2019): 2105–2110. 30858551 10.1038/s41375-019-0440-x

[419] K. Raina , J. Lu , Y. Qian , et al., “PROTAC‐induced BET Protein Degradation as a Therapy for Castration‐resistant Prostate Cancer,” PNAS 113, no. 26 (2016): 7124–7129. 27274052 10.1073/pnas.1521738113 PMC4932933

[420] S. Zeng , W. Huang , X. Zheng , et al., “Proteolysis Targeting Chimera (PROTAC) in Drug Discovery Paradigm: Recent Progress and Future Challenges,” European Journal of Medicinal Chemistry 210 (2021): 112981. 33160761 10.1016/j.ejmech.2020.112981

[421] S. Chen , Y. Zheng , B. Liang , et al., “The Application of PROTAC in HDAC,” European Journal of Medicinal Chemistry 260 (2023): 115746. 37607440 10.1016/j.ejmech.2023.115746

[422] M. Laurent , M. Geoffroy , G. Pavani , and S. Guiraud , “CRISPR‐Based Gene Therapies: From Preclinical to Clinical Treatments,” Cells 13, no. 10 (2024): 800. 38786024 10.3390/cells13100800 PMC11119143

[423] F. Liu , M. Xin , H. Feng , et al., “Cryo‐shocked Tumor Cells Deliver CRISPR‐Cas9 for Lung Cancer Regression by Synthetic Lethality,” Science Advances 10, no. 13 (2024): k8264. 10.1126/sciadv.adk8264 PMC10980270 38552011

[424] M. Pirouzfar , F. Amiri , M. Dianatpour , and M. A. Takhshid , “CRISPR/Cas9‐mediated Knockout of MLL5 Enhances Apoptotic Effect of Cisplatin in HeLa Cells in Vitro,” EXCLI journal 19 (2020): 170–182. 32194363 10.17179/excli2019-1957 PMC7068203

[425] H. Shaath , R. Vishnubalaji , R. Elango , S. Khattak , and N. M. Alajez , “Single‐cell Long Noncoding RNA (lncRNA) Transcriptome Implicates MALAT1 in Triple‐negative Breast Cancer (TNBC) Resistance to Neoadjuvant Chemotherapy,” Cell Death Discov 7, no. 1 (2021): 23. 33495450 10.1038/s41420-020-00383-y PMC7835365

[426] S. Lee , Y. Kim , and H. J. Ahn , “Systemic Delivery of CRISPR/Cas9 to Hepatic Tumors for Cancer Treatment Using Altered Tropism of Lentiviral Vector,” Biomaterials 272 (2021): 120793. 33836291 10.1016/j.biomaterials.2021.120793

[427] T. Morimoto , T. Nakazawa , R. Matsuda , et al., “CRISPR‐Cas9‐Mediated TIM3 Knockout in human Natural Killer Cells Enhances Growth Inhibitory Effects on human Glioma Cells,” International Journal of Molecular Sciences 22, no. 7 (2021): 3489. 33800561 10.3390/ijms22073489 PMC8036491

[428] Z. Yang , X. Liu , J. Zhu , et al., “Inhibiting Intracellular CD28 in Cancer Cells Enhances Antitumor Immunity and Overcomes Anti‐PD‐1 Resistance via Targeting PD‐L1,” Cancer Cell 43, no. 1 (2025): 86–102. 39672166 10.1016/j.ccell.2024.11.008

[429] Y. Du , Y. Liu , J. Hu , X. Peng , and Z. Liu , “CRISPR/Cas9 Systems: Delivery Technologies and Biomedical Applications,” Asian Journal of Pharmaceutical Sciences 18, no. 6 (2023): 100854. 38089835 10.1016/j.ajps.2023.100854 PMC10711398

[430] X. Xu , C. Liu , Y. Wang , et al., “Nanotechnology‐based Delivery of CRISPR/Cas9 for Cancer Treatment,” Advanced Drug Delivery Reviews 176 (2021): 113891. 34324887 10.1016/j.addr.2021.113891

[431] Y. Wang , X. Yu , Y. Gu , et al., “XGraphCDS: An Explainable Deep Learning Model for Predicting Drug Sensitivity From Gene Pathways and Chemical Structures,” Computers in Biology and Medicine 168 (2024): 107746. 38039896 10.1016/j.compbiomed.2023.107746

[432] J. Xu , C. Lu , S. Jin , et al., “Deep Learning‐based Cell‐specific Gene Regulatory Networks Inferred From Single‐cell Multiome Data,” Nucleic Acids Research 53, no. 5 (2025): gkaf138. 40037709 10.1093/nar/gkaf138 PMC11879466

